# An improved bind-n-seq strategy to determine protein-DNA interactions validated using the bacterial transcriptional regulator YipR

**DOI:** 10.1186/s12866-019-1672-7

**Published:** 2020-01-02

**Authors:** Shi-qi An, Miguel A. Valvano, Yan-hua Yu, Jeremy S. Webb, Guillermo Lopez Campos

**Affiliations:** 1 National Biofilms Innovation Centre (NBIC), Biological Sciences, University of Southampton, Southampton, SO17 1BJ UK; 2 Wellcome-Wolfson Institute for Experimental Medicine, Queen’s University Belfast, Belfast, BT9 7BL UK; 3 State Key Laboratory for Conservation and Utilization of Subtropical Agro-bioresources, College of Life Science and Technology, Guangxi University, Nanning, 530004 Guangxi China

**Keywords:** Protein–DNA interactions, Bind-n-seq, Transcription regulator, Gene expression, Virulence, *Xanthomonas*

## Abstract

**Background:**

Interactions between transcription factors and DNA lie at the centre of many biological processes including DNA recombination, replication, repair and transcription. Most bacteria encode diverse proteins that act as transcription factors to regulate various traits. Several technologies for identifying protein–DNA interactions at the genomic level have been developed. Bind-n-seq is a high-throughput in vitro method first deployed to analyse DNA interactions associated with eukaryotic zinc-finger proteins. The method has three steps (i) binding protein to a randomised oligonucleotide DNA target library, (ii) deep sequencing of bound oligonucleotides, and (iii) a computational algorithm to define motifs among the sequences. The classical Bind-n-seq strategy suffers from several limitations including a lengthy wet laboratory protocol and a computational algorithm that is difficult to use. We introduce here an improved, rapid, and simplified Bind-n-seq protocol coupled with a user-friendly downstream data analysis and handling algorithm, which has been optimized for bacterial target proteins. We validate this new protocol by showing the successful characterisation of the DNA-binding specificities of YipR (YajQ interacting protein regulator), a well-known transcriptional regulator of virulence genes in the bacterial phytopathogen *Xanthomonas campestris* pv. *campestris* (*Xcc*).

**Results:**

The improved Bind-n-seq approach identified several DNA binding motif sequences for YipR, in particular the CCCTCTC motif, which were located in the promoter regions of 1320 *Xcc* genes. Informatics analysis revealed that many of these genes regulate functions associated with virulence, motility, and biofilm formation and included genes previously found involved in virulence. Additionally, electromobility shift assays show that YipR binds to the promoter region of XC_2633 in a CCCTCTC motif-dependent manner.

**Conclusion:**

We present a new and rapid Bind-n-seq protocol that should be useful to investigate DNA-binding proteins in bacteria. The analysis of YipR DNA binding using this protocol identifies a novel DNA sequence motif in the promoter regions of target genes that define the YipR regulon.

## Background

Detailed understanding of transcription and its regulation of gene expression is a major focus of biochemists and molecular biologists [1, 2]. Transcription factors (TFs) are proteins that bind to specific regions of the DNA and regulate gene expression in living cells including bacteria [3–5]. Several studies have provided detailed mechanistic insight, which has been extrapolated and simplified into a set of widely held assumptions about the global nature of TF binding in bacteria [3–5]. However, these studies have been limited to a small number of factors at a few genomic locations.

Current technologies to identify protein-DNA interactions at the genomic level include chromatin immunoprecipitation (ChIP) followed by microarray hybridization (ChIP-chip) or high-throughput sequencing (ChIP-seq) [6, 7]. ChIP-chip and ChIP-seq allow genome-wide discovery of protein-DNA interactions, such as transcription factor binding sites and histone modifications. Although highly informative, these methods are limited by the availability of highly specific antibodies, as well as by the number of transcription factors and accessible binding sites available in any particular cell type under any particular environmental condition. Further, yeast and bacterial one-and two-hybrid systems have been described [8–11]. These systems have the advantage of in vivo selection with stringencies that can be experimentally manipulated. In theory, libraries of target sites up to 15 bp in length (10^9^ sequences) could be surveyed; however, usage of libraries larger than 10^7^ sequences has not been reported [12].

More recently, high-throughput approaches to identify protein-DNA interactions have been developed; these techniques include Protein-Binding Microarray (PBM), Cyclical Amplification and Selection of Targets (CAST), Systematic Evolution of Ligands by Exponential Enrichment (SELEX), Serial Analysis of Gene Expression (SAGE) and Bind-n-seq [12–16]. In PBM, proteins bind double-stranded oligonucleotides on a microarray [13]. CAST generally involves several rounds of amplification and purification for each protein and is therefore labour-intensive [14, 15]. Serial SAGE has been applied in certain studies to reduce the cloning burden and the cost to obtain large numbers of sequences [16]. Bind-n-seq is a high throughput method for in vitro analysis of protein–DNA interactions that takes advantage of deep sequencing. Unlike CAST and SELEX, multiple rounds of binding and amplification are unnecessary. Unlike microarrays, Bind-n-seq is not limited to 10-bp binding sites. Further, many binding reactions can be assayed in parallel with barcoded oligonucleotides. However, this method was only used successfully in the analysis of the DNA-binding domains (DBDs) of eukaryotic zinc-finger proteins [12, 17], and the downstream data analysis of the classical method is challenging for general biologists [12, 17].

**Fig. 1:**
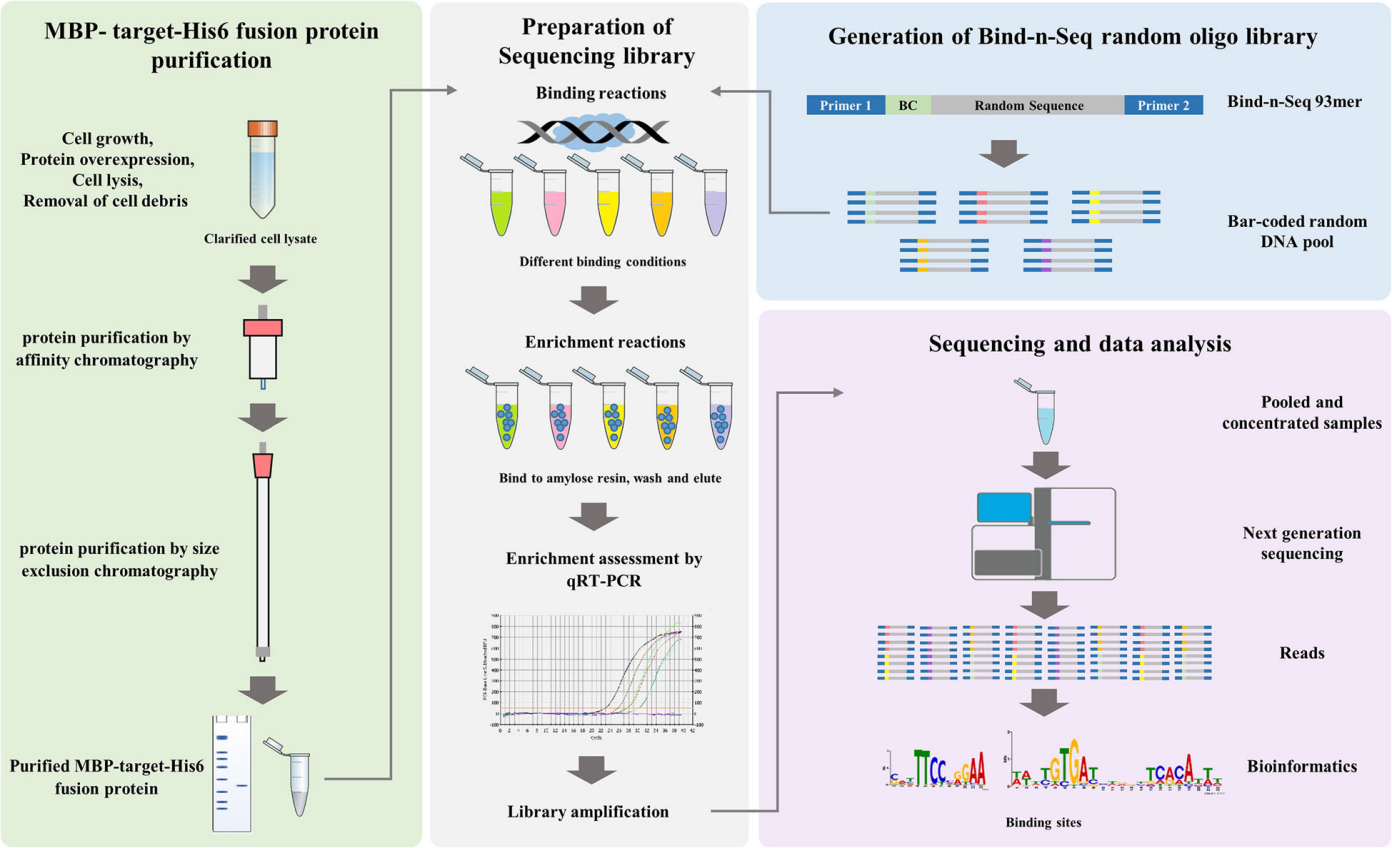
Bind-n-seq experimental overview. The protein purification strategy depends on the properties of the target protein and should be optimized in each case. For YipR, both MBP and His affinity tags were incorporated and an affinity chromatography step was followed by a size exclusion step. After purification, the target protein is assessed for concentration, stability and purity. The protein quality is an essential requirement (green panel left). The Bind-n-seq substrate is an oligo containing constant regions (Primer A and Primer B) a 3-nucleotide bar code (BC) and 21 bp random region (blue panel right). Barcoded oligonucleotides are mixed with various proteins, washed to remove unbound DNA, pooled and sequenced with short read technology (grey panel middle). Reads are sorted by their bar codes and processed through several bioinformatics procedures that result in motifs corresponding to the DNA binding sites of each protein (pink panel right)

We present here an improved, simplified, and comprehensive Bind-n-seq protocol coupled with an easy to use downstream data analysis pipeline. Our improved method enables unbiased, high-throughput and quantitative analysis of broader protein-DNA interactions using the MiSeq system (but can be deployed on other sequencing platforms). The approach involves three steps: (i) binding target protein to randomised oligonucleotide DNA targets, (ii) sequencing the bound oligonucleotides with massively parallel sequencing platform and (iii) finding motifs among the sequences using a computational algorithm (Fig. 1). We demonstrate the utility of Bind-n-seq by analysing the transcriptional regulator YipR (YajQ interacting protein regulator; XC_2801) from *Xanthomonas campestris* pv. *campestris*. Our results identify YipR DNA binding motifs in more than 1000 genes indicating this protein is a global regulator of a large number of genes in *X. campestris*. We also experimentally validate YipR interactions with target DNA containing the main binding motif using mobility gel shift assays. Our new Bind-n-seq method will allow researchers to examine a broad range of transcription factors from both eukaryote and prokaryote and identify the binding site in a more efficient and cost-effective -manner.

## Results

### Overall experimental approach

The success of a Bind-n-seq approach depends on three key elements: the purification of protein(s) of interest and its binding to DNA, the randomisation of the DNA Bind-n-seq oligonucleotide library and a robust data analysis (Fig. 1). We validated this approach by characterising the genes directly controlled by the transcriptional regulator YipR (XC_2801) from the plant pathogen *X. campestris pv. campestris* strain 8004. YipR is a transcriptional regulator carrying CheY-homologous receiver (REC) and DNA-binding domains, which governs virulence gene expression [18]. YipR homologues are present in the genome of most *Xanthomonas* species, but their regulons remain ill defined. Therefore, it is important to understand the extent of the YipR regulon by identifying genes directly regulated by the YipR family of proteins.

### Purification of target protein for the bind-n-seq approach

**Fig. 2:**
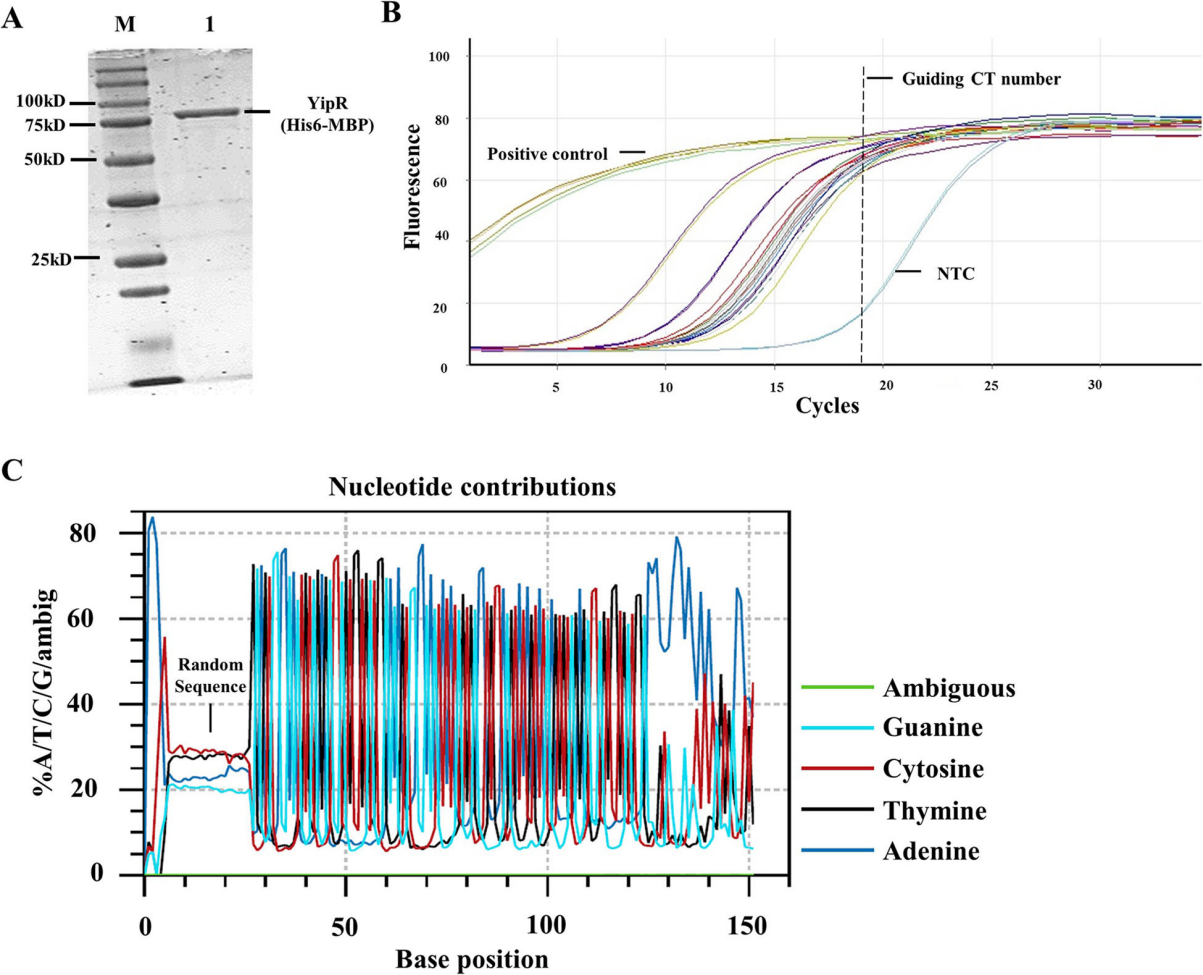
Protein purification of YipR, DNA-protein enrichment and identification of DNA binding motifs for YipR. **a** SDS-PAGE of the YipR protein purified by nickel affinity chromatography shows a single band of the expected size of 81 kDa (**b**) Assessment of enrichment of DNA recovered from Bind-n-seq reactions using real-time PCR. Samples derived from oligo only were used as positive control, No Template Control (NTC) was also included. **c** Quality analysis of synthesised 93-mer oligo

The method of protein expression and purification for a Bind-n-seq experiment must be optimised on case-by-case basis. For the YipR protein, we had success obtaining good quality soluble protein using MBP- and His- dual tagged expression vector, which allowed the expression of YipR in *E. coli* BL21 and purification by affinity and size exclusion chromatography. SDS/PAGE shows that the protein preparation gave a single band of the expected size of ~ 81 kDa (Fig. 2).

### Preparation and evaluation of bind-n-seq reactions

For the binding reaction, purified YipR was mixed with double-stranded Bind-n-seq target oligonucleotides, which contained a 2-nt AA leader, a 3-nt bar code, and a binding region consisting of a 21-bp random and flanking Illumina primer-binding sites. Specifically, a randomised region of 21 bp contained 4.4× 10^12^ combinations (4^21^). Each binding reaction contained approximately 10-fold over-representation of each possible 21-mer, corresponding to 80 pmol or 1600 ng of single-stranded 93-mer oligonucleotides. Additionally, each binding reaction contained more than 10^7^ copies of each possible 10-mer or more than 10^2^ copies of each possible 18-mer. Double-stranded DNAs were created by primer extension. After incubation, the protein-DNA complexes were separated from unbound and low-affinity DNAs and then the bound DNAs were eluted and quantified. For YipR examined protein concentrations ranging from no protein (0 nM YipR) to 4000 nM, which covered and exceed the reported Kd values. The sequence-specific DNA binding affinities of various target proteins (transcription regulator in this case) have been studied in vitro and their apparent Kd values fall within nanomolar or low micromolar ranges [19, 20].

**Fig. 3:**
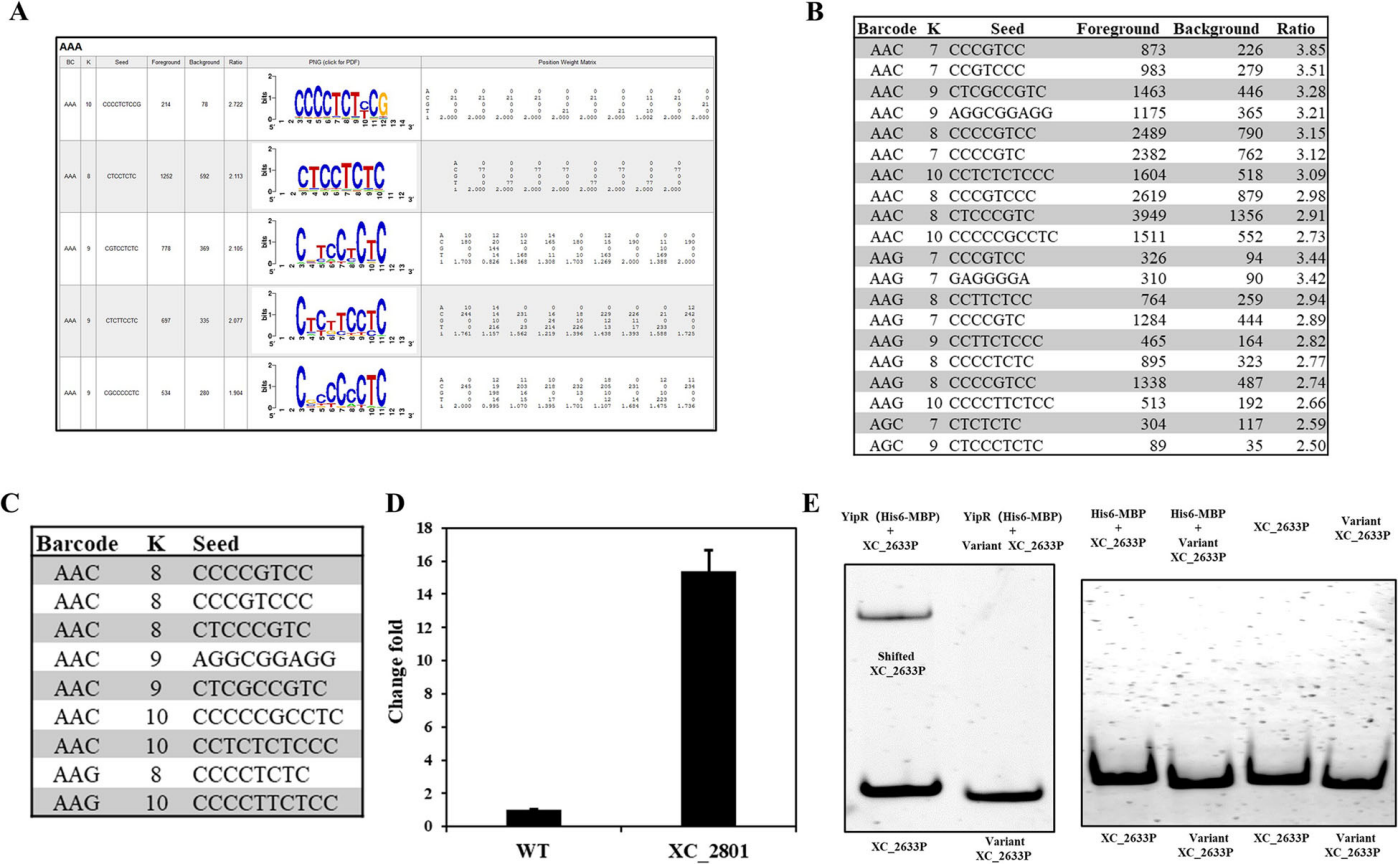
Bind-n-seq reveals binding sites of YipR in the *Xanthomonas campestris.* **a** Representative results generated by generated by MERMADE under barcode AAA. **b** Manual filtering from MERMADE shows enriched motifs (Cut-off 3.0 fold) identified under different binding conditions. **c** The automatic filtering analysis report from MERMADE using Extractmotif package (Cut-off 3.0 fold) shows (**d**) qRT-PCR analysis reveals that mutation of *yipR* in leads to the elevation in expression of *XC_2633* validating previous observations seen using RNA-seq analysis. **e** Binding of YipR to the XC_2633 promoter is modulated by the presence and absence of “CCCTCTC” motif. The impact presence and absence of “CCCTCTC” motif on the binding of YipR to the *XC_2633* promoter was assessed by the use of electromobility shift assay (EMSA). The DIG-labelled promoter fragment was incubated with purified YipR and XC_2633 promoter with or without binding motif. His-MBP tag alone and DNA fragment alone were used as negative control in the assay

Enrichment was achieved using a resin-based method where amylose resin was added to the binding reactions at equilibrium to capture the proteins, then washed three times with a parameter-specific wash buffer. Buffer salt concentrations ranging from no addition of KCl salt (0 mM KCl) to 500 mM were surveyed. Our data showed KCl concentration at 10 nM with 400 nM YipR protein was the best condition for binding, as most reads were identified under this condition (Fig. 3).

### DNA amplification and preparation of sequencing library

After incubation and enrichment, the protein-DNA complexes were separated from unbound and low-affinity DNAs and then the bound DNAs were eluted and checked by Real-time PCR (RT-PCR). RT-PCR was also used to determine the number of cycles required to amplify all output samples that would be sufficient for sequencing (Fig. 2). Samples were analysed on a Rotor-Gene Q RT-PCR platform (Qiagen). The sequencing library was amplified using touchdown sequence method. The PCR products were purified and quantified by QIAquick PCR purification kit (Qiagen) and Qubit dsDNA high sensitivity assay kit (Thermo Fisher Scientific). The DNAs from several enrichment reactions were combined in approximately equal concentrations and concentrated to approximately 50 μl. High throughput sequencing was performed in an Illumina MiSeq platform (Earlham Institute, UK).

### Sequence analysis and in silico binding motif characterisation

The generated sequence data undergoes standard QC analysis. In total 1,610,524 reads with 3-nt barcoded were obtained. Demultiplexed group with AAC barcode contained the highest read number (377,199), while AGC contained the lowest reads number (55,514). The input sequencing file were further analysed for the quality of the synthesised oligo. The ambiguous reading percentage was low and the 21-mer randomized region contains ~ 25% of each type nucleotide (Average: A: 23.3%, T: 27.8%, C: 28.6%, G: 20.2%) (Fig. 2), suggesting the quality of the library was acceptable.

The sequence file was then analysed using MERMADE for motif analysis on the YipR reads. For this analysis, sequences were analysed relative to a file of background sequences using a default settings in MERMADE. A graphical representation of the sequence motifs identified was rendered using WebLogo. It was found that 400 nM protein with 10 mM KCl provided the optimal enrichment for YipR (Barcode AAC) (Fig. 3). Enriched motifs (Ratio > 2.5) were also identified from conditions with Barcode AAG (400 nM protein, 25 mMCl) and Barcode AGC (4000 nM protein, 100 mM KCl) but with significantly less reads. Importantly, there was no enriched motif identified from control conditions.

MERMADE results filtered to eliminate low complexity patterns and those with an enrichment below 2.5-fold over background and foreground reads > 500. We developed the ExtractMotifs package to select the final list of sequences. This script uses the “.html” output generated by MERMADE to rapidly 1) identify all the unique motifs; 2) Identify the shorter unique motifs that might be contained in longer ones; and 3) identify the longer unique motifs.

To filter the low complexity patterns, we demanded that all motifs be enriched 2.5-fold over background. We collected all reads that match the motifs and ran on this subset to arrive at the final motif(s). The list obtained from ExtractMotifs package was then submitted to the Regulatory Sequence Analysis Tools prokaryotes (RSAT) genome-scale DNA-pattern identification. This analysis allowed the identification of the consensus binding sequence for YipR and also located its occurrences in the *X. campestris* genome (Additional file 2: Table S1), to identify putative transcription factor binding sites in upstream sequences of a set of genes.

### YipR regulates the expression of XC_2633 and binds to its promoter region in vitro

We defined in silico 9 potential binding motifs of YipR (Fig. 3). To determine if these motifs are associated to *X. campestris* genes we used the Regulatory Sequence Analysis Tools for prokaryotes (RSAT) to screen the identified DNA-patterns against the *X. campestris* 8004 genome sequence (*X. campestris* GCF 000012105.1 ASM1210v1) limiting the search window to 200 bp upstream of annotated Open reading frames (ORFs) and allowing no overlaps with upstream ORFs (the substitutions option was set at 1).

We identified 2337 hits (102 hits were 100% match) within the promoter region of 1320 *Xanthomonas campestris* genes. Several of these genes including *XC_1391* (hypothetical), *XC_1372* (hypothetical), *XC_2332* (*flgA*), *XC_2234* (*flgB*), *XC_2339* (*flgG*), *XC_2240* (*flgH*), *XC_2251* (RNA polymerase sigma-54 factor), *XC_2277*(*flhB*), *XC_2279*(*flhF*), *XC_2633* (hypothetical) and *XC_2857* (*proU*) were previously shown to be regulated by YipR by RNA-seq and to be involved in virulence [21]. We confirmed that YipR regulates the expression of *XC_2633* using Real-Time Quantitative Reverse Transcription PCR (Real-time qRT-PCR) (Fig. 3). qRT-PCR also confirmed that *XC_1732*, *XC_2239* and *XC_2277* are regulated by YipR, as previously published [21].

We next conducted electrophoretic mobility shift assays (EMSA) to demonstrate that YipR interacts with the *XC_2633* promoter region. Dual-tagged expression constructs of YipR (His6-MBP) and tag alone as a control (His6-MBP) were generated and protein subsequently purified by nickel affinity column chromatography. The purified dual-tagged YipR fusion protein caused a mobility shift when incubated with a DNA fragment spanning the *XC_2633* promoter (Fig. 3**,** Additional file 3: Table S2). However, DNA fragment lacking the CCCTCTC motif showed no shift unless a high concentration of protein was added (Fig. 3**,** Additional file 3: Table S2), while the MBP-tag alone did not bind to the DNA fragments (Fig. 3**,** Additional file 1: Figure S1). Together, the data indicate that YipR controls *XC_2633* expression by binding to the upstream region of *XC_2633* in a manner that requires the CCCTCTC motif, providing experimental validation to the Bind-n-seq experimental approach.

## Discussion

We show that our Bind-n-seq approach can identify in vitro binding site motifs in a one-step enrichment of an oligonucleotide library containing 93-mer sequences. The method is rapid, and the simplified protocol using high-throughput sequencing allows the simultaneous analysis of multiple proteins. Also, we introduced a robust straightforward downstream data analysis and handling algorithm. These conclusions are supported by the identification in silico and experimental validation of a novel preferred DNA binding motif for the *X. campestris* YipR virulence regulator protein.

We envisage our Bind-n-seq approach could accelerate the characterisation of transcription factors with unknown binding sites. The number of transcription factors found within a microorganism increases with its genome size. There are more than 250 proteins in the *X. campestris* genome that contain predicted DNA-binding domains, most of which are expected to be transcription factors [22]. However, the gene targets of most of these proteins are unknown. Identifying these targets is not only useful from a discovery perspective, but also to model the transcription factor binding code and advance understanding of bacterial cell physiology. An accurate transcription factor binding code would not only allow predicting binding sites and identifying regulon components, but will also improve the design of engineered domains for synthetic biology applications and network rewiring.

A similar concept was recently applied to determine sequence motifs for RNAs that bind to a specific RNA-binding protein [23–25]. The method, RNA Bind-n-seq, comprehensively characterises sequence and structural specificity of RNA binding proteins, and it has been applied to characterize developmental alternative splicing factors like RBFOX2 [23]. This platform has also been adapted for rapid screening, quantitative identification of high-affinity binding sites of small molecules that bind to DNA, which provides a better understanding of small molecule-DNA recognition, which will be essential for in vitro applications, such as DNA nanotechnology [26]. The data analysis pipeline used in our improved DNA Bind-n-seq method could also be adopted for downstream analysis of RNA Bind-n-seq experiments.

## Conclusion

We developed an improved Bind-n-seq approach to define potential direct DNA binding functions of the global transcription regulator protein YipR. The novel motifs identified may guide localization of YipR to target genes in vivo, where it can be recruited to regulate gene expression.

The advantages of our improved Bind-n-seq approach are:

- High-throughput and the use of barcoding to allow the simultaneous analysis of multiple protein samples
- Not limited to 10-bp binding sites to allow the investigation of proteins with long DNA binding sites taking advantage of DNA sequencing platforms that can allow for lengths of random DNA over 35 bp
- Easy design and synthesis of random oligo DNA library
- Cost-effective. Parallel DNA sequencing is affordable to most research labs and it can generate over 5 million sequences in a single assay
- User-friendly downstream bioinformatic pipeline by freely available software that requires minimal training

The limitations of the technique include:

- Bind-n-seq cannot detect the interaction of specific proteins with specific genomic regions in vivo. Therefore, experimental validation is required.
- The Bind-n-seq approach relies on the ability to detect protein-dependent binding motifs from a background of random sequences. However, if the background is not perfectly random, motifs could appear to arise due to background bias.
- The relative binding affinity is calculated with an abundance of sequences in total sequenced reads. Therefore, some low-affinity sequences may be undetected.
- PCR use during certain steps of the approach may introduce bias or artefacts. Despite sequencing errors are substantially reduced but are still present.

## Methods

### Protein expression

The coding region of the target gene encoding the transcriptional regulator YipR (XC_2801) from *X. campestris* pv. *campestris,* was sub-cloned into the vector pMAL-c5x, which enables the expression of a protein fused with both 6xHis tag (C-terminal) and the maltose binding protein (MBP) tag (N-terminal). The N-terminal MBP domain improves the solubility of the expressed proteins and the His-tag allows for standard large-scale protein purification by Ni ^2+^ − affinity chromatography using an automated system.

A 1-ml overnight culture was used to inoculate 50 ml of fresh LB medium in a 250-ml culture flask supplemented with 50 μg/ml ampicillin. This flask was incubated with shaking (200 rpm) at 37 °C overnight (~ 16 h). A 20 ml of the overnight culture was used to inoculate 1 L of fresh LB medium in a 2.5 L culture flask supplemented with 50 μg/ml ampicillin and incubated with shaking (200 rpm) at 37 °C until the culture attains OD600 = 0.4–0.6 (~ 3 h). Expression was induced by adding 60 μl of 0.5 M IPTG to a final concentration of 0.3 mM IPTG. Shaking is continued at 18 °C overnight (~ 16 h). Cells were harvested by centrifugation at 4000 rpm, 4 °C for 30 min and the supernatant discarded. These samples can be stored indefinitely at − 80 °C or used directly for protein purification.

### Protein purification by affinity chromatography

The cell culture pellets were re-suspended with 50 ml lysis buffer (100 mM Tris-HCl [pH 8], 20 mM, Imidazole, 500 mM NaCl, 1 mM TCEP-HCl (Tris(2-carboxyethyl)phosphine hydrochloride), 2% (V/V) Glycerol), supplemented with 1 ml lysozyme (50 mg/ml), 50 μl DNase I (5 mg/ml) and one tablet of protease inhibitor. Bacterial cells were lysed with a microfluidizer or French Press at ~ 20,000 psi. Lysis was considered complete when the cloudy cell suspension becomes translucent. The lysate was centrifuged for 30 min at 16,000 rpm at 4 °C. Soluble protein (supernatant) was removed into a fresh 50 ml centrifuge tube. The supernatant was then filtered through a 0.22 μm filter and kept on ice. Affinity chromatography purification was performed using a HisTrap™ FF column (5 ml) in the ÄKTA protein purification system. The column was washed with Wash buffer 1 (100 mM Tris-HCl [pH 8], 20 mM Imidazole, 2 M NaCl, 2% Glycerol, 1 mM TCEP-HCl, 0.1. mM AEBSF (4-(2-Aminoethyl)benzenesulfonyl fluoride hydrochloride)) to remove nonspecifically bound DNA. Then the column was washed using Wash buffer 2 (100 mM Tris-HCl [pH 8], 20 mM Imidazole, 50 mM NaCl, 2% Glycerol, 1 mM TCEP-HCl, 0.1 mM AEBSF). Elution was carried out with Elution buffer 1 (100 mM Tris-HCl [pH 8], 500 mM Imidazole, 500 mM NaCl, 2% Glycerol, 1 mM TCEP-HCl, 0.1 mM AEBSF) using a linear gradient with a set target concentration of Elution buffer 1 of 50%. Protein-containing fractions were run on a 12% polyacrylamide gel. Visualization of protein bands was achieved by incubating the gel with InstantBlue stain for 5–10 min and the protein-containing fractions pooled. The protein sample was stored at 4 °C.

### Protein purification by size exclusion chromatography

The protein sample was transferred into 20 ml ultrafiltration spin column (10,000 MWCO) and centrifuged at 4000 rpm at 4 °C until the final volume reached approximately 5 ml. Size exclusion chromatography purification was performed using HiLoad 16/600 Superdex 75 prep grade column with ÄKTA protein purification system using Binding buffer A (20 mM Tris-HCl [pH 8], 50 mM KCl, 2% Glycerol, 1 mM TCEP-HCl, 1 mM EDTA). Protein-containing fractions were run on a 12% polyacrylamide gel. Visualization of protein bands was achieved by incubating the gel with Instant blue stain for 5–10 min. Protein-containing fractions were pooled to together and concentration determined using a protein assay kit (BioRad DC protein assay kit).

### Bind-n-seq: barcodes assignment and equilibration reactions

Barcodes were assigned to each testing condition as shown in Additional file 4: Table S3**.** Primer extension PCR master mix was generated by added randomized oligos for 15 reactions (25 μl/rxn): 52.5 μl of H_2_O, 15 μl of 10 μM Primer 1 (Additional file 5: Table S4), 187.5 μl of Taq DNA polymerase master mix (2×). A volume of 17 μl of the master mix was added into each PCR tube or well of a PCR microplate. 8 μl of 10 μM Bind-n-seq 93 mer (Additional file 5: Table S4) was added to each PCR reaction. PCR was run on a thermal cycler and using the following PCR program: [95 °C for 2 min] × 1, [63 °C for 1 min] × 1, [72 °C for 4 min] × 1, and store at 4 °C.

### Bind-n-seq: binding reactions

For binding reaction, 20 × Binding buffer A (without KCl) was prepared as follows: 400 mM Tris-HCl, 20 mM TCEP-HCl, 40% Glycerol, 20 mM EDTA, and H_2_O to bring up the final volume to 100 ml. A master mix of Binding buffer B was prepared as follows for 12 reactions: A volume of 30 μl of 20 × Binding Buffer A (without KCl), 6 μl of 1 M MgCl2, 60 μl of 10% BSA and 24 μl of H_2_O. The KCl salt solutions were prepared as shown in Additional file 6: Table S5. Highly purified proteins were diluted to a concentration of 40 μM in Binding buffer A. A volume of 10 μl Binding buffer B was added to the Oligo mixture (25 μl) described above. Then protein (5 μl) and salt solution (10 μl) were added to the reaction tubes as shown in Additional file 7: Table S6 to make a total volume of 50 μl. The reaction tubes were incubated at room temperature for 2 h.

### Bind-n-seq: enrichment reactions

Bind-n-seq wash buffers were prepared using different concentrations, as described in Additional file 8: Table S7. A 1.5 ml sterile microcentrifuge tube containing each binding reaction condition was prepared. A volume of 100 μl of the amylose resin slurry (≈ 50 μl packed resin after spinning down) was added to each microcentrifuge tube, and then centrifuged for 1 min at 14,000 rpm at room temperature. The supernatant was carefully removed without disturbing the resin. A volume of 1 ml H_2_O was added to the amylose resin and vortexed for 30 s. These H_2_O washes were repeated three times. Then a volume of 1 ml Bind-n-seq wash buffer (Additional file 8: Table S7) with specific KCl concentration to the corresponding tubes to equilibrate the resin was added. The tube was centrifuged for 1 min at 14,000 rpm at room temperature. The supernatant was carefully removed without disturbing the resin. This wash was repeated using Bind-n-seq wash buffer. A volume of 50 μl protein-DNA reaction was added to the equilibrated resin and incubated at room temperature for 30 min (the solution was gently mixed every 10 min). The tubes were centrifuged for 1 min at 14,000 rpm at room temperature and the supernatant was removed without disturbing the resin. Again, a 1 ml volume of Bind-n-seq wash buffer with specific KCl concentration was added to the corresponding tubes to remove the unbound nucleotides. These tubes were included for 10 min at room temperature and then centrifuged at 14,000 rpm at room temperature for 1 min. The wash step was repeated twice with Bind-n-seq wash buffer. After the washed a volume of 50 μl Bind-n-seq elution buffer was added (10 mM maltose in 1 ml EB buffer (QIAquick PCR purification kit, Qiagen)) to the reaction tubes to elute bound nucleotides and incubated for 10 min at room temperature. After incubation, the tubes were centrifuged for 1 min at 14,000 rpm at room temperature. The supernatant was transferred to a new microcentrifuge tube and stored at − 20 °C for up to 2 weeks (or used immediately for library amplification).

### Bind-n-seq: library amplification

The qPCR master mix was created for 15 reactions to assess enrichment of recovered DNA (20 μl per reaction): 120 μl of H_2_O, 15 μl of Primer 2&3 (10 μM) **(**Additional file 5: Table S4**)**, 150 μl of qPCR master mix (2**×**). A volume of 19 μl of the master mix was added into each PCR tube. One μl of enriched DNA was added to each PCR tube. PCR tubes were loaded into the real-time thermal cycler and run on the following PCR program: [95 °C for 5 min] × 1, [63 °C for 5 s, 72 °C for 10 s] × 39, melting curve at 50–90 °C for 5 s per degree. Reactions were analysed for the number of cycles required to achieve a saturated fluorescence signal. This number of cycles was then recorded and used as a guide for subsequent touchdown PCR amplification reactions to prepare sufficient DNA for Illumina sequencing.

A master mix was created to generate the sequencing library for 15 reactions as follows: (50 μl per reaction): 300 μl of H_2_O, 37.5 μl of 10 μM Primer 2 & 3 (Additional file 5: Table S4), 375 μl of Taq DNA polymerase master mix (2×). A volume of 47.5 μl of the master mix plus a volume of 2.5 μl of enriched DNA was added into each PCR tube. These tubes were moved to the thermocycler and the following PCR program used: [95 °C for 4 min] × 1, [95 °C for 30 s, 60 °C down 0.5 °C per cycle at 10 s, 72 °C for 4 min] × 10, [95 °C for 30 s, 45 °C for 30 s, 72 °C for 4 min] × 9, and stored at 4 °C. The PCR products were purified using the QIAquick PCR purification kit (Qiagen). The recovered DNA was quantified by Qubit dsDNA high sensitivity assay kit (Life Technologies). One hundred ng DNA from each enrichment reaction was pooled into one 1.5 ml-microcentrifuge tube and the total volume to was reduced to approximately 50 μl with a vacuum concentrator.

### Bind-n-seq: sequencing

The resulting pooled library was diluted to 2 nM with NaOH and 10 μL transferred into 990 μL Hybridization Buffer (HT1) (Illumina) to give a final concentration of 20 pM. A volume of 600 μl of the diluted library pool was spiked with 10% PhiX control v3 and placed on ice before loading into the Illumina MiSeq cartridge following the manufacturer’s instructions. The MiSeq Reagent Kit v3 (150 cycles) sequencing chemistry was utilised with run metrics of 150 cycles for each single end read using MiSeq Control Software 2.4.1.3 and Real-Time Analysis (RTA) 1.18.54.

### Data analysis

For data analysis, a new directory was created on the computer hard disk and used as working directory for the downstream analysis. The input sequencing file containing high quality sequences was placed into this directory (Note: that the input dataset should be in a compressed fastq.gz format). Other required files were downloaded from website:

https://anshiqi19840918.wixsite.com/ngsfilelinks/others and files saved to the same location as the sequencing file: background.txt (random 21mers that acts as the default background for a MERMADE run), Bind-n-seq 13-barcodes.csv (a comma-separated list of the possible 3 long bar-codes), which can be edited in excel to add meaningful names for specific libraries against the barcodes.

### Installation of MERMADE

The original MERMADE package was Dockerized, which can be run on diverse operation systems, including Windows. More information can be found at https://anshiqi19840918.wixsite.com/ngsfilelinks/others (for commands for running on macOS system please see Additional file 9: First, the latest version of the Docker Desktop for Windows was downloaded and installed following the instructions in https://hub.docker.com/editions/community/docker-ce-desktop-windows. In the terminal window switch directory with command *cd directoryname*. To pull and install the Dockerized MERMADE image by using following commands in a terminal window:

*docker pull pfcarrier/docker_mermade*

Then following commands were used for development of the container:

*docker run -v “directory path of the container”:/work -it pfcarrier/docker_mermade bash*

The prompt in the terminal window should change to: */work#*, which indicates the software has been installed successfully.

### Sequencing data analysis using MERMADE

In the working directory, MEMADE could be run with the command

*rm -rf databasename.db wdir;run_mermade.pl -o databasename.db -d wdir -b background.txt -v TGATCGGAAG sequencing.fastq.gz barcode.csv*

where ***databasename*** is the name of the database file; ***sequencing.fastq.gz*** is the name of the sequence file; ***barcode.csv*** is the name of the edited barcode.csv file with user library names (Note there are other optional parameters that can be further optimized by the user, but in general running the application with default setting is recommended).

An analysis report was generated by using reporter.pl script. The reporter.pl script. Was executable with command:

*reporter.pl < databasename.db > <# of motifs > <output dir > <barcodes>*

### Filtering and processing the results from MERMADE

Results from the MERMADE were processed by filtering low complexity patterns and those seed sequences with an enrichment below 2.5-fold over background and foreground reads less than 500. We applied an R script to select the final list of sequences that were submitted to the Regulatory Sequence Analysis Tools prokaryotes (RSAT). This script used the “.html” output generated by MERMADE and then identified 1) all the unique motifs; 2) shorter unique motifs that might be contained in longer ones; and 3) longer unique motifs (Please note that there are other software/applications available to search given motifs). RStudio can be downloaded and installed from: https://www.rstudio.com/ and ExtractMotifs zip file can be downloaded from https://anshiqi19840918.wixsite.com/ngsfilelinks/others. These files were unzipped and saved to the computer hard disk. A .txt file containing barcodes of interest was used (Please note the format of the file should be one barcode per line). RStudio was installed and packages loaded with the commands:

*install.packages(“plyr”)*

*library(“plyr”)*

*install.packages(“dplyr”)*

*library(“dplyr”)*

*install.packages(“stringi”)*

*library(“stringi”)*

*install.packages(“htmltab”)*

*library(“htmltab”)*

*install.packages(“stringr”)*

*library(“stringr”)*

*install.packages(“devtools”)*

*library(“devtools”)*

*source(“* *https://bioconductor.org/biocLite.R**“)*

*biocLite(“Biostrings”)*

*source(“* *https://bioconductor.org/biocLite.R**“)*

*biocLite(“DECIPHER”)*

*Install and run ExtractMotifs package with commands:*

*install.packages(“PathTo/ExtractMotifs_0.1.0.tar.gz”,repos = NULL, type = “source”)*

*library(“ExtractMotifs”)*

*x < −ExtractMotifs(“path_to_html_file”,Ratio_Threshold,Foreground,"path_to_Barcode_List”)*

The output from this command was three “.csv” files that were saved into the current R working directory and one HTML file that automatically open when the analysis was completed (Please note it was important to check the current active directory using the command *getwd().* The list named *BC_selected_Longest_Seqs.csv* was used for genome-scale DNA pattern searching using Regulatory Sequence Analysis Tools (RSAT) Prokaryotes. RSAT Prokaryotes genome-scale DNA-pattern search is available at: http://embnet.ccg.unam.mx/rsat/genome-scale-dna-pattern_form.cgi. In this case, the selected organism of interest to identify pattern(s) as Query pattern(s) to perform the search was *X. campestris pv. campestris* sequenced strain 8004 (Please note the parameters at RAST-genome-scale DNA-pattern can be optimised for more specific searches if required. For example, the search region can be narrowed down within 200 bp upstream of annotated ORFs and also the researcher can disable the option of allow overlap with upstream ORF).

## Supplementary information

**Additional file 1: Figure S1.** SDS/PAGE gel image shows a single band of the His6-MBP tag of the expected size of 81 kDa purified by affinity and size exclusive chromatography.

**Additional file 2: Table S1.** RAST-genome-scale DNA-pattern search result.

**Additional file 3: Table S2.** Fragment used for EMSA experiment.

**Additional file 4: Table S3.** Barcode assignment for Bind-n-seq.

**Additional file 5: Table S4.** Primers used in Bind-n-seq procedure.

**Additional file 6: Table S5.** Composition for KCl salt solutions.

**Additional file 7: Table S6.** Binding reaction conditions.

**Additional file 8: Table S7.** Composition for Bin-n-seq wash buffer.

**Additional file 9.** Analysis Bind-n-seq data using macOS operating system.

## Data Availability

Bind-n-seq sequence dataset generated and analyzed during the current study has been deposited in Mendeley Data and are accessible through DOI: 10.17632/vjb2dd6fzn.1

## Abbreviations

AEBSF: 4-(2-Aminoethyl) benzenesulfonyl fluoride hydrochloride
CAST: Cyclical Amplification and Selection of Targets
ChIP: Chromatin Immunoprecipitation
DBDs: DNA-binding domains
EMSA: Eelectrophoretic Mobility Shift Assays
ORFs: Open reading frames
PBM: Protein-Binding Microarray
qRT-PCR: Quantitative Reverse Transcription PCR
REC: CheY-homologous receiver
RSAT: Regulatory Sequence Analysis Tools prokaryotes
RTA: Real-Time Analysis
RT-PCR: Real-time PCR
SAGE: Serial Analysis of Gene Expression
SELEX: Systematic Evolution of Ligands by Exponential Enrichment
*Xcc*: *Xanthomonas campestris* pv. *campestris*
YipR: YajQ interacting protein regulator
