# Supplementary figures and images for: An improved bind-n-seq strategy to determine protein-DNA interactions validated using the bacterial transcriptional regulator YipR
Source: BMC Microbiol. 2020 Jan 2;20:1. doi: 10.1186/s12866-019-1672-7 (PMC6941359; doi:10.1186/s12866-019-1672-7)

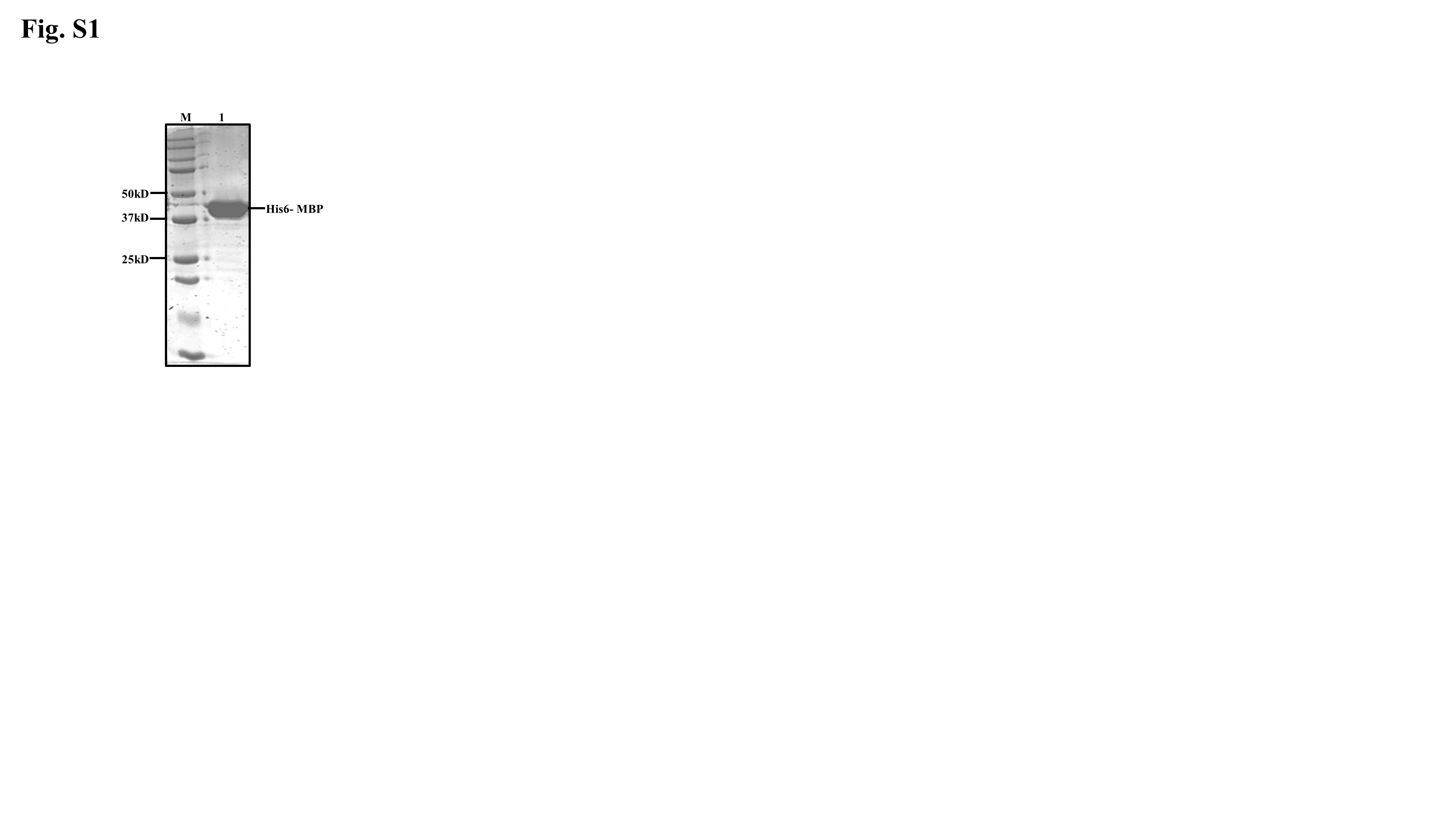

Supplement: Supplementary file 1 — Additional file 1: Figure S1. SDS/PAGE gel image shows a single band of the His6-MBP tag of the expected size of 81 kDa purified by affinity and size exclusive chromatography. [file 12866_2019_1672_MOESM1_ESM.jpg]
