# Supplementary material for: An improved bind-n-seq strategy to determine protein-DNA interactions validated using the bacterial transcriptional regulator YipR
Source: BMC Microbiol. 2020 Jan 2;20:1. doi: 10.1186/s12866-019-1672-7 (PMC6941359; doi:10.1186/s12866-019-1672-7)
Supplement: Supplementary file 2 — Additional file 2: Table S1. RAST-genome-scale DNA-pattern search result. [file 12866_2019_1672_MOESM2_ESM.docx]

**Supplementary Table S1. RAST-genome-scale DNA-pattern search result**

| SeqID | Strand | Pattern | Start | End | matching_seq | Score |
| --- | --- | --- | --- | --- | --- | --- |
| XC_RS00005 | R | CCCGTCCC | -186 | -179 | CCCGGCCC | 0.88 |
| XC_RS00015 | D | CCCCGTCC | -41 | -34 | CCCCGTCC | 1 |
| XC_RS00015 | D | CCCGTCCC | -40 | -33 | CCCGTCCG | 0.88 |
| XC_RS00020 | D | CCCCGTCC | -39 | -32 | CGCCGTCC | 0.88 |
| XC_RS00020 | D | CCCCTTCTCC | -67 | -58 | CCCCTTTTCC | 0.9 |
| XC_RS00025 | R | AGGCGGAGG | -60 | -52 | AGGCGGCGG | 0.89 |
| XC_RS00080 | D | CTCGCCGTC | -116 | -108 | CTCGCCGTT | 0.89 |
| XC_RS00090 | D | CCCCGTCC | -197 | -190 | CACCGTCC | 0.88 |
| XC_RS00095 | R | CCCCGTCC | -30 | -23 | CACCGTCC | 0.88 |
| XC_RS00100 | R | AGGCGGAGG | -20 | -12 | AGGCGCAGG | 0.89 |
| XC_RS00110 | R | CTCCCGTC | -63 | -56 | CTGCCGTC | 0.88 |
| XC_RS00115 | D | CCCGTCCC | -43 | -36 | CCCGACCC | 0.88 |
| XC_RS00115 | D | CTCCCGTC | -163 | -156 | CTCCCGCC | 0.88 |
| XC_RS00120 | R | CCCGTCCC | -135 | -128 | CCCGACCC | 0.88 |
| XC_RS00120 | R | CTCCCGTC | -15 | -8 | CTCCCGCC | 0.88 |
| XC_RS00125 | R | AGGCGGAGG | -50 | -42 | AGGCGGTGG | 0.89 |
| XC_RS00125 | D | CCCCCGCCTC | -24 | -15 | CCCCCGCCGC | 0.9 |
| XC_RS21985 | D | CTCCCGTC | -114 | -107 | CTCCCGCC | 0.88 |
| XC_RS00145 | R | AGGCGGAGG | -46 | -38 | AGGGGGAGG | 0.89 |
| XC_RS00150 | D | CCCGTCCC | -21 | -14 | TCCGTCCC | 0.88 |
| XC_RS21990 | D | AGGCGGAGG | -49 | -41 | AAGCGGAGG | 0.89 |
| XC_RS00155 | D | CTCCCGTC | -15 | -8 | CTACCGTC | 0.88 |
| XC_RS00160 | D | CCCCGTCC | -73 | -66 | CCCCGCCC | 0.88 |
| XC_RS00165 | D | CCCCTCTC | -142 | -135 | CCGCTCTC | 0.88 |
| XC_RS00165 | D | CTCCCGTC | -183 | -176 | CTCCCATC | 0.88 |
| XC_RS00170 | R | CCCGTCCC | -71 | -64 | CCCGGCCC | 0.88 |
| XC_RS00175 | D | CCCGTCCC | -122 | -115 | CCCGGCCC | 0.88 |
| XC_RS00190 | D | CTCCCGTC | -143 | -136 | CTTCCGTC | 0.88 |
| XC_RS00195 | R | CCCCGTCC | -170 | -163 | CTCCGTCC | 0.88 |
| XC_RS00200 | R | CCCGTCCC | -89 | -82 | CGCGTCCC | 0.88 |
| XC_RS00200 | D | CTCCCGTC | -64 | -57 | CTGCCGTC | 0.88 |
| XC_RS00210 | D | CTCGCCGTC | -85 | -77 | CTCGCTGTC | 0.89 |
| XC_RS00215 | R | CTCGCCGTC | -58 | -50 | CTCGCTGTC | 0.89 |
| XC_RS00240 | D | CTCGCCGTC | -86 | -78 | CTCGCCATC | 0.89 |
| XC_RS00240 | R | CTCGCCGTC | -15 | -7 | CTCGCCGTC | 1 |
| XC_RS00250 | D | CCCGTCCC | -57 | -50 | CCCGGCCC | 0.88 |
| XC_RS00255 | R | CCCGTCCC | -155 | -148 | CCCGGCCC | 0.88 |
| XC_RS00270 | D | CCCCTCTC | -121 | -114 | CCCCTGTC | 0.88 |
| XC_RS00270 | D | CCCCTCTC | -108 | -101 | CCCCTCTT | 0.88 |
| XC_RS00270 | D | CCTCTCTCCC | -73 | -64 | CCTCGCTCCC | 0.9 |
| XC_RS00280 | D | CCCCCGCCTC | -27 | -18 | CCCCCACCTC | 0.9 |
| XC_RS00340 | R | CTCGCCGTC | -54 | -46 | CTCGCCCTC | 0.89 |
| XC_RS00375 | D | CTCCCGTC | -198 | -191 | CTCCCGCC | 0.88 |
| XC_RS00375 | R | CTCCCGTC | -144 | -137 | CGCCCGTC | 0.88 |
| XC_RS00380 | D | CTCCCGTC | -145 | -138 | CTCGCGTC | 0.88 |
| XC_RS00380 | D | CTCGCCGTC | -126 | -118 | CCCGCCGTC | 0.89 |
| XC_RS00445 | R | CCCCGTCC | -131 | -124 | CCCGGTCC | 0.88 |
| XC_RS00450 | R | CCCCTCTC | -14 | -7 | CCCCTCTG | 0.88 |
| XC_RS00450 | D | CCCGTCCC | -118 | -111 | CCCGTTCC | 0.88 |
| XC_RS00485 | D | CCCCGTCC | -135 | -128 | CACCGTCC | 0.88 |
| XC_RS00490 | D | CCCCGTCC | -186 | -179 | CCCCGTCG | 0.88 |
| XC_RS00490 | R | CTCGCCGTC | -195 | -187 | CTCGCAGTC | 0.89 |
| XC_RS00500 | D | CTCCCGTC | -131 | -124 | CTCCCGGC | 0.88 |
| XC_RS00500 | R | CTCCCGTC | -148 | -141 | CTCCCGGC | 0.88 |
| XC_RS00505 | D | CTCCCGTC | -25 | -18 | CTTCCGTC | 0.88 |
| XC_RS00510 | R | CTCCCGTC | -83 | -76 | CTTCCGTC | 0.88 |
| XC_RS00525 | R | CCCCGTCC | -163 | -156 | CCCCGTGC | 0.88 |
| XC_RS00550 | D | CCCCCGCCTC | -22 | -13 | CCCCCGCCGC | 0.9 |
| XC_RS00580 | D | CTCCCGTC | -24 | -17 | CTCCGGTC | 0.88 |
| XC_RS00590 | D | CCCCTCTC | -33 | -26 | CCCCTTTC | 0.88 |
| XC_RS00605 | D | CCCCTCTC | -78 | -71 | CGCCTCTC | 0.88 |
| XC_RS00605 | R | CCCCTCTC | -150 | -143 | CCCCACTC | 0.88 |
| XC_RS00605 | R | CCCGTCCC | -86 | -79 | CCGGTCCC | 0.88 |
| XC_RS00615 | R | CCCCTCTC | -13 | -6 | CCCCTCCC | 0.88 |
| XC_RS00615 | R | CCCGTCCC | -13 | -6 | CCCCTCCC | 0.88 |
| XC_RS00615 | R | CTCCCGTC | -11 | -4 | CTCCCCTC | 0.88 |
| XC_RS00635 | D | AGGCGGAGG | -63 | -55 | AGGCGGTGG | 0.89 |
| XC_RS00640 | R | CCCCTTCTCC | -191 | -182 | CACCTTCTCC | 0.9 |
| XC_RS22040 | D | CCCCGTCC | -22 | -15 | CCCCTTCC | 0.88 |
| XC_RS22040 | D | CCCGTCCC | -21 | -14 | CCCTTCCC | 0.88 |
| XC_RS00695 | D | CTCCCGTC | -197 | -190 | CTCCCGCC | 0.88 |
| XC_RS00695 | R | AGGCGGAGG | -106 | -98 | AGGCGGAGT | 0.89 |
| XC_RS00700 | R | CTCCCGTC | -120 | -113 | CTCCCGCC | 0.88 |
| XC_RS00740 | R | CCCCGTCC | -73 | -66 | CCCCATCC | 0.88 |
| XC_RS00755 | R | AGGCGGAGG | -115 | -107 | AGGCGGCGG | 0.89 |
| XC_RS00760 | R | CCCCTCTC | -77 | -70 | CCCCTCCC | 0.88 |
| XC_RS00760 | R | CCCGTCCC | -77 | -70 | CCCCTCCC | 0.88 |
| XC_RS00765 | D | CCCCTCTC | -137 | -130 | CCCCTCCC | 0.88 |
| XC_RS00765 | D | CCCGTCCC | -137 | -130 | CCCCTCCC | 0.88 |
| XC_RS00785 | D | CTCCCGTC | -78 | -71 | CTCCCGCC | 0.88 |
| XC_RS00785 | R | CTCCCGTC | -87 | -80 | CTCCCGCC | 0.88 |
| XC_RS22060 | D | CTCCCGTC | -133 | -126 | CTCCCCTC | 0.88 |
| XC_RS22060 | R | CCCCTTCTCC | -124 | -115 | CACCTTCTCC | 0.9 |
| XC_RS00795 | D | CCCCTCTC | -26 | -19 | CCCCTCGC | 0.88 |
| XC_RS00795 | R | CTCCCGTC | -141 | -134 | CTCCCCTC | 0.88 |
| XC_RS00795 | D | CCCCTTCTCC | -152 | -143 | CACCTTCTCC | 0.9 |
| XC_RS00800 | D | CTCGCCGTC | -72 | -64 | CCCGCCGTC | 0.89 |
| XC_RS00805 | R | CCCCTTCTCC | -130 | -121 | CACCTTCTCC | 0.9 |
| XC_RS00810 | R | CCCCTCTC | -95 | -88 | CCGCTCTC | 0.88 |
| XC_RS00830 | D | CCCCGTCC | -117 | -110 | CCCCGTCG | 0.88 |
| XC_RS00830 | D | CTCCCGTC | -118 | -111 | CCCCCGTC | 0.88 |
| XC_RS00835 | R | CCCGTCCC | -66 | -59 | CGCGTCCC | 0.88 |
| XC_RS00840 | R | CTCCCGTC | -16 | -9 | CTCCTGTC | 0.88 |
| XC_RS00860 | D | CTCCCGTC | -36 | -29 | CGCCCGTC | 0.88 |
| XC_RS00890 | D | CCCCGTCC | -135 | -128 | CCCCTTCC | 0.88 |
| XC_RS00890 | D | CCCCGTCC | -124 | -117 | CCCCATCC | 0.88 |
| XC_RS00890 | R | CCCCGTCC | -83 | -76 | GCCCGTCC | 0.88 |
| XC_RS00890 | D | CCCGTCCC | -134 | -127 | CCCTTCCC | 0.88 |
| XC_RS00890 | R | CCCGTCCC | -84 | -77 | CCCGTCCG | 0.88 |
| XC_RS00890 | D | CTCCCGTC | -131 | -124 | TTCCCGTC | 0.88 |
| XC_RS00890 | R | CTCCCGTC | -82 | -75 | CGCCCGTC | 0.88 |
| XC_RS00890 | D | AGGCGGAGG | -87 | -79 | AGGCGGACG | 0.89 |
| XC_RS00895 | D | CCCCGTCC | -86 | -79 | GCCCGTCC | 0.88 |
| XC_RS00895 | R | CCCCGTCC | -45 | -38 | CCCCATCC | 0.88 |
| XC_RS00895 | R | CCCCGTCC | -34 | -27 | CCCCTTCC | 0.88 |
| XC_RS00895 | D | CCCGTCCC | -85 | -78 | CCCGTCCG | 0.88 |
| XC_RS00895 | R | CCCGTCCC | -40 | -33 | CCCGTCCC | 1 |
| XC_RS00895 | D | CTCCCGTC | -87 | -80 | CGCCCGTC | 0.88 |
| XC_RS00895 | R | CTCCCGTC | -38 | -31 | TTCCCGTC | 0.88 |
| XC_RS00895 | R | AGGCGGAGG | -83 | -75 | AGGCGGACG | 0.89 |
| XC_RS00935 | D | CCCCGTCC | -24 | -17 | CCCCGTTC | 0.88 |
| XC_RS00935 | D | CCCGTCCC | -23 | -16 | CCCGTTCC | 0.88 |
| XC_RS00935 | D | CTCCCGTC | -77 | -70 | CTCCCGGC | 0.88 |
| XC_RS00950 | D | CTCGCCGTC | -109 | -101 | CTGGCCGTC | 0.89 |
| XC_RS00970 | R | CCCGTCCC | -195 | -188 | CTCGTCCC | 0.88 |
| XC_RS00970 | R | CTCCCGTC | -193 | -186 | CTCTCGTC | 0.88 |
| XC_RS00970 | D | AGGCGGAGG | -145 | -137 | AGGCGGATG | 0.89 |
| XC_RS00980 | D | CCCCGTCC | -195 | -188 | CCCCGGCC | 0.88 |
| XC_RS00980 | D | CCCCGTCC | -61 | -54 | CCCCGTCG | 0.88 |
| XC_RS00980 | D | CCCGTCCC | -194 | -187 | CCCGGCCC | 0.88 |
| XC_RS00980 | D | CCCGTCCC | -145 | -138 | CCCGCCCC | 0.88 |
| XC_RS00995 | D | CCCCGTCC | -13 | -6 | CCCCGTGC | 0.88 |
| XC_RS01000 | D | CCCCGTCC | -77 | -70 | CCCCGGCC | 0.88 |
| XC_RS01000 | D | CCCCGTCC | -41 | -34 | CCCCGGCC | 0.88 |
| XC_RS01000 | D | CCCGTCCC | -81 | -74 | CCCGCCCC | 0.88 |
| XC_RS01000 | D | CCCGTCCC | -60 | -53 | CCCGCCCC | 0.88 |
| XC_RS01000 | R | CTCGCCGTC | -21 | -13 | CTCGCCGCC | 0.89 |
| XC_RS01030 | R | AGGCGGAGG | -34 | -26 | AGGCGCAGG | 0.89 |
| XC_RS01035 | D | CCCGTCCC | -15 | -8 | CCCGACCC | 0.88 |
| XC_RS01040 | R | CCCGTCCC | -177 | -170 | CCCGACCC | 0.88 |
| XC_RS01045 | D | CTCGCCGTC | -9 | -1 | TTCGCCGTC | 0.89 |
| XC_RS01055 | R | AGGCGGAGG | -48 | -40 | AGGCGGAAG | 0.89 |
| XC_RS01080 | D | CCCCTCTC | -19 | -12 | CTCCTCTC | 0.88 |
| XC_RS01095 | D | CCCCGTCC | -132 | -125 | CCCCGTGC | 0.88 |
| XC_RS01100 | D | CCCCTCTC | -77 | -70 | CGCCTCTC | 0.88 |
| XC_RS01100 | R | CTCCCGTC | -111 | -104 | CTCCCGCC | 0.88 |
| XC_RS01100 | D | AGGCGGAGG | -194 | -186 | AGGCGGCGG | 0.89 |
| XC_RS01105 | D | CCCCTCTC | -30 | -23 | CCCCTCGC | 0.88 |
| XC_RS01145 | D | AGGCGGAGG | -68 | -60 | AGGCGCAGG | 0.89 |
| XC_RS01160 | D | CCCGTCCC | -161 | -154 | ACCGTCCC | 0.88 |
| XC_RS01160 | R | CCCGTCCC | -126 | -119 | CCCGGCCC | 0.88 |
| XC_RS01160 | D | CTCCCGTC | -163 | -156 | CTACCGTC | 0.88 |
| XC_RS01160 | R | CTCCCGTC | -135 | -128 | CGCCCGTC | 0.88 |
| XC_RS01160 | R | CTCCCGTC | -13 | -6 | CTCCTGTC | 0.88 |
| XC_RS01165 | D | CCCGTCCC | -54 | -47 | CCCGGCCC | 0.88 |
| XC_RS01165 | R | CCCGTCCC | -19 | -12 | ACCGTCCC | 0.88 |
| XC_RS01165 | D | CTCCCGTC | -167 | -160 | CTCCTGTC | 0.88 |
| XC_RS01165 | D | CTCCCGTC | -45 | -38 | CGCCCGTC | 0.88 |
| XC_RS01165 | R | CTCCCGTC | -17 | -10 | CTACCGTC | 0.88 |
| XC_RS01180 | R | CCCCGTCC | -78 | -71 | GCCCGTCC | 0.88 |
| XC_RS01180 | D | CCCGTCCC | -27 | -20 | CCGGTCCC | 0.88 |
| XC_RS01180 | R | CCCGTCCC | -79 | -72 | CCCGTCCC | 1 |
| XC_RS01180 | R | CTCCCGTC | -77 | -70 | CGCCCGTC | 0.88 |
| XC_RS01185 | D | CCCCGTCC | -24 | -17 | GCCCGTCC | 0.88 |
| XC_RS01185 | D | CCCGTCCC | -23 | -16 | CCCGTCCC | 1 |
| XC_RS01185 | R | CCCGTCCC | -75 | -68 | CCGGTCCC | 0.88 |
| XC_RS01185 | D | CTCCCGTC | -25 | -18 | CGCCCGTC | 0.88 |
| XC_RS01225 | D | CTCGCCGTC | -22 | -14 | CTCGCCTTC | 0.89 |
| XC_RS01235 | R | AGGCGGAGG | -88 | -80 | AGGCGCAGG | 0.89 |
| XC_RS01240 | D | AGGCGGAGG | -41 | -33 | AGGCGCAGG | 0.89 |
| XC_RS01245 | D | CCCCGTCC | -27 | -20 | CCCCATCC | 0.88 |
| XC_RS01245 | D | CCCGTCCC | -26 | -19 | CCCATCCC | 0.88 |
| XC_RS01250 | R | CCCCTCTC | -160 | -153 | CCCCTCCC | 0.88 |
| XC_RS01250 | R | CCCCGTCC | -159 | -152 | CCCCCTCC | 0.88 |
| XC_RS01250 | R | CCCGTCCC | -160 | -153 | CCCCTCCC | 0.88 |
| XC_RS01290 | D | CCCGTCCC | -29 | -22 | CACGTCCC | 0.88 |
| XC_RS01295 | R | CCCGTCCC | -107 | -100 | CACGTCCC | 0.88 |
| XC_RS01320 | D | CCCCGTCC | -13 | -6 | CACCGTCC | 0.88 |
| XC_RS01390 | D | CCCGTCCC | -28 | -21 | CCCGACCC | 0.88 |
| XC_RS22080 | D | CCCCGTCC | -185 | -178 | CCCCGACC | 0.88 |
| XC_RS22080 | D | CCCGTCCC | -184 | -177 | CCCGACCC | 0.88 |
| XC_RS01395 | R | CCCCTCTC | -184 | -177 | CCCCTGTC | 0.88 |
| XC_RS01425 | D | CTCGCCGTC | -130 | -122 | ATCGCCGTC | 0.89 |
| XC_RS01435 | R | CCCCGTCC | -167 | -160 | CCCCGTCC | 1 |
| XC_RS01435 | R | CCCCGTCC | -76 | -69 | GCCCGTCC | 0.88 |
| XC_RS01435 | D | CCCGTCCC | -16 | -9 | CCCGCCCC | 0.88 |
| XC_RS01435 | R | CCCGTCCC | -168 | -161 | CCCGTCCC | 1 |
| XC_RS01435 | R | CCCGTCCC | -77 | -70 | CCCGTCCG | 0.88 |
| XC_RS01435 | R | CTCGCCGTC | -166 | -158 | CTCCCCGTC | 0.89 |
| XC_RS01435 | R | CCCCCGCCTC | -176 | -167 | CCCCCGCTTC | 0.9 |
| XC_RS01440 | D | CCCCGTCC | -137 | -130 | CCCCGTCC | 1 |
| XC_RS01440 | D | CCCGTCCC | -136 | -129 | CCCGTCCC | 1 |
| XC_RS01440 | D | CTCGCCGTC | -139 | -131 | CTCCCCGTC | 0.89 |
| XC_RS01440 | D | CCCCCGCCTC | -130 | -121 | CCCCCGCTTC | 0.9 |
| XC_RS01480 | D | CCCCTCTC | -98 | -91 | CCCCTCCC | 0.88 |
| XC_RS01480 | D | CCCCGTCC | -28 | -21 | CCCTGTCC | 0.88 |
| XC_RS01480 | D | CCCGTCCC | -176 | -169 | CCCGTCGC | 0.88 |
| XC_RS01480 | D | CCCGTCCC | -98 | -91 | CCCCTCCC | 0.88 |
| XC_RS01480 | D | CCCGTCCC | -27 | -20 | CCTGTCCC | 0.88 |
| XC_RS01480 | D | CTCCCGTC | -95 | -88 | CTCCCGCC | 0.88 |
| XC_RS01485 | R | CCCGTCCC | -144 | -137 | CCCGTCGC | 0.88 |
| XC_RS01490 | R | CCCCGTCC | -16 | -9 | CCGCGTCC | 0.88 |
| XC_RS01490 | R | CCCGTCCC | -17 | -10 | CGCGTCCC | 0.88 |
| XC_RS23605 | D | AGGCGGAGG | -150 | -142 | GGGCGGAGG | 0.89 |
| XC_RS23605 | D | CTCGCCGTC | -183 | -175 | CTGGCCGTC | 0.89 |
| XC_RS01500 | D | CCCGTCCC | -27 | -20 | CCCGTTCC | 0.88 |
| XC_RS01500 | D | CTCCCGTC | -29 | -22 | CTCCCGTT | 0.88 |
| XC_RS01500 | D | CTCGCCGTC | -67 | -59 | CACGCCGTC | 0.89 |
| XC_RS01520 | D | CCCCTCTC | -74 | -67 | CCCCTCTC | 1 |
| XC_RS01520 | R | CCCCTCTC | -59 | -52 | CCCCTCTC | 1 |
| XC_RS01520 | D | CTCCCGTC | -69 | -62 | CTCCCGGC | 0.88 |
| XC_RS01520 | R | CTCCCGTC | -64 | -57 | CTCCCGCC | 0.88 |
| XC_RS01520 | D | CCTCTCTCCC | -74 | -65 | CCCCTCTCCC | 0.9 |
| XC_RS01520 | R | CCTCTCTCCC | -61 | -52 | CCCCTCTCCC | 0.9 |
| XC_RS01525 | D | CCCCGTCC | -69 | -62 | CCACGTCC | 0.88 |
| XC_RS01545 | R | CCCCGTCC | -126 | -119 | CCCCGCCC | 0.88 |
| XC_RS01545 | R | CCCGTCCC | -127 | -120 | CCCGCCCC | 0.88 |
| XC_RS01545 | D | CTCCCGTC | -27 | -20 | CTCCCGCC | 0.88 |
| XC_RS01550 | D | CCCCGTCC | -9 | -2 | CCCCGCCC | 0.88 |
| XC_RS01550 | D | CCCGTCCC | -8 | -1 | CCCGCCCC | 0.88 |
| XC_RS01550 | R | CTCCCGTC | -108 | -101 | CTCCCGCC | 0.88 |
| XC_RS01555 | R | CCCCTCTC | -71 | -64 | CCCGTCTC | 0.88 |
| XC_RS01555 | R | CCCGTCCC | -71 | -64 | CCCGTCTC | 0.88 |
| XC_RS01555 | R | CTCCCGTC | -76 | -69 | CTCCCATC | 0.88 |
| XC_RS01555 | D | CCCCTTCTCC | -87 | -78 | CACCTTCTCC | 0.9 |
| XC_RS01590 | R | CTCCCGTC | -15 | -8 | CTCCGGTC | 0.88 |
| XC_RS01610 | D | CCCCGTCC | -176 | -169 | CCGCGTCC | 0.88 |
| XC_RS01625 | D | CCCCTCTC | -192 | -185 | GCCCTCTC | 0.88 |
| XC_RS01630 | R | CCCCGTCC | -17 | -10 | CGCCGTCC | 0.88 |
| XC_RS01705 | D | CTCCCGTC | -8 | -1 | CTCCCGTC | 1 |
| XC_RS01725 | D | CCCCTCTC | -148 | -141 | CCACTCTC | 0.88 |
| XC_RS22105 | R | CCCCTCTC | -21 | -14 | CCACTCTC | 0.88 |
| XC_RS22110 | D | CCCCTCTC | -48 | -41 | CGCCTCTC | 0.88 |
| XC_RS22110 | R | CCCCTCTC | -97 | -90 | CCCCTTTC | 0.88 |
| XC_RS22110 | D | CTCCCGTC | -171 | -164 | CTCCCGTT | 0.88 |
| XC_RS22110 | D | AGGCGGAGG | -183 | -175 | AGGCGGAAG | 0.89 |
| XC_RS01785 | R | CCCCTCTC | -21 | -14 | CCACTCTC | 0.88 |
| XC_RS01790 | D | CCCCGTCC | -108 | -101 | CCCCTTCC | 0.88 |
| XC_RS01790 | D | CCCGTCCC | -107 | -100 | CCCTTCCC | 0.88 |
| XC_RS01795 | R | CCCCGTCC | -129 | -122 | CCCCTTCC | 0.88 |
| XC_RS01795 | R | CCCGTCCC | -130 | -123 | CCCTTCCC | 0.88 |
| XC_RS22135 | R | CCCCGTCC | -109 | -102 | CCCCGTCA | 0.88 |
| XC_RS22135 | R | CTCCCGTC | -108 | -101 | CCCCCGTC | 0.88 |
| XC_RS01825 | D | CCCCTCTC | -21 | -14 | CCCCCCTC | 0.88 |
| XC_RS01830 | D | CCCGTCCC | -96 | -89 | CCCGGCCC | 0.88 |
| XC_RS01835 | D | CTCCCGTC | -79 | -72 | CTCACGTC | 0.88 |
| XC_RS01880 | R | CCCCGTCC | -14 | -7 | CTCCGTCC | 0.88 |
| XC_RS01880 | R | CCCGTCCC | -15 | -8 | TCCGTCCC | 0.88 |
| XC_RS01890 | D | AGGCGGAGG | -41 | -33 | AGGCGCAGG | 0.89 |
| XC_RS01940 | D | CCCCGTCC | -133 | -126 | CCGCGTCC | 0.88 |
| XC_RS01940 | D | CCCGTCCC | -27 | -20 | CCCGGCCC | 0.88 |
| XC_RS01945 | D | CCCCGTCC | -84 | -77 | CCCCGTAC | 0.88 |
| XC_RS01945 | R | CCCCGTCC | -80 | -73 | CCCCGTAC | 0.88 |
| XC_RS01950 | D | CCCCGTCC | -79 | -72 | CCCCGTAC | 0.88 |
| XC_RS01950 | R | CCCCGTCC | -75 | -68 | CCCCGTAC | 0.88 |
| XC_RS01955 | D | CTCGCCGTC | -32 | -24 | CTCGGCGTC | 0.89 |
| XC_RS01990 | D | CCCCGTCC | -24 | -17 | CCCCGCCC | 0.88 |
| XC_RS01990 | D | CCCGTCCC | -82 | -75 | CCCATCCC | 0.88 |
| XC_RS01990 | D | CCCGTCCC | -23 | -16 | CCCGCCCC | 0.88 |
| XC_RS01990 | D | CCCCCGCCTC | -25 | -16 | CCCCCGCCCC | 0.9 |
| XC_RS02000 | D | CCCCTCTC | -154 | -147 | CACCTCTC | 0.88 |
| XC_RS02100 | D | CCCGTCCC | -126 | -119 | CCAGTCCC | 0.88 |
| XC_RS02100 | D | CTCGCCGTC | -107 | -99 | CTCGCCGAC | 0.89 |
| XC_RS02105 | R | CCCCGTCC | -92 | -85 | CCCCGGCC | 0.88 |
| XC_RS02105 | D | CCCGTCCC | -188 | -181 | CCCGACCC | 0.88 |
| XC_RS23615 | D | CTCGCCGTC | -141 | -133 | CTCACCGTC | 0.89 |
| XC_RS02110 | R | CTCGCCGTC | -56 | -48 | CTCGCCGTG | 0.89 |
| XC_RS02110 | D | CCTCTCTCCC | -25 | -16 | CCTCTTTCCC | 0.9 |
| XC_RS02115 | D | CCCCGTCC | -80 | -73 | CCCCGTTC | 0.88 |
| XC_RS02115 | R | CCCGTCCC | -69 | -62 | CCCGGCCC | 0.88 |
| XC_RS02120 | R | CCCCGTCC | -168 | -161 | CCCCGTTC | 0.88 |
| XC_RS02120 | D | CCCGTCCC | -179 | -172 | CCCGGCCC | 0.88 |
| XC_RS02190 | D | CCCCTCTC | -23 | -16 | CCCCTGTC | 0.88 |
| XC_RS22155 | D | CCCGTCCC | -165 | -158 | CCAGTCCC | 0.88 |
| XC_RS02245 | D | CTCGCCGTC | -63 | -55 | CTCGCCGGC | 0.89 |
| XC_RS02250 | D | CCCCGTCC | -60 | -53 | CGCCGTCC | 0.88 |
| XC_RS02250 | D | CTCGCCGTC | -62 | -54 | TTCGCCGTC | 0.89 |
| XC_RS02255 | R | CCCCGTCC | -189 | -182 | CCCCGTCT | 0.88 |
| XC_RS02255 | R | CCCCGTCC | -58 | -51 | CCACGTCC | 0.88 |
| XC_RS02255 | R | CCCGTCCC | -59 | -52 | CACGTCCC | 0.88 |
| XC_RS02255 | R | CTCGCCGTC | -188 | -180 | CTCCCCGTC | 0.89 |
| XC_RS02265 | R | CCCCTCTC | -172 | -165 | CCCTTCTC | 0.88 |
| XC_RS02265 | D | CTCCCGTC | -127 | -120 | CACCCGTC | 0.88 |
| XC_RS02265 | D | CCCCTTCTCC | -188 | -179 | CACCTTCTCC | 0.9 |
| XC_RS02265 | R | CCCCTTCTCC | -173 | -164 | CCCCTTCTCC | 1 |
| XC_RS02285 | D | CCCCTCTC | -47 | -40 | CCCCTCTC | 1 |
| XC_RS02285 | R | CCCCTCTC | -32 | -25 | CCCCTCTC | 1 |
| XC_RS02285 | D | CCTCTCTCCC | -47 | -38 | CCCCTCTCCC | 0.9 |
| XC_RS02285 | R | CCTCTCTCCC | -34 | -25 | CCCCTCTCCC | 0.9 |
| XC_RS02335 | R | CTCCCGTC | -85 | -78 | CGCCCGTC | 0.88 |
| XC_RS02340 | D | CTCCCGTC | -54 | -47 | CGCCCGTC | 0.88 |
| XC_RS02365 | D | CCCGTCCC | -46 | -39 | CCCATCCC | 0.88 |
| XC_RS02395 | D | CTCCCGTC | -144 | -137 | CGCCCGTC | 0.88 |
| XC_RS02395 | R | AGGCGGAGG | -148 | -140 | GGGCGGAGG | 0.89 |
| XC_RS02465 | R | CCCCTCTC | -112 | -105 | CCCCTTTC | 0.88 |
| XC_RS02465 | R | CTCCCGTC | -117 | -110 | TTCCCGTC | 0.88 |
| XC_RS02470 | D | CCCCTCTC | -33 | -26 | CCCCTTTC | 0.88 |
| XC_RS02470 | D | CTCCCGTC | -28 | -21 | TTCCCGTC | 0.88 |
| XC_RS02475 | R | CCCGTCCC | -107 | -100 | GCCGTCCC | 0.88 |
| XC_RS02480 | R | CCCCGTCC | -22 | -15 | CCCCGGCC | 0.88 |
| XC_RS02485 | D | CCCCGTCC | -169 | -162 | CCCCGGCC | 0.88 |
| XC_RS02530 | R | CCCCGTCC | -114 | -107 | GCCCGTCC | 0.88 |
| XC_RS02530 | R | CCCGTCCC | -115 | -108 | CCCGTCCG | 0.88 |
| XC_RS02535 | D | CCCCGTCC | -20 | -13 | GCCCGTCC | 0.88 |
| XC_RS02535 | D | CCCGTCCC | -19 | -12 | CCCGTCCG | 0.88 |
| XC_RS02565 | D | AGGCGGAGG | -170 | -162 | AGGCGCAGG | 0.89 |
| XC_RS02575 | R | CCCCGTCC | -80 | -73 | CCCCATCC | 0.88 |
| XC_RS02580 | D | CCCCGTCC | -28 | -21 | CCCCATCC | 0.88 |
| XC_RS02605 | D | CCTCTCTCCC | -24 | -15 | CCTCTTTCCC | 0.9 |
| XC_RS02640 | R | CTCCCGTC | -113 | -106 | CTGCCGTC | 0.88 |
| XC_RS02640 | R | CTCGCCGTC | -90 | -82 | CCCGCCGTC | 0.89 |
| XC_RS02645 | D | CCCCTCTC | -47 | -40 | CCCCTCGC | 0.88 |
| XC_RS02645 | D | CCCGTCCC | -33 | -26 | CGCGTCCC | 0.88 |
| XC_RS02645 | D | CTCGCCGTC | -44 | -36 | CTCGCCGGC | 0.89 |
| XC_RS22200 | D | CCCGTCCC | -130 | -123 | CACGTCCC | 0.88 |
| XC_RS02655 | D | CCCGTCCC | -29 | -22 | CCCGGCCC | 0.88 |
| XC_RS02655 | D | CTCCCGTC | -31 | -24 | CTCCCGGC | 0.88 |
| XC_RS02660 | R | CCCGTCCC | -93 | -86 | CCCGTTCC | 0.88 |
| XC_RS02660 | R | CTCCCGTC | -86 | -79 | CTCCCGGC | 0.88 |
| XC_RS22205 | R | CTCGCCGTC | -120 | -112 | CGCGCCGTC | 0.89 |
| XC_RS02670 | D | CTCGCCGTC | -46 | -38 | CGCGCCGTC | 0.89 |
| XC_RS02705 | D | CCCCTCTC | -136 | -129 | CCCCGCTC | 0.88 |
| XC_RS02705 | D | CCCCGTCC | -152 | -145 | CCCCGGCC | 0.88 |
| XC_RS02705 | D | CCCGTCCC | -151 | -144 | CCCGGCCC | 0.88 |
| XC_RS02720 | D | CTCGCCGTC | -83 | -75 | CTCGCCATC | 0.89 |
| XC_RS02725 | R | CTCCCGTC | -38 | -31 | CTCTCGTC | 0.88 |
| XC_RS02760 | R | CCCCTCTC | -89 | -82 | CCCCTGTC | 0.88 |
| XC_RS02760 | R | CCCCGTCC | -105 | -98 | CCCCGACC | 0.88 |
| XC_RS02760 | R | CCCCGTCC | -90 | -83 | CCCTGTCC | 0.88 |
| XC_RS02780 | D | AGGCGGAGG | -135 | -127 | TGGCGGAGG | 0.89 |
| XC_RS02790 | D | CCCCGTCC | -59 | -52 | CCCCGCCC | 0.88 |
| XC_RS02790 | D | CCCCGTCC | -22 | -15 | CCCCGTCA | 0.88 |
| XC_RS02790 | D | CCCGTCCC | -21 | -14 | CCCGTCAC | 0.88 |
| XC_RS02790 | D | CTCCCGTC | -23 | -16 | CCCCCGTC | 0.88 |
| XC_RS02790 | R | AGGCGGAGG | -99 | -91 | AGGCGGCGG | 0.89 |
| XC_RS02790 | R | CTCGCCGTC | -122 | -114 | CTTGCCGTC | 0.89 |
| XC_RS02795 | R | CCCCGTCC | -123 | -116 | CCCCGTCA | 0.88 |
| XC_RS02795 | R | CCCCGTCC | -86 | -79 | CCCCGCCC | 0.88 |
| XC_RS02795 | R | CCCGTCCC | -124 | -117 | CCCGTCAC | 0.88 |
| XC_RS02795 | R | CTCCCGTC | -122 | -115 | CCCCCGTC | 0.88 |
| XC_RS02795 | D | AGGCGGAGG | -47 | -39 | AGGCGGCGG | 0.89 |
| XC_RS02795 | D | CTCGCCGTC | -24 | -16 | CTTGCCGTC | 0.89 |
| XC_RS02800 | D | CCCGTCCC | -171 | -164 | CCCGTCGC | 0.88 |
| XC_RS02800 | D | CTCGCCGTC | -9 | -1 | CTTGCCGTC | 0.89 |
| XC_RS22220 | R | CCCCCGCCTC | -30 | -21 | CACCCGCCTC | 0.9 |
| XC_RS02825 | D | CCCCTCTC | -9 | -2 | CACCTCTC | 0.88 |
| XC_RS02825 | R | CCCCTCTC | -139 | -132 | CCCATCTC | 0.88 |
| XC_RS22225 | R | CTCCCGTC | -169 | -162 | CTGCCGTC | 0.88 |
| XC_RS02855 | D | CCCCGTCC | -28 | -21 | CGCCGTCC | 0.88 |
| XC_RS02880 | D | CCCCTCTC | -52 | -45 | CCCCTGTC | 0.88 |
| XC_RS02880 | D | CCCCTCTC | -19 | -12 | CCCATCTC | 0.88 |
| XC_RS02880 | D | CCCCGTCC | -65 | -58 | CCTCGTCC | 0.88 |
| XC_RS02880 | D | CCCCGTCC | -51 | -44 | CCCTGTCC | 0.88 |
| XC_RS02880 | D | CCCGTCCC | -50 | -43 | CCTGTCCC | 0.88 |
| XC_RS02885 | D | CCCGTCCC | -49 | -42 | CCCATCCC | 0.88 |
| XC_RS02885 | D | CCCCCGCCTC | -43 | -34 | CCCCCGCCAC | 0.9 |
| XC_RS02940 | R | CCCGTCCC | -68 | -61 | CACGTCCC | 0.88 |
| XC_RS02955 | R | CTCCCGTC | -14 | -7 | CTCACGTC | 0.88 |
| XC_RS22235 | D | CTCCCGTC | -82 | -75 | CTCACGTC | 0.88 |
| XC_RS02975 | D | CCCCGTCC | -121 | -114 | CACCGTCC | 0.88 |
| XC_RS02975 | D | CCCGTCCC | -120 | -113 | ACCGTCCC | 0.88 |
| XC_RS02990 | R | CCCGTCCC | -71 | -64 | CCCGTCAC | 0.88 |
| XC_RS02995 | R | CTCCCGTC | -112 | -105 | CGCCCGTC | 0.88 |
| XC_RS03040 | D | CCCCTCTC | -133 | -126 | ACCCTCTC | 0.88 |
| XC_RS03040 | D | CCCGTCCC | -25 | -18 | CCCGACCC | 0.88 |
| XC_RS03070 | D | CCCGTCCC | -51 | -44 | GCCGTCCC | 0.88 |
| XC_RS03080 | D | CTCGCCGTC | -69 | -61 | GTCGCCGTC | 0.89 |
| XC_RS03090 | D | CCCCGTCC | -83 | -76 | CCCCGCCC | 0.88 |
| XC_RS03105 | D | CTCCCGTC | -27 | -20 | CTCCCGCC | 0.88 |
| XC_RS03110 | R | CTCCCGTC | -26 | -19 | CTCCAGTC | 0.88 |
| XC_RS03115 | D | CTCCCGTC | -143 | -136 | CTCCAGTC | 0.88 |
| XC_RS03130 | R | CTCCCGTC | -25 | -18 | CTCCCGGC | 0.88 |
| XC_RS03135 | D | CTCCCGTC | -35 | -28 | CTCCCGGC | 0.88 |
| XC_RS03140 | D | CCCGTCCC | -29 | -22 | CCGGTCCC | 0.88 |
| XC_RS03160 | R | CCCGTCCC | -199 | -192 | CCCGTTCC | 0.88 |
| XC_RS03165 | D | CCCCTCTC | -21 | -14 | CCCATCTC | 0.88 |
| XC_RS22250 | R | CCCCTCTC | -106 | -99 | CCCATCTC | 0.88 |
| XC_RS03170 | R | CTCCCGTC | -185 | -178 | CTCCCGGC | 0.88 |
| XC_RS03190 | D | CCCCGTCC | -22 | -15 | CCCCGCCC | 0.88 |
| XC_RS03190 | D | CCCGTCCC | -86 | -79 | CCGGTCCC | 0.88 |
| XC_RS03190 | R | CCCGTCCC | -183 | -176 | CCCGTGCC | 0.88 |
| XC_RS03205 | D | CCCCGTCC | -94 | -87 | GCCCGTCC | 0.88 |
| XC_RS03205 | D | CCCGTCCC | -93 | -86 | CCCGTCCG | 0.88 |
| XC_RS03205 | D | CTCCCGTC | -95 | -88 | CGCCCGTC | 0.88 |
| XC_RS22260 | R | CCCCGTCC | -76 | -69 | CCCCGGCC | 0.88 |
| XC_RS22260 | R | CCCGTCCC | -100 | -93 | CCCGTCGC | 0.88 |
| XC_RS03220 | D | CCCCGTCC | -86 | -79 | CCCCGGCC | 0.88 |
| XC_RS03220 | D | CCCGTCCC | -62 | -55 | CCCGTCGC | 0.88 |
| XC_RS03225 | D | CCCGTCCC | -137 | -130 | CCCATCCC | 0.88 |
| XC_RS03240 | R | CCCCTCTC | -190 | -183 | CCCCGCTC | 0.88 |
| XC_RS03240 | R | CCCGTCCC | -185 | -178 | CCCGGCCC | 0.88 |
| XC_RS22265 | R | CCCCTCTC | -59 | -52 | CCCCTCCC | 0.88 |
| XC_RS22265 | R | CCCGTCCC | -59 | -52 | CCCCTCCC | 0.88 |
| XC_RS22265 | R | CTCCCGTC | -62 | -55 | CTCCCGAC | 0.88 |
| XC_RS03275 | R | CCCCTCTC | -9 | -2 | CCCCTCGC | 0.88 |
| XC_RS03285 | D | CCCCTCTC | -40 | -33 | CACCTCTC | 0.88 |
| XC_RS03285 | D | CCCCGTCC | -86 | -79 | CCCGGTCC | 0.88 |
| XC_RS03285 | D | CCCCGTCC | -67 | -60 | CCCAGTCC | 0.88 |
| XC_RS03285 | D | CCCGTCCC | -149 | -142 | CCCGGCCC | 0.88 |
| XC_RS03285 | D | CCTCTCTCCC | -38 | -29 | CCTCTCTGCC | 0.9 |
| XC_RS03290 | R | CCCCTCTC | -183 | -176 | CACCTCTC | 0.88 |
| XC_RS03290 | R | CCCCGTCC | -156 | -149 | CCCAGTCC | 0.88 |
| XC_RS03290 | R | CCCCGTCC | -137 | -130 | CCCGGTCC | 0.88 |
| XC_RS03290 | R | CCCGTCCC | -74 | -67 | CCCGGCCC | 0.88 |
| XC_RS03290 | R | CCTCTCTCCC | -187 | -178 | CCTCTCTGCC | 0.9 |
| XC_RS03295 | D | CTCCCGTC | -138 | -131 | CTGCCGTC | 0.88 |
| XC_RS03310 | D | CCCCTCTC | -57 | -50 | CCCCTCTA | 0.88 |
| XC_RS03310 | D | CTCCCGTC | -59 | -52 | CTCCCCTC | 0.88 |
| XC_RS22280 | D | CCCCGTCC | -84 | -77 | CGCCGTCC | 0.88 |
| XC_RS22280 | D | CCCGTCCC | -83 | -76 | GCCGTCCC | 0.88 |
| XC_RS22280 | D | CTCCCGTC | -153 | -146 | TTCCCGTC | 0.88 |
| XC_RS22280 | R | AGGCGGAGG | -174 | -166 | AGGCGGCGG | 0.89 |
| XC_RS22280 | D | CTCGCCGTC | -86 | -78 | CCCGCCGTC | 0.89 |
| XC_RS22280 | D | CCCCCGCCTC | -174 | -165 | CCGCCGCCTC | 0.9 |
| XC_RS03375 | D | CCCCGTCC | -69 | -62 | CCCCGGCC | 0.88 |
| XC_RS03415 | D | CCCCGTCC | -108 | -101 | CCCCGTCT | 0.88 |
| XC_RS03425 | R | CCCCTCTC | -16 | -9 | CCCCTCTC | 1 |
| XC_RS22290 | R | CTCGCCGTC | -116 | -108 | CTCACCGTC | 0.89 |
| XC_RS03480 | D | CCCCGTCC | -61 | -54 | CGCCGTCC | 0.88 |
| XC_RS03480 | D | CCCGTCCC | -60 | -53 | GCCGTCCC | 0.88 |
| XC_RS03480 | D | CTCGCCGTC | -63 | -55 | CGCGCCGTC | 0.89 |
| XC_RS22295 | R | CCCCTCTC | -152 | -145 | CCCCACTC | 0.88 |
| XC_RS22295 | R | CCCCGTCC | -172 | -165 | TCCCGTCC | 0.88 |
| XC_RS22295 | R | CCCGTCCC | -173 | -166 | CCCGTCCC | 1 |
| XC_RS22295 | D | CTCCCGTC | -97 | -90 | CTCCCGCC | 0.88 |
| XC_RS22295 | R | CTCCCGTC | -171 | -164 | TTCCCGTC | 0.88 |
| XC_RS03490 | D | CCCCTCTC | -34 | -27 | CCCCACTC | 0.88 |
| XC_RS03490 | D | CCCGTCCC | -13 | -6 | CCCGGCCC | 0.88 |
| XC_RS03525 | R | CCCCGTCC | -34 | -27 | CCCCGCCC | 0.88 |
| XC_RS22305 | R | AGGCGGAGG | -37 | -29 | AGGCGCAGG | 0.89 |
| XC_RS03560 | D | CTCGCCGTC | -120 | -112 | CTCGCCGGC | 0.89 |
| XC_RS03570 | R | CCCCTCTC | -113 | -106 | CCCCGCTC | 0.88 |
| XC_RS03575 | D | CCCGTCCC | -26 | -19 | CCTGTCCC | 0.88 |
| XC_RS03585 | R | CCCCGTCC | -94 | -87 | CCCCGTCG | 0.88 |
| XC_RS03585 | R | CCCCGTCC | -43 | -36 | CCCCGCCC | 0.88 |
| XC_RS03595 | D | CCCGTCCC | -11 | -4 | CCCGTACC | 0.88 |
| XC_RS03600 | D | CCCCGTCC | -21 | -14 | CCCCGTGC | 0.88 |
| XC_RS03600 | D | CCCGTCCC | -20 | -13 | CCCGTGCC | 0.88 |
| XC_RS03600 | R | CCCGTCCC | -146 | -139 | CCCGTACC | 0.88 |
| XC_RS03625 | D | CTCCCGTC | -25 | -18 | CTCCCGAC | 0.88 |
| XC_RS03625 | D | CTCCCGTC | -8 | -1 | CTTCCGTC | 0.88 |
| XC_RS03650 | D | CCCCGTCC | -59 | -52 | CCCCGGCC | 0.88 |
| XC_RS03650 | D | CCCGTCCC | -58 | -51 | CCCGGCCC | 0.88 |
| XC_RS03705 | D | CCCCGTCC | -93 | -86 | CCCCGTCA | 0.88 |
| XC_RS03705 | D | CCCGTCCC | -92 | -85 | CCCGTCAC | 0.88 |
| XC_RS03710 | R | CCCCGTCC | -53 | -46 | CCCCGTCA | 0.88 |
| XC_RS03710 | R | CCCGTCCC | -54 | -47 | CCCGTCAC | 0.88 |
| XC_RS03780 | D | CCCCTCTC | -149 | -142 | CCCGTCTC | 0.88 |
| XC_RS03780 | D | CCCGTCCC | -154 | -147 | ACCGTCCC | 0.88 |
| XC_RS03780 | D | CCCGTCCC | -120 | -113 | CCCATCCC | 0.88 |
| XC_RS03780 | D | CCCGTCCC | -74 | -67 | CCCGTCGC | 0.88 |
| XC_RS03780 | D | CTCCCGTC | -151 | -144 | GTCCCGTC | 0.88 |
| XC_RS03805 | D | CCCCGTCC | -121 | -114 | CGCCGTCC | 0.88 |
| XC_RS03805 | D | CCCGTCCC | -177 | -170 | GCCGTCCC | 0.88 |
| XC_RS03805 | D | CCCGTCCC | -120 | -113 | GCCGTCCC | 0.88 |
| XC_RS03820 | D | CCCCGTCC | -157 | -150 | CCCCGTGC | 0.88 |
| XC_RS03820 | D | CCCGTCCC | -156 | -149 | CCCGTGCC | 0.88 |
| XC_RS03825 | R | CCCCGTCC | -51 | -44 | CCCCGTGC | 0.88 |
| XC_RS03825 | R | CCCGTCCC | -52 | -45 | CCCGTGCC | 0.88 |
| XC_RS03850 | D | CCCCGTCC | -32 | -25 | CCCCTTCC | 0.88 |
| XC_RS03860 | R | CCCGTCCC | -85 | -78 | CCCGGCCC | 0.88 |
| XC_RS03865 | D | CCCGTCCC | -126 | -119 | CCCGGCCC | 0.88 |
| XC_RS03870 | D | CCCCTCTC | -24 | -17 | CCCCTTTC | 0.88 |
| XC_RS03870 | D | CCCCGTCC | -110 | -103 | CCCCGTCC | 1 |
| XC_RS03870 | D | CCCGTCCC | -163 | -156 | CCCGTGCC | 0.88 |
| XC_RS03870 | D | CCCGTCCC | -109 | -102 | CCCGTCCA | 0.88 |
| XC_RS03870 | D | CTCCCGTC | -165 | -158 | CTCCCGTG | 0.88 |
| XC_RS03880 | D | CCCCGTCC | -82 | -75 | TCCCGTCC | 0.88 |
| XC_RS03880 | D | CCCGTCCC | -81 | -74 | CCCGTCCT | 0.88 |
| XC_RS03880 | D | CTCCCGTC | -83 | -76 | CTCCCGTC | 1 |
| XC_RS03885 | R | CCCCGTCC | -61 | -54 | TCCCGTCC | 0.88 |
| XC_RS03885 | R | CCCGTCCC | -62 | -55 | CCCGTCCT | 0.88 |
| XC_RS03885 | R | CTCCCGTC | -60 | -53 | CTCCCGTC | 1 |
| XC_RS03905 | R | CCCGTCCC | -15 | -8 | CCCGTTCC | 0.88 |
| XC_RS03910 | D | CCCCGTCC | -37 | -30 | GCCCGTCC | 0.88 |
| XC_RS03910 | D | CCCGTCCC | -36 | -29 | CCCGTCCT | 0.88 |
| XC_RS03910 | D | CTCCCGTC | -38 | -31 | CGCCCGTC | 0.88 |
| XC_RS03915 | R | CCCCTCTC | -12 | -5 | CCCCTCCC | 0.88 |
| XC_RS03915 | R | CCCGTCCC | -12 | -5 | CCCCTCCC | 0.88 |
| XC_RS03915 | R | CTCGCCGTC | -63 | -55 | CTGGCCGTC | 0.89 |
| XC_RS22315 | R | CTCCCGTC | -31 | -24 | CTCCCGCC | 0.88 |
| XC_RS03950 | D | CCCCGTCC | -100 | -93 | GCCCGTCC | 0.88 |
| XC_RS03950 | R | CCCCGTCC | -13 | -6 | CCCCCTCC | 0.88 |
| XC_RS03950 | D | CCCGTCCC | -99 | -92 | CCCGTCCC | 1 |
| XC_RS03950 | D | AGGCGGAGG | -17 | -9 | AGCCGGAGG | 0.89 |
| XC_RS03950 | R | AGGCGGAGG | -70 | -62 | AGGCGAAGG | 0.89 |
| XC_RS03955 | R | CCCCGTCC | -182 | -175 | GCCCGTCC | 0.88 |
| XC_RS03955 | R | CCCGTCCC | -183 | -176 | CCCGTCCT | 0.88 |
| XC_RS03955 | R | CTCCCGTC | -181 | -174 | CGCCCGTC | 0.88 |
| XC_RS03975 | D | CTCGCCGTC | -160 | -152 | ATCGCCGTC | 0.89 |
| XC_RS04005 | D | CTCCCGTC | -26 | -19 | CTCCCATC | 0.88 |
| XC_RS04060 | R | CCCCTCTC | -38 | -31 | CCCCTCCC | 0.88 |
| XC_RS04060 | R | CCCGTCCC | -38 | -31 | CCCCTCCC | 0.88 |
| XC_RS04075 | D | CCCCTCTC | -45 | -38 | CCCCACTC | 0.88 |
| XC_RS04075 | D | CCCCTTCTCC | -62 | -53 | CCCCTGCTCC | 0.9 |
| XC_RS04080 | D | CTCCCGTC | -164 | -157 | CTGCCGTC | 0.88 |
| XC_RS04085 | D | CCCCTCTC | -115 | -108 | CCCGTCTC | 0.88 |
| XC_RS04085 | D | CCCGTCCC | -115 | -108 | CCCGTCTC | 0.88 |
| XC_RS04085 | D | CTCCCGTC | -179 | -172 | CTCCCATC | 0.88 |
| XC_RS04085 | D | CTCCCGTC | -117 | -110 | ATCCCGTC | 0.88 |
| XC_RS04085 | D | CTCGCCGTC | -64 | -56 | CTCGCCGTG | 0.89 |
| XC_RS04090 | R | CCCCTCTC | -159 | -152 | CCCGTCTC | 0.88 |
| XC_RS04090 | R | CCCGTCCC | -159 | -152 | CCCGTCTC | 0.88 |
| XC_RS04090 | R | CTCCCGTC | -157 | -150 | ATCCCGTC | 0.88 |
| XC_RS04090 | R | CTCCCGTC | -95 | -88 | CTCCCATC | 0.88 |
| XC_RS04115 | D | AGGCGGAGG | -119 | -111 | AGGCGCAGG | 0.89 |
| XC_RS04120 | R | CCCCGTCC | -64 | -57 | CCGCGTCC | 0.88 |
| XC_RS04120 | R | CCCGTCCC | -65 | -58 | CGCGTCCC | 0.88 |
| XC_RS04125 | D | CCCGTCCC | -95 | -88 | GCCGTCCC | 0.88 |
| XC_RS04125 | D | CCCGTCCC | -36 | -29 | CCCGGCCC | 0.88 |
| XC_RS04140 | D | CCCGTCCC | -47 | -40 | CCCGCCCC | 0.88 |
| XC_RS04140 | R | CTCCCGTC | -142 | -135 | CTCGCGTC | 0.88 |
| XC_RS04140 | R | AGGCGGAGG | -27 | -19 | AGGCGGATG | 0.89 |
| XC_RS04160 | D | CCCCGTCC | -190 | -183 | TCCCGTCC | 0.88 |
| XC_RS04160 | D | CCCGTCCC | -189 | -182 | CCCGTCCT | 0.88 |
| XC_RS04160 | D | CTCCCGTC | -191 | -184 | GTCCCGTC | 0.88 |
| XC_RS04165 | D | CTCCCGTC | -18 | -11 | CTCCCGTA | 0.88 |
| XC_RS04165 | D | AGGCGGAGG | -190 | -182 | AGGCGGATG | 0.89 |
| XC_RS04190 | R | CCCCGTCC | -100 | -93 | CGCCGTCC | 0.88 |
| XC_RS04195 | D | CCCCGTCC | -32 | -25 | CGCCGTCC | 0.88 |
| XC_RS04200 | D | CTCCCGTC | -53 | -46 | CTCCCGCC | 0.88 |
| XC_RS04200 | D | CCCCCGCCTC | -53 | -44 | CTCCCGCCTC | 0.9 |
| XC_RS04230 | R | AGGCGGAGG | -43 | -35 | AGGCGGCGG | 0.89 |
| XC_RS04230 | D | CCCCCGCCTC | -43 | -34 | CCGCCGCCTC | 0.9 |
| XC_RS04240 | R | CCCCGTCC | -69 | -62 | CCACGTCC | 0.88 |
| XC_RS04250 | D | CCTCTCTCCC | -105 | -96 | TCTCTCTCCC | 0.9 |
| XC_RS04260 | D | CCCCTCTC | -57 | -50 | CCCCTCGC | 0.88 |
| XC_RS04285 | D | AGGCGGAGG | -90 | -82 | AGACGGAGG | 0.89 |
| XC_RS04290 | R | AGGCGGAGG | -118 | -110 | AGACGGAGG | 0.89 |
| XC_RS04295 | R | CCCCTCTC | -10 | -3 | CCCCTCCC | 0.88 |
| XC_RS04295 | R | CCCGTCCC | -10 | -3 | CCCCTCCC | 0.88 |
| XC_RS04385 | D | CCCCTCTC | -35 | -28 | CCCCTCTT | 0.88 |
| XC_RS04385 | D | CTCCCGTC | -195 | -188 | CTCCAGTC | 0.88 |
| XC_RS04425 | R | CCCCTCTC | -112 | -105 | CCACTCTC | 0.88 |
| XC_RS04430 | D | CCCCTCTC | -189 | -182 | CCCCTTTC | 0.88 |
| XC_RS04435 | D | AGGCGGAGG | -56 | -48 | AAGCGGAGG | 0.89 |
| XC_RS04440 | R | AGGCGGAGG | -25 | -17 | AAGCGGAGG | 0.89 |
| XC_RS04480 | R | CCCGTCCC | -151 | -144 | CCCATCCC | 0.88 |
| XC_RS04480 | D | CCCCCGCCTC | -10 | -1 | CCCTCGCCTC | 0.9 |
| XC_RS04485 | D | CCCCTCTC | -52 | -45 | CCCCGCTC | 0.88 |
| XC_RS04485 | D | AGGCGGAGG | -93 | -85 | AGGCGGCGG | 0.89 |
| XC_RS22325 | D | CCCCGTCC | -59 | -52 | CCGCGTCC | 0.88 |
| XC_RS22325 | R | CCTCTCTCCC | -10 | -1 | GCTCTCTCCC | 0.9 |
| XC_RS04495 | D | CCCGTCCC | -33 | -26 | CCCATCCC | 0.88 |
| XC_RS04500 | R | CCCGTCCC | -82 | -75 | CCCATCCC | 0.88 |
| XC_RS04505 | D | CTCCCGTC | -22 | -15 | CTCCTGTC | 0.88 |
| XC_RS04510 | D | CCCCTCTC | -24 | -17 | CCCCCCTC | 0.88 |
| XC_RS04510 | D | CCCCGTCC | -23 | -16 | CCCCCTCC | 0.88 |
| XC_RS04535 | D | CCCCTCTC | -16 | -9 | CCCCTATC | 0.88 |
| XC_RS22360 | R | CCCCTCTC | -80 | -73 | CCCCTCAC | 0.88 |
| XC_RS22360 | R | CCCCGTCC | -130 | -123 | CCCCATCC | 0.88 |
| XC_RS04570 | D | CCCCTCTC | -68 | -61 | CCCCTCAC | 0.88 |
| XC_RS04570 | D | CCCCGTCC | -18 | -11 | CCCCATCC | 0.88 |
| XC_RS04615 | D | CCCCTCTC | -32 | -25 | GCCCTCTC | 0.88 |
| XC_RS04615 | R | CCCGTCCC | -41 | -34 | CCCGGCCC | 0.88 |
| XC_RS04640 | D | CCCGTCCC | -73 | -66 | CCCGTTCC | 0.88 |
| XC_RS04640 | R | AGGCGGAGG | -67 | -59 | AGGGGGAGG | 0.89 |
| XC_RS04660 | R | AGGCGGAGG | -21 | -13 | AAGCGGAGG | 0.89 |
| XC_RS04665 | R | CCCCTCTC | -86 | -79 | CGCCTCTC | 0.88 |
| XC_RS04695 | R | CCCCGTCC | -97 | -90 | CCCCGGCC | 0.88 |
| XC_RS04700 | R | CCCCTCTC | -16 | -9 | CCCCTATC | 0.88 |
| XC_RS04730 | D | CCCCGTCC | -97 | -90 | CCGCGTCC | 0.88 |
| XC_RS04735 | D | CCCCTCTC | -148 | -141 | CCCCTCAC | 0.88 |
| XC_RS04745 | R | CCCCTCTC | -106 | -99 | CCCCACTC | 0.88 |
| XC_RS04745 | R | CCCCTCTC | -90 | -83 | CCCCGCTC | 0.88 |
| XC_RS04785 | R | AGGCGGAGG | -24 | -16 | AGGCGGGGG | 0.89 |
| XC_RS04785 | D | CCCCCGCCTC | -24 | -15 | CCCCCGCCTA | 0.9 |
| XC_RS04800 | D | CCCCTCTC | -50 | -43 | CCCCTTTC | 0.88 |
| XC_RS04800 | R | CTCCCGTC | -13 | -6 | CTCCCGGC | 0.88 |
| XC_RS04810 | R | CTCCCGTC | -97 | -90 | CTCCCGAC | 0.88 |
| XC_RS04815 | D | CTCCCGTC | -23 | -16 | CTCCCGAC | 0.88 |
| XC_RS04835 | R | CCCCGTCC | -64 | -57 | CCGCGTCC | 0.88 |
| XC_RS22390 | D | AGGCGGAGG | -30 | -22 | AGGCGGGGG | 0.89 |
| XC_RS22390 | R | CCCCCGCCTC | -31 | -22 | CCCCCGCCTG | 0.9 |
| XC_RS04855 | R | CCCCGTCC | -119 | -112 | CGCCGTCC | 0.88 |
| XC_RS04855 | R | CCCGTCCC | -75 | -68 | CACGTCCC | 0.88 |
| XC_RS04855 | R | CTCGCCGTC | -118 | -110 | GTCGCCGTC | 0.89 |
| XC_RS04860 | D | CCCCTCTC | -170 | -163 | CCCCTCCC | 0.88 |
| XC_RS04860 | D | CCCCGTCC | -171 | -164 | CCCCCTCC | 0.88 |
| XC_RS04860 | D | CCCGTCCC | -170 | -163 | CCCCTCCC | 0.88 |
| XC_RS04860 | D | CTCGCCGTC | -102 | -94 | CGCGCCGTC | 0.89 |
| XC_RS04870 | D | CCCCTCTC | -46 | -39 | CCCCTTTC | 0.88 |
| XC_RS04900 | D | CTCCCGTC | -150 | -143 | CTGCCGTC | 0.88 |
| XC_RS04905 | R | CTCCCGTC | -169 | -162 | CTCACGTC | 0.88 |
| XC_RS04915 | D | CTCCCGTC | -57 | -50 | CTCCCGCC | 0.88 |
| XC_RS04915 | R | CCTCTCTCCC | -12 | -3 | CTTCTCTCCC | 0.9 |
| XC_RS04925 | D | CCCCGTCC | -8 | -1 | CCCGGTCC | 0.88 |
| XC_RS04925 | D | CTCCCGTC | -21 | -14 | CTCCCCTC | 0.88 |
| XC_RS04930 | R | CCCCGTCC | -163 | -156 | CCCGGTCC | 0.88 |
| XC_RS04930 | R | CTCCCGTC | -150 | -143 | CTCCCCTC | 0.88 |
| XC_RS04965 | D | CCCCTCTC | -31 | -24 | CCCCTTTC | 0.88 |
| XC_RS04965 | R | CCCCTCTC | -102 | -95 | CCCCACTC | 0.88 |
| XC_RS04970 | R | CTCGCCGTC | -160 | -152 | CTCGCCGCC | 0.89 |
| XC_RS04975 | D | CTCGCCGTC | -27 | -19 | CTCGCCGCC | 0.89 |
| XC_RS04985 | R | CCCGTCCC | -187 | -180 | CCCGTACC | 0.88 |
| XC_RS05020 | D | AGGCGGAGG | -14 | -6 | AGGAGGAGG | 0.89 |
| XC_RS05025 | R | AGGCGGAGG | -167 | -159 | AGGAGGAGG | 0.89 |
| XC_RS05030 | R | CCCCTCTC | -11 | -4 | CCCCTCCC | 0.88 |
| XC_RS05030 | R | CCCGTCCC | -11 | -4 | CCCCTCCC | 0.88 |
| XC_RS05050 | R | CCCCGTCC | -59 | -52 | CCCCGTCT | 0.88 |
| XC_RS05050 | R | CTCCCGTC | -58 | -51 | CCCCCGTC | 0.88 |
| XC_RS05055 | D | CCCCGTCC | -25 | -18 | CCGCGTCC | 0.88 |
| XC_RS05055 | D | CCCGTCCC | -24 | -17 | CGCGTCCC | 0.88 |
| XC_RS05070 | R | AGGCGGAGG | -88 | -80 | AGGCGGCGG | 0.89 |
| XC_RS05080 | D | CCCCTCTC | -48 | -41 | CCCCTCTC | 1 |
| XC_RS05080 | R | AGGCGGAGG | -112 | -104 | AGGCGCAGG | 0.89 |
| XC_RS05080 | D | CCTCTCTCCC | -48 | -39 | CCCCTCTCCC | 0.9 |
| XC_RS05090 | D | CCCCGTCC | -77 | -70 | CGCCGTCC | 0.88 |
| XC_RS05090 | D | CTCGCCGTC | -79 | -71 | CTCGCCGTC | 1 |
| XC_RS05095 | R | CCCCGTCC | -119 | -112 | CGCCGTCC | 0.88 |
| XC_RS05095 | R | CTCGCCGTC | -118 | -110 | CTCGCCGTC | 1 |
| XC_RS05100 | R | CCCGTCCC | -80 | -73 | CCCGGCCC | 0.88 |
| XC_RS05105 | D | CCCCTCTC | -162 | -155 | CCCCTCTT | 0.88 |
| XC_RS05105 | D | CCCGTCCC | -73 | -66 | CCCGTTCC | 0.88 |
| XC_RS05140 | R | CCCCGTCC | -53 | -46 | CCCCGCCC | 0.88 |
| XC_RS05165 | D | CCCCTCTC | -120 | -113 | ACCCTCTC | 0.88 |
| XC_RS05165 | R | CTCCCGTC | -158 | -151 | CTTCCGTC | 0.88 |
| XC_RS05255 | R | AGGCGGAGG | -128 | -120 | AGGCGGAGG | 1 |
| XC_RS05260 | R | CCCCTCTC | -52 | -45 | CCCCGCTC | 0.88 |
| XC_RS05260 | D | CCCCGTCC | -173 | -166 | CGCCGTCC | 0.88 |
| XC_RS05260 | D | CTCGCCGTC | -175 | -167 | CACGCCGTC | 0.89 |
| XC_RS05260 | D | CTCGCCGTC | -76 | -68 | CCCGCCGTC | 0.89 |
| XC_RS05275 | R | CCCGTCCC | -70 | -63 | CCCGTTCC | 0.88 |
| XC_RS05280 | R | CTCCCGTC | -113 | -106 | CTGCCGTC | 0.88 |
| XC_RS05305 | D | AGGCGGAGG | -169 | -161 | AGGCAGAGG | 0.89 |
| XC_RS05320 | D | CCCCGTCC | -102 | -95 | CCCCTTCC | 0.88 |
| XC_RS22425 | R | CCCCTCTC | -192 | -185 | CCCCACTC | 0.88 |
| XC_RS22425 | R | CCCCGTCC | -86 | -79 | CCCCGCCC | 0.88 |
| XC_RS05345 | R | CCCCGTCC | -188 | -181 | CCCCGTCG | 0.88 |
| XC_RS05345 | R | CTCCCGTC | -173 | -166 | CTCCCGAC | 0.88 |
| XC_RS05350 | D | CCCCGTCC | -53 | -46 | CCCCGTCG | 0.88 |
| XC_RS05350 | D | CTCCCGTC | -75 | -68 | CTCGCGTC | 0.88 |
| XC_RS05380 | R | CCCCGTCC | -122 | -115 | CCGCGTCC | 0.88 |
| XC_RS05380 | R | CTCCCGTC | -77 | -70 | CTCCCGCC | 0.88 |
| XC_RS05385 | D | CCCCGTCC | -8 | -1 | CCGCGTCC | 0.88 |
| XC_RS05385 | D | CTCCCGTC | -53 | -46 | CTCCCGCC | 0.88 |
| XC_RS05405 | R | CTCCCGTC | -200 | -193 | CTCCCGGC | 0.88 |
| XC_RS05410 | D | CCCCTCTC | -62 | -55 | CCTCTCTC | 0.88 |
| XC_RS05410 | R | CCCCTCTC | -114 | -107 | CCCCTCCC | 0.88 |
| XC_RS05410 | R | CCCGTCCC | -114 | -107 | CCCCTCCC | 0.88 |
| XC_RS05410 | D | CCTCTCTCCC | -62 | -53 | CCTCTCTCCC | 1 |
| XC_RS22430 | D | CTCGCCGTC | -89 | -81 | CTCGCTGTC | 0.89 |
| XC_RS05425 | D | CCCCTCTC | -185 | -178 | CCCCTCTC | 1 |
| XC_RS05425 | R | CCCCTCTC | -171 | -164 | CCCCTCTC | 1 |
| XC_RS05425 | R | CCCCTCTC | -64 | -57 | CCCTTCTC | 0.88 |
| XC_RS05425 | D | CCCGTCCC | -107 | -100 | CCCGTACC | 0.88 |
| XC_RS05425 | D | CTCCCGTC | -74 | -67 | CTCCCGAC | 0.88 |
| XC_RS05425 | R | CTCCCGTC | -69 | -62 | CTCCCGTC | 1 |
| XC_RS05425 | D | CCCCTTCTCC | -80 | -71 | CACCTTCTCC | 0.9 |
| XC_RS05425 | R | CCCCTTCTCC | -65 | -56 | TCCCTTCTCC | 0.9 |
| XC_RS05425 | R | CCTCTCTCCC | -173 | -164 | CCCCTCTCCC | 0.9 |
| XC_RS05430 | D | CCCGTCCC | -73 | -66 | CCCGGCCC | 0.88 |
| XC_RS05430 | D | CTCCCGTC | -75 | -68 | CTCCCGGC | 0.88 |
| XC_RS05430 | D | AGGCGGAGG | -194 | -186 | AGGCGGGGG | 0.89 |
| XC_RS05430 | R | CCCCCGCCTC | -195 | -186 | CCCCCGCCTG | 0.9 |
| XC_RS05435 | R | CCCGTCCC | -172 | -165 | CCCGGCCC | 0.88 |
| XC_RS05435 | R | CTCCCGTC | -170 | -163 | CTCCCGGC | 0.88 |
| XC_RS05435 | R | AGGCGGAGG | -52 | -44 | AGGCGGGGG | 0.89 |
| XC_RS05435 | D | CCCCCGCCTC | -52 | -43 | CCCCCGCCTG | 0.9 |
| XC_RS05445 | D | CCCGTCCC | -25 | -18 | CCTGTCCC | 0.88 |
| XC_RS05480 | D | AGGCGGAGG | -79 | -71 | TGGCGGAGG | 0.89 |
| XC_RS05485 | R | AGGCGGAGG | -23 | -15 | AGGAGGAGG | 0.89 |
| XC_RS05495 | D | CTCCCGTC | -51 | -44 | CTCGCGTC | 0.88 |
| XC_RS05500 | R | CCCGTCCC | -14 | -7 | CCCGTCAC | 0.88 |
| XC_RS22435 | R | CTCCCGTC | -14 | -7 | CTCCAGTC | 0.88 |
| XC_RS05580 | D | CCCGTCCC | -24 | -17 | CCCGTTCC | 0.88 |
| XC_RS05585 | D | CCCCTCTC | -113 | -106 | CCCCTCGC | 0.88 |
| XC_RS05585 | D | CTCGCCGTC | -36 | -28 | CTCGCCGTC | 1 |
| XC_RS05600 | D | CCCCTCTC | -22 | -15 | CCCCACTC | 0.88 |
| XC_RS05600 | R | CCCCGTCC | -62 | -55 | CCGCGTCC | 0.88 |
| XC_RS05600 | R | CCCCGTCC | -50 | -43 | CCCCTTCC | 0.88 |
| XC_RS05600 | R | CCCGTCCC | -51 | -44 | CCCTTCCC | 0.88 |
| XC_RS05605 | D | CCCCGTCC | -190 | -183 | CCCCGGCC | 0.88 |
| XC_RS05605 | D | CTCCCGTC | -53 | -46 | CTCCTGTC | 0.88 |
| XC_RS05610 | D | CCCCGTCC | -54 | -47 | CCCCGGCC | 0.88 |
| XC_RS05615 | R | CCCCGTCC | -132 | -125 | CCCCGGCC | 0.88 |
| XC_RS05650 | D | CCCCGTCC | -18 | -11 | CCCCGCCC | 0.88 |
| XC_RS22445 | R | CTCCCGTC | -29 | -22 | CGCCCGTC | 0.88 |
| XC_RS05670 | D | CCCCTCTC | -30 | -23 | CGCCTCTC | 0.88 |
| XC_RS05670 | R | CCCCGTCC | -133 | -126 | CGCCGTCC | 0.88 |
| XC_RS05670 | R | CTCCCGTC | -93 | -86 | CTCCCGCC | 0.88 |
| XC_RS05670 | D | AGGCGGAGG | -82 | -74 | ATGCGGAGG | 0.89 |
| XC_RS05675 | R | CCCCTCTC | -140 | -133 | CCTCTCTC | 0.88 |
| XC_RS05675 | D | CCCCGTCC | -35 | -28 | CGCCGTCC | 0.88 |
| XC_RS05675 | D | CTCCCGTC | -75 | -68 | CTCCCGCC | 0.88 |
| XC_RS05675 | R | AGGCGGAGG | -87 | -79 | ATGCGGAGG | 0.89 |
| XC_RS05680 | D | CCCCGTCC | -77 | -70 | CACCGTCC | 0.88 |
| XC_RS05680 | D | CCCGTCCC | -76 | -69 | ACCGTCCC | 0.88 |
| XC_RS05685 | R | AGGCGGAGG | -11 | -3 | CGGCGGAGG | 0.89 |
| XC_RS05695 | D | CCCCTCTC | -19 | -12 | CCCCACTC | 0.88 |
| XC_RS05695 | D | CCCGTCCC | -12 | -5 | CCCGGCCC | 0.88 |
| XC_RS05695 | D | CTCCCGTC | -14 | -7 | CTCCCGGC | 0.88 |
| XC_RS05705 | D | CCCCGTCC | -130 | -123 | CCCCGTAC | 0.88 |
| XC_RS05705 | D | CTCGCCGTC | -49 | -41 | CACGCCGTC | 0.89 |
| XC_RS05740 | R | CCCCGTCC | -174 | -167 | CCGCGTCC | 0.88 |
| XC_RS05810 | D | CCCCGTCC | -74 | -67 | CACCGTCC | 0.88 |
| XC_RS05810 | D | CTCGCCGTC | -76 | -68 | CTCACCGTC | 0.89 |
| XC_RS05820 | R | CCCGTCCC | -109 | -102 | CCCGGCCC | 0.88 |
| XC_RS05825 | D | CCCGTCCC | -26 | -19 | CCCGGCCC | 0.88 |
| XC_RS05840 | D | CCCGTCCC | -26 | -19 | CCAGTCCC | 0.88 |
| XC_RS05845 | D | CTCGCCGTC | -182 | -174 | CTGGCCGTC | 0.89 |
| XC_RS05860 | D | CCCCGTCC | -37 | -30 | CGCCGTCC | 0.88 |
| XC_RS05860 | D | CTCCCGTC | -95 | -88 | CTCGCGTC | 0.88 |
| XC_RS05860 | D | CTCGCCGTC | -39 | -31 | CCCGCCGTC | 0.89 |
| XC_RS05860 | R | CTCGCCGTC | -83 | -75 | CTCGCGGTC | 0.89 |
| XC_RS05875 | D | CCCCTCTC | -24 | -17 | CGCCTCTC | 0.88 |
| XC_RS05875 | D | CCTCTCTCCC | -22 | -13 | CCTCTCTCTC | 0.9 |
| XC_RS05895 | D | CCCCTCTC | -55 | -48 | GCCCTCTC | 0.88 |
| XC_RS05905 | D | CCCCGTCC | -134 | -127 | CCCCGTCA | 0.88 |
| XC_RS05905 | D | CCCGTCCC | -139 | -132 | CGCGTCCC | 0.88 |
| XC_RS05925 | D | CCCCGTCC | -128 | -121 | GCCCGTCC | 0.88 |
| XC_RS05925 | D | CCCGTCCC | -127 | -120 | CCCGTCCT | 0.88 |
| XC_RS05930 | D | CCCCGTCC | -28 | -21 | CCCGGTCC | 0.88 |
| XC_RS05930 | D | CCCGTCCC | -175 | -168 | CCCGTCAC | 0.88 |
| XC_RS05930 | D | CCCGTCCC | -27 | -20 | CCGGTCCC | 0.88 |
| XC_RS05930 | D | CTCCCGTC | -177 | -170 | CTCCCGTC | 1 |
| XC_RS05930 | R | AGGCGGAGG | -118 | -110 | TGGCGGAGG | 0.89 |
| XC_RS05945 | R | CCCCGTCC | -93 | -86 | CCCGGTCC | 0.88 |
| XC_RS05945 | D | AGGCGGAGG | -126 | -118 | AGGCGGACG | 0.89 |
| XC_RS05950 | D | CCCCGTCC | -152 | -145 | CCCGGTCC | 0.88 |
| XC_RS05950 | R | AGGCGGAGG | -120 | -112 | AGGCGGACG | 0.89 |
| XC_RS05970 | R | CCCGTCCC | -53 | -46 | CCCGTTCC | 0.88 |
| XC_RS06030 | R | CCCCTCTC | -131 | -124 | CCGCTCTC | 0.88 |
| XC_RS06030 | R | CCTCTCTCCC | -133 | -124 | CCGCTCTCCC | 0.9 |
| XC_RS06040 | D | CTCCCGTC | -52 | -45 | TTCCCGTC | 0.88 |
| XC_RS06040 | R | AGGCGGAGG | -150 | -142 | AGGCGGCGG | 0.89 |
| XC_RS06055 | R | CCCCGTCC | -160 | -153 | CCCCGTCG | 0.88 |
| XC_RS06055 | R | CCCGTCCC | -161 | -154 | CCCGTCGC | 0.88 |
| XC_RS06055 | R | AGGCGGAGG | -183 | -175 | AGGCGGTGG | 0.89 |
| XC_RS06055 | R | CTCGCCGTC | -91 | -83 | CTGGCCGTC | 0.89 |
| XC_RS06070 | D | CTCCCGTC | -154 | -147 | CTCCCATC | 0.88 |
| XC_RS06070 | D | CTCCCGTC | -127 | -120 | CTCACGTC | 0.88 |
| XC_RS06075 | R | CTCCCGTC | -41 | -34 | CTCACGTC | 0.88 |
| XC_RS06075 | R | CTCCCGTC | -14 | -7 | CTCCCATC | 0.88 |
| XC_RS06080 | D | CCCCTCTC | -87 | -80 | CCCGTCTC | 0.88 |
| XC_RS06080 | D | CCCGTCCC | -87 | -80 | CCCGTCTC | 0.88 |
| XC_RS06080 | D | CTCCCGTC | -182 | -175 | CTCCCGAC | 0.88 |
| XC_RS06095 | D | CCCCTCTC | -33 | -26 | CCCCCCTC | 0.88 |
| XC_RS06105 | D | CCCCTCTC | -81 | -74 | CCCTTCTC | 0.88 |
| XC_RS06105 | D | CTCCCGTC | -76 | -69 | CTCCCGCC | 0.88 |
| XC_RS06105 | D | CCCCTTCTCC | -82 | -73 | TCCCTTCTCC | 0.9 |
| XC_RS06115 | D | CCCGTCCC | -69 | -62 | CCCGTCGC | 0.88 |
| XC_RS06115 | R | CCCGTCCC | -58 | -51 | CCCGTCGC | 0.88 |
| XC_RS06125 | D | CCCGTCCC | -139 | -132 | CCAGTCCC | 0.88 |
| XC_RS06135 | D | CCCGTCCC | -73 | -66 | CCAGTCCC | 0.88 |
| XC_RS06135 | R | CTCGCCGTC | -196 | -188 | CGCGCCGTC | 0.89 |
| XC_RS06145 | D | CTCGCCGTC | -60 | -52 | CTCGCCGTT | 0.89 |
| XC_RS06180 | R | CCCCTCTC | -200 | -193 | CCCCTCGC | 0.88 |
| XC_RS06180 | D | CTCCCGTC | -85 | -78 | CTCCCGCC | 0.88 |
| XC_RS06190 | D | CTCCCGTC | -186 | -179 | CGCCCGTC | 0.88 |
| XC_RS06190 | D | CCTCTCTCCC | -24 | -15 | CCTCTTTCCC | 0.9 |
| XC_RS22475 | D | CCCGTCCC | -114 | -107 | TCCGTCCC | 0.88 |
| XC_RS22475 | D | CTCCCGTC | -111 | -104 | GTCCCGTC | 0.88 |
| XC_RS06215 | D | CCCGTCCC | -9 | -2 | CCAGTCCC | 0.88 |
| XC_RS06280 | D | CCCCTCTC | -36 | -29 | CCCCTCTC | 1 |
| XC_RS06280 | D | CCCGTCCC | -167 | -160 | CCTGTCCC | 0.88 |
| XC_RS06280 | R | AGGCGGAGG | -161 | -153 | CGGCGGAGG | 0.89 |
| XC_RS06280 | R | AGGCGGAGG | -129 | -121 | AGGCGGTGG | 0.89 |
| XC_RS06280 | D | CCTCTCTCCC | -34 | -25 | CCTCTCGCCC | 0.9 |
| XC_RS06285 | D | CCCCGTCC | -94 | -87 | CCGCGTCC | 0.88 |
| XC_RS06285 | R | CCCGTCCC | -172 | -165 | CCTGTCCC | 0.88 |
| XC_RS06285 | D | AGGCGGAGG | -179 | -171 | CGGCGGAGG | 0.89 |
| XC_RS06285 | R | AGGCGGAGG | -97 | -89 | ACGCGGAGG | 0.89 |
| XC_RS06325 | D | CCCCGTCC | -83 | -76 | CCACGTCC | 0.88 |
| XC_RS06330 | R | CCCCGTCC | -21 | -14 | CCACGTCC | 0.88 |
| XC_RS06340 | D | CCCGTCCC | -99 | -92 | CCCGTCGC | 0.88 |
| XC_RS06345 | D | CCCCGTCC | -24 | -17 | CCCCGTCC | 1 |
| XC_RS06345 | D | CCCGTCCC | -23 | -16 | CCCGTCCT | 0.88 |
| XC_RS06355 | D | CCCCGTCC | -157 | -150 | CGCCGTCC | 0.88 |
| XC_RS06360 | D | CTCCCGTC | -137 | -130 | CTCCCGGC | 0.88 |
| XC_RS06370 | R | CTCCCGTC | -65 | -58 | CTCCCGGC | 0.88 |
| XC_RS06375 | D | CTCCCGTC | -137 | -130 | CTCCCGGC | 0.88 |
| XC_RS06400 | D | CTCCCGTC | -29 | -22 | CACCCGTC | 0.88 |
| XC_RS06440 | R | CCCGTCCC | -190 | -183 | CCCGTCGC | 0.88 |
| XC_RS06445 | R | CCCCGTCC | -92 | -85 | CCCCATCC | 0.88 |
| XC_RS06460 | R | CCCCTCTC | -118 | -111 | CCCCTCGC | 0.88 |
| XC_RS22500 | D | CCCCGTCC | -13 | -6 | CCACGTCC | 0.88 |
| XC_RS06470 | R | CCCCGTCC | -65 | -58 | CCACGTCC | 0.88 |
| XC_RS06490 | D | CCCGTCCC | -73 | -66 | CCCGCCCC | 0.88 |
| XC_RS06490 | D | CCCGTCCC | -28 | -21 | CCCGGCCC | 0.88 |
| XC_RS06495 | R | CCCGTCCC | -175 | -168 | CCCTTCCC | 0.88 |
| XC_RS06495 | R | CCCGTCCC | -125 | -118 | CCCGCCCC | 0.88 |
| XC_RS06500 | D | CCCCTCTC | -31 | -24 | CCGCTCTC | 0.88 |
| XC_RS06500 | D | CCCGTCCC | -58 | -51 | CCCGACCC | 0.88 |
| XC_RS06510 | D | CTCCCGTC | -24 | -17 | TTCCCGTC | 0.88 |
| XC_RS06510 | D | AGGCGGAGG | -86 | -78 | AGGCGGTGG | 0.89 |
| XC_RS06510 | R | CCCCCGCCTC | -87 | -78 | CCACCGCCTC | 0.9 |
| XC_RS06515 | D | CCCGTCCC | -119 | -112 | CGCGTCCC | 0.88 |
| XC_RS06515 | R | CCCGTCCC | -131 | -124 | CCCGTTCC | 0.88 |
| XC_RS06550 | D | CCCCGTCC | -14 | -7 | CCGCGTCC | 0.88 |
| XC_RS06590 | D | CCCCGTCC | -24 | -17 | CCCCGTTC | 0.88 |
| XC_RS06605 | R | AGGCGGAGG | -43 | -35 | ATGCGGAGG | 0.89 |
| XC_RS06620 | D | CCCCTCTC | -43 | -36 | TCCCTCTC | 0.88 |
| XC_RS06620 | D | CCCCGTCC | -21 | -14 | CCCCGTGC | 0.88 |
| XC_RS06620 | D | CCCGTCCC | -20 | -13 | CCCGTGCC | 0.88 |
| XC_RS06620 | D | CCTCTCTCCC | -14 | -5 | CCTCGCTCCC | 0.9 |
| XC_RS06715 | D | CCCCGTCC | -38 | -31 | CGCCGTCC | 0.88 |
| XC_RS06715 | R | CCCCGTCC | -21 | -14 | CGCCGTCC | 0.88 |
| XC_RS06715 | D | CTCGCCGTC | -40 | -32 | CGCGCCGTC | 0.89 |
| XC_RS06715 | R | CTCGCCGTC | -20 | -12 | CACGCCGTC | 0.89 |
| XC_RS06720 | D | CCCCTCTC | -183 | -176 | CCCCTGTC | 0.88 |
| XC_RS06720 | D | CCCCGTCC | -182 | -175 | CCCTGTCC | 0.88 |
| XC_RS06720 | D | CCCGTCCC | -63 | -56 | CCCTTCCC | 0.88 |
| XC_RS06730 | R | CCCCTCTC | -168 | -161 | CCCCTCTG | 0.88 |
| XC_RS06730 | D | CTCCCGTC | -55 | -48 | CTCCCGAC | 0.88 |
| XC_RS06745 | D | CCCCTCTC | -156 | -149 | CCCTTCTC | 0.88 |
| XC_RS06745 | D | CCCCTCTC | -52 | -45 | CCCCTCTC | 1 |
| XC_RS06745 | R | CCCCTCTC | -37 | -30 | CCCCTCTC | 1 |
| XC_RS06745 | R | CCCGTCCC | -113 | -106 | CCCGTACC | 0.88 |
| XC_RS06745 | D | CTCCCGTC | -151 | -144 | CTCCCCTC | 0.88 |
| XC_RS06745 | D | CTCCCGTC | -47 | -40 | CTCCCGGC | 0.88 |
| XC_RS06745 | D | CCCCTTCTCC | -157 | -148 | CCCCTTCTCC | 1 |
| XC_RS06745 | R | CCCCTTCTCC | -142 | -133 | CACCTTCTCC | 0.9 |
| XC_RS06745 | D | CCTCTCTCCC | -52 | -43 | CCCCTCTCCC | 0.9 |
| XC_RS06755 | D | CCCCGTCC | -12 | -5 | CACCGTCC | 0.88 |
| XC_RS06760 | R | CCCCGTCC | -169 | -162 | CACCGTCC | 0.88 |
| XC_RS06775 | R | CCCCGTCC | -15 | -8 | CCCCGTCA | 0.88 |
| XC_RS06775 | R | CCCGTCCC | -16 | -9 | CCCGTCAC | 0.88 |
| XC_RS06775 | R | CTCGCCGTC | -14 | -6 | CTCCCCGTC | 0.89 |
| XC_RS06785 | R | AGGCGGAGG | -76 | -68 | AGGCGGAGA | 0.89 |
| XC_RS06790 | D | CCCGTCCC | -134 | -127 | CCCGTGCC | 0.88 |
| XC_RS06800 | R | CCCGTCCC | -48 | -41 | CCCGTGCC | 0.88 |
| XC_RS06805 | R | CCCCGTCC | -45 | -38 | CCCCGGCC | 0.88 |
| XC_RS06810 | D | CCCGTCCC | -71 | -64 | CCGGTCCC | 0.88 |
| XC_RS06815 | D | CCCCGTCC | -39 | -32 | CTCCGTCC | 0.88 |
| XC_RS06850 | D | CTCCCGTC | -13 | -6 | CTCCCGAC | 0.88 |
| XC_RS06855 | D | CCCCTCTC | -66 | -59 | CACCTCTC | 0.88 |
| XC_RS06870 | R | CCCCGTCC | -179 | -172 | CCCCATCC | 0.88 |
| XC_RS06870 | D | CTCCCGTC | -106 | -99 | CTCGCGTC | 0.88 |
| XC_RS06875 | D | CCCCGTCC | -24 | -17 | CCCCATCC | 0.88 |
| XC_RS06875 | R | CTCCCGTC | -97 | -90 | CTCGCGTC | 0.88 |
| XC_RS06895 | D | CTCGCCGTC | -111 | -103 | CGCGCCGTC | 0.89 |
| XC_RS06900 | R | CCCGTCCC | -142 | -135 | CCCGACCC | 0.88 |
| XC_RS06900 | R | CCCGTCCC | -55 | -48 | TCCGTCCC | 0.88 |
| XC_RS06905 | D | CCCGTCCC | -146 | -139 | CCCGACCC | 0.88 |
| XC_RS06950 | D | CTCCCGTC | -17 | -10 | CTCCTGTC | 0.88 |
| XC_RS06960 | D | CCCCTCTC | -124 | -117 | CCACTCTC | 0.88 |
| XC_RS06960 | R | CCCCGTCC | -141 | -134 | CCGCGTCC | 0.88 |
| XC_RS06960 | D | CCTCTCTCCC | -124 | -115 | CCACTCTCCC | 0.9 |
| XC_RS06975 | R | CCCGTCCC | -143 | -136 | CACGTCCC | 0.88 |
| XC_RS06975 | R | CCCCTTCTCC | -109 | -100 | CTCCTTCTCC | 0.9 |
| XC_RS06980 | R | CTCCCGTC | -101 | -94 | CGCCCGTC | 0.88 |
| XC_RS06990 | R | CTCCCGTC | -41 | -34 | CTCCCGCC | 0.88 |
| XC_RS06995 | D | CTCCCGTC | -199 | -192 | CTCCCGCC | 0.88 |
| XC_RS07000 | R | CCCGTCCC | -30 | -23 | CCCGCCCC | 0.88 |
| XC_RS07010 | R | AGGCGGAGG | -51 | -43 | AGGCGGCGG | 0.89 |
| XC_RS07030 | D | CCCCTCTC | -20 | -13 | CCCCTGTC | 0.88 |
| XC_RS07030 | D | CCCCGTCC | -19 | -12 | CCCTGTCC | 0.88 |
| XC_RS07065 | R | CTCGCCGTC | -34 | -26 | CTCGCCGCC | 0.89 |
| XC_RS07090 | D | CTCCCGTC | -8 | -1 | CTCCCGTG | 0.88 |
| XC_RS07090 | D | AGGCGGAGG | -122 | -114 | AGGCCGAGG | 0.89 |
| XC_RS07095 | R | AGGCGGAGG | -113 | -105 | AGGCCGAGG | 0.89 |
| XC_RS07110 | D | CTCGCCGTC | -122 | -114 | TTCGCCGTC | 0.89 |
| XC_RS07110 | D | CTCGCCGTC | -97 | -89 | CGCGCCGTC | 0.89 |
| XC_RS07135 | R | AGGCGGAGG | -46 | -38 | ATGCGGAGG | 0.89 |
| XC_RS07140 | D | CTCCCGTC | -28 | -21 | CTCCCGCC | 0.88 |
| XC_RS07145 | D | CCCGTCCC | -20 | -13 | CCCGTTCC | 0.88 |
| XC_RS07145 | R | CCCGTCCC | -63 | -56 | CCCGGCCC | 0.88 |
| XC_RS07145 | D | CTCCCGTC | -22 | -15 | CTCCCGTT | 0.88 |
| XC_RS07185 | R | CTCCCGTC | -53 | -46 | CTCCCGGC | 0.88 |
| XC_RS07190 | D | CTCCCGTC | -157 | -150 | CTCCCGGC | 0.88 |
| XC_RS07200 | D | CCCCGTCC | -12 | -5 | GCCCGTCC | 0.88 |
| XC_RS07200 | D | CCCGTCCC | -11 | -4 | CCCGTCCC | 1 |
| XC_RS07240 | R | CCCCGTCC | -55 | -48 | ACCCGTCC | 0.88 |
| XC_RS07240 | R | CCCGTCCC | -56 | -49 | CCCGTCCG | 0.88 |
| XC_RS07240 | R | CTCCCGTC | -54 | -47 | CACCCGTC | 0.88 |
| XC_RS07275 | D | AGGCGGAGG | -116 | -108 | AGGCGGCGG | 0.89 |
| XC_RS07290 | D | CCCCGTCC | -47 | -40 | CCCCGTGC | 0.88 |
| XC_RS07290 | D | CCCGTCCC | -46 | -39 | CCCGTGCC | 0.88 |
| XC_RS07325 | R | CCCCCGCCTC | -15 | -6 | CCCCCGGCTC | 0.9 |
| XC_RS07330 | D | CTCCCGTC | -63 | -56 | CTCCCGCC | 0.88 |
| XC_RS07340 | D | CCCCGTCC | -96 | -89 | CCGCGTCC | 0.88 |
| XC_RS22550 | R | CCCCCGCCTC | -65 | -56 | CCCCCGCCAC | 0.9 |
| XC_RS07350 | D | CCCCTCTC | -26 | -19 | CCCCCCTC | 0.88 |
| XC_RS07350 | D | CCCCGTCC | -25 | -18 | CCCCCTCC | 0.88 |
| XC_RS07350 | D | CCCGTCCC | -24 | -17 | CCCCTCCC | 0.88 |
| XC_RS07350 | R | CCCGTCCC | -179 | -172 | CCCGTCGC | 0.88 |
| XC_RS07350 | R | CTCCCGTC | -177 | -170 | CGCCCGTC | 0.88 |
| XC_RS07360 | D | CTCCCGTC | -54 | -47 | CTCCCGGC | 0.88 |
| XC_RS07365 | R | CTCCCGTC | -126 | -119 | CTCCCGGC | 0.88 |
| XC_RS07370 | R | CCCCGTCC | -53 | -46 | CACCGTCC | 0.88 |
| XC_RS07390 | D | CCCCTCTC | -26 | -19 | CCCCCCTC | 0.88 |
| XC_RS07390 | D | CCCCCGCCTC | -28 | -19 | CCCCCCCCTC | 0.9 |
| XC_RS07395 | R | CCCCTCTC | -178 | -171 | CCCCTCGC | 0.88 |
| XC_RS07395 | R | CCCCCGCCTC | -176 | -167 | CCCCCCCCTC | 0.9 |
| XC_RS07410 | D | CCCCGTCC | -12 | -5 | CGCCGTCC | 0.88 |
| XC_RS07420 | R | CCCCGTCC | -167 | -160 | CCCCATCC | 0.88 |
| XC_RS07420 | R | CTCGCCGTC | -198 | -190 | CTCGCCGTG | 0.89 |
| XC_RS07425 | D | CCCCGTCC | -63 | -56 | CCCCATCC | 0.88 |
| XC_RS07425 | D | CTCGCCGTC | -33 | -25 | CTCGCCGTG | 0.89 |
| XC_RS07430 | D | AGGCGGAGG | -51 | -43 | ACGCGGAGG | 0.89 |
| XC_RS07440 | D | CCCCTCTC | -123 | -116 | CGCCTCTC | 0.88 |
| XC_RS07440 | R | CCCCGTCC | -108 | -101 | GCCCGTCC | 0.88 |
| XC_RS07440 | R | CCCGTCCC | -109 | -102 | CCCGTCCC | 1 |
| XC_RS07440 | R | CTCCCGTC | -107 | -100 | CGCCCGTC | 0.88 |
| XC_RS07440 | R | AGGCGGAGG | -127 | -119 | AGGCGGAAG | 0.89 |
| XC_RS07445 | R | CCCCTCTC | -88 | -81 | CCCATCTC | 0.88 |
| XC_RS07450 | D | CCCCTCTC | -90 | -83 | CCCATCTC | 0.88 |
| XC_RS07465 | R | CTCGCCGTC | -10 | -2 | CTCGACGTC | 0.89 |
| XC_RS07470 | R | CCCGTCCC | -55 | -48 | CCCGGCCC | 0.88 |
| XC_RS07475 | D | CCCGTCCC | -110 | -103 | CCCGGCCC | 0.88 |
| XC_RS07485 | D | CCCCTCTC | -82 | -75 | GCCCTCTC | 0.88 |
| XC_RS07485 | D | CCCCGTCC | -71 | -64 | CCCCGTTC | 0.88 |
| XC_RS07485 | D | CTCGCCGTC | -64 | -56 | CACGCCGTC | 0.89 |
| XC_RS07520 | D | CCCGTCCC | -103 | -96 | GCCGTCCC | 0.88 |
| XC_RS07525 | R | CCCGTCCC | -27 | -20 | GCCGTCCC | 0.88 |
| XC_RS07535 | R | CCCCGTCC | -60 | -53 | CCCCGTAC | 0.88 |
| XC_RS07535 | D | CTCCCGTC | -34 | -27 | CTCCCGAC | 0.88 |
| XC_RS07540 | R | CCCCGTCC | -91 | -84 | CCCCATCC | 0.88 |
| XC_RS07545 | D | CCCCGTCC | -15 | -8 | CCCCATCC | 0.88 |
| XC_RS22570 | D | CCCCGTCC | -22 | -15 | CCCCGACC | 0.88 |
| XC_RS07580 | R | CTCCCGTC | -17 | -10 | CGCCCGTC | 0.88 |
| XC_RS07605 | R | CCCCGTCC | -87 | -80 | CCGCGTCC | 0.88 |
| XC_RS07605 | R | CCCGTCCC | -88 | -81 | CGCGTCCC | 0.88 |
| XC_RS07630 | D | CCCGTCCC | -32 | -25 | CCCGTACC | 0.88 |
| XC_RS07660 | R | CTCCCGTC | -104 | -97 | CTCACGTC | 0.88 |
| XC_RS07710 | R | CCCCTCTC | -146 | -139 | CCCCTCGC | 0.88 |
| XC_RS07710 | R | CCCCGTCC | -16 | -9 | CCCCGTCA | 0.88 |
| XC_RS07710 | R | CCCGTCCC | -17 | -10 | CCCGTCAC | 0.88 |
| XC_RS22575 | D | CCCCTCTC | -122 | -115 | CCCCTCGC | 0.88 |
| XC_RS07715 | D | CCCCTTCTCC | -35 | -26 | CCACTTCTCC | 0.9 |
| XC_RS07740 | D | CTCCCGTC | -26 | -19 | CGCCCGTC | 0.88 |
| XC_RS07765 | D | CCCCGTCC | -25 | -18 | CCCCATCC | 0.88 |
| XC_RS07775 | D | CCCCTCTC | -150 | -143 | CCCCGCTC | 0.88 |
| XC_RS07775 | D | CCCCTCTC | -114 | -107 | TCCCTCTC | 0.88 |
| XC_RS07775 | D | CCCCGTCC | -119 | -112 | CCCTGTCC | 0.88 |
| XC_RS07775 | D | CCCGTCCC | -118 | -111 | CCTGTCCC | 0.88 |
| XC_RS07780 | D | CCCCGTCC | -64 | -57 | CCCCGGCC | 0.88 |
| XC_RS07780 | R | CTCGCCGTC | -55 | -47 | CCCGCCGTC | 0.89 |
| XC_RS07790 | D | AGGCGGAGG | -179 | -171 | AGGCGGCGG | 0.89 |
| XC_RS07790 | R | CTCGCCGTC | -38 | -30 | CTAGCCGTC | 0.89 |
| XC_RS07835 | D | CCCCTCTC | -23 | -16 | CCCCTTTC | 0.88 |
| XC_RS07840 | D | CCCCTCTC | -54 | -47 | CTCCTCTC | 0.88 |
| XC_RS07840 | D | CTCGCCGTC | -49 | -41 | CTCGCCATC | 0.89 |
| XC_RS07880 | D | CCCCTCTC | -47 | -40 | CCCCTCTG | 0.88 |
| XC_RS07915 | D | CTCCCGTC | -24 | -17 | CTCGCGTC | 0.88 |
| XC_RS07930 | R | CCCGTCCC | -15 | -8 | TCCGTCCC | 0.88 |
| XC_RS07950 | D | CTCCCGTC | -36 | -29 | CACCCGTC | 0.88 |
| XC_RS08005 | D | CTCGCCGTC | -33 | -25 | CTTGCCGTC | 0.89 |
| XC_RS08015 | D | CCCCGTCC | -139 | -132 | TCCCGTCC | 0.88 |
| XC_RS08015 | D | CCCGTCCC | -138 | -131 | CCCGTCCT | 0.88 |
| XC_RS08015 | D | CTCCCGTC | -140 | -133 | TTCCCGTC | 0.88 |
| XC_RS08035 | R | CTCCCGTC | -76 | -69 | CTGCCGTC | 0.88 |
| XC_RS08040 | R | CTCGCCGTC | -64 | -56 | CTCGCCGTT | 0.89 |
| XC_RS08045 | D | CTCCCGTC | -74 | -67 | CTCCCGGC | 0.88 |
| XC_RS08075 | D | CCCCTCTC | -37 | -30 | GCCCTCTC | 0.88 |
| XC_RS08075 | D | CTCCCGTC | -23 | -16 | CTCCCTTC | 0.88 |
| XC_RS08080 | R | CCCCTCTC | -92 | -85 | GCCCTCTC | 0.88 |
| XC_RS08080 | R | CTCCCGTC | -106 | -99 | CTCCCTTC | 0.88 |
| XC_RS08100 | D | CTCCCGTC | -66 | -59 | CTCCCGGC | 0.88 |
| XC_RS08100 | R | CTCCCGTC | -14 | -7 | CTCCTGTC | 0.88 |
| XC_RS08105 | R | CTCCCGTC | -107 | -100 | GTCCCGTC | 0.88 |
| XC_RS08125 | R | CCCGTCCC | -25 | -18 | CTCGTCCC | 0.88 |
| XC_RS08135 | R | CCCCTCTC | -10 | -3 | CGCCTCTC | 0.88 |
| XC_RS08140 | D | CCCCTCTC | -137 | -130 | CGCCTCTC | 0.88 |
| XC_RS08155 | R | CCCCTCTC | -11 | -4 | CCCTTCTC | 0.88 |
| XC_RS08155 | R | CCCCTTCTCC | -12 | -3 | GCCCTTCTCC | 0.9 |
| XC_RS08210 | D | CCCCTCTC | -191 | -184 | CCCCGCTC | 0.88 |
| XC_RS08210 | R | CCCGTCCC | -164 | -157 | CCCGACCC | 0.88 |
| XC_RS08215 | R | CCCCTCTC | -13 | -6 | CCCCTCCC | 0.88 |
| XC_RS08215 | R | CCCGTCCC | -13 | -6 | CCCCTCCC | 0.88 |
| XC_RS08220 | R | CCCCTCTC | -145 | -138 | ACCCTCTC | 0.88 |
| XC_RS08225 | D | CCCCGTCC | -17 | -10 | CGCCGTCC | 0.88 |
| XC_RS08225 | D | CTCGCCGTC | -19 | -11 | ATCGCCGTC | 0.89 |
| XC_RS08235 | D | CCCCTCTC | -157 | -150 | CCCCTCCC | 0.88 |
| XC_RS08235 | R | CCCCTCTC | -10 | -3 | CCCCTCCC | 0.88 |
| XC_RS08235 | D | CCCGTCCC | -157 | -150 | CCCCTCCC | 0.88 |
| XC_RS08235 | R | CCCGTCCC | -10 | -3 | CCCCTCCC | 0.88 |
| XC_RS08240 | R | CCCCTCTC | -93 | -86 | CCCCTCCC | 0.88 |
| XC_RS08240 | R | CCCGTCCC | -93 | -86 | CCCCTCCC | 0.88 |
| XC_RS08240 | R | CCTCTCTCCC | -39 | -30 | CCTCCCTCCC | 0.9 |
| XC_RS08260 | D | CCCGTCCC | -73 | -66 | CCTGTCCC | 0.88 |
| XC_RS08265 | D | CCCCGTCC | -43 | -36 | CCCCGGCC | 0.88 |
| XC_RS08280 | R | CCCGTCCC | -138 | -131 | CCCTTCCC | 0.88 |
| XC_RS08305 | D | CCCGTCCC | -92 | -85 | CCTGTCCC | 0.88 |
| XC_RS08305 | R | AGGCGGAGG | -25 | -17 | AGGCTGAGG | 0.89 |
| XC_RS08375 | D | CCCCGTCC | -132 | -125 | CCCCATCC | 0.88 |
| XC_RS08375 | D | CCCGTCCC | -131 | -124 | CCCATCCC | 0.88 |
| XC_RS08375 | D | CTCGCCGTC | -102 | -94 | CTCGCCGTG | 0.89 |
| XC_RS08440 | R | CTCCCGTC | -15 | -8 | CTCCTGTC | 0.88 |
| XC_RS08445 | D | CCCGTCCC | -66 | -59 | CCCGTCGC | 0.88 |
| XC_RS08445 | D | CTCCCGTC | -21 | -14 | CTTCCGTC | 0.88 |
| XC_RS08445 | R | AGGCGGAGG | -17 | -9 | AGGCGGACG | 0.89 |
| XC_RS08450 | R | CCCGTCCC | -16 | -9 | CCCGTCGC | 0.88 |
| XC_RS08450 | R | CTCCCGTC | -61 | -54 | CTTCCGTC | 0.88 |
| XC_RS08450 | D | AGGCGGAGG | -66 | -58 | AGGCGGACG | 0.89 |
| XC_RS08470 | D | CTCCCGTC | -40 | -33 | CTGCCGTC | 0.88 |
| XC_RS08490 | R | AGGCGGAGG | -28 | -20 | AGGCGAAGG | 0.89 |
| XC_RS08500 | R | CCCGTCCC | -46 | -39 | CCCGACCC | 0.88 |
| XC_RS08500 | D | CTCCCGTC | -152 | -145 | CTCCCATC | 0.88 |
| XC_RS08505 | D | CCCGTCCC | -123 | -116 | CCCGACCC | 0.88 |
| XC_RS08505 | R | CTCCCGTC | -17 | -10 | CTCCCATC | 0.88 |
| XC_RS08540 | R | CCCCGTCC | -102 | -95 | CACCGTCC | 0.88 |
| XC_RS08565 | R | CCCCGTCC | -94 | -87 | ACCCGTCC | 0.88 |
| XC_RS08565 | R | CCCGTCCC | -95 | -88 | CCCGTCCA | 0.88 |
| XC_RS08565 | R | CTCCCGTC | -93 | -86 | CACCCGTC | 0.88 |
| XC_RS08570 | D | CCCCGTCC | -66 | -59 | CCGCGTCC | 0.88 |
| XC_RS08585 | D | CCCCGTCC | -169 | -162 | CCACGTCC | 0.88 |
| XC_RS08615 | D | CTCCCGTC | -71 | -64 | CTCCCGAC | 0.88 |
| XC_RS08680 | D | CCCCTCTC | -20 | -13 | CCCCACTC | 0.88 |
| XC_RS08685 | R | CCCGTCCC | -33 | -26 | CCCGTCAC | 0.88 |
| XC_RS08685 | R | CTCCCGTC | -31 | -24 | TTCCCGTC | 0.88 |
| XC_RS08690 | D | CCCGTCCC | -86 | -79 | CCCGTCAC | 0.88 |
| XC_RS08690 | D | CTCCCGTC | -88 | -81 | TTCCCGTC | 0.88 |
| XC_RS08695 | D | CCCGTCCC | -164 | -157 | CCCGTACC | 0.88 |
| XC_RS08695 | R | CTCCCGTC | -126 | -119 | CTCCCATC | 0.88 |
| XC_RS08695 | D | CCCCTTCTCC | -137 | -128 | CACCTTCTCC | 0.9 |
| XC_RS08700 | D | CCCCTTCTCC | -54 | -45 | CCGCTTCTCC | 0.9 |
| XC_RS08705 | R | CCCCGTCC | -108 | -101 | CGCCGTCC | 0.88 |
| XC_RS08705 | D | CTCCCGTC | -129 | -122 | CTGCCGTC | 0.88 |
| XC_RS08705 | R | CTCGCCGTC | -107 | -99 | CACGCCGTC | 0.89 |
| XC_RS08730 | D | CCCCTCTC | -74 | -67 | CCCCGCTC | 0.88 |
| XC_RS08730 | D | CCCCGTCC | -117 | -110 | GCCCGTCC | 0.88 |
| XC_RS08730 | D | CCCGTCCC | -116 | -109 | CCCGTCCC | 1 |
| XC_RS22610 | D | CCCCGTCC | -30 | -23 | ACCCGTCC | 0.88 |
| XC_RS22610 | D | CCCGTCCC | -29 | -22 | CCCGTCCC | 1 |
| XC_RS08765 | R | CTCCCGTC | -55 | -48 | CGCCCGTC | 0.88 |
| XC_RS08775 | R | CTCGCCGTC | -59 | -51 | GTCGCCGTC | 0.89 |
| XC_RS08780 | D | CTCGCCGTC | -158 | -150 | GTCGCCGTC | 0.89 |
| XC_RS08790 | D | CCCCGTCC | -27 | -20 | CCCCGTAC | 0.88 |
| XC_RS08790 | D | CCCGTCCC | -26 | -19 | CCCGTACC | 0.88 |
| XC_RS08800 | R | CCCCGTCC | -175 | -168 | CCCCGTAC | 0.88 |
| XC_RS08800 | R | CCCGTCCC | -176 | -169 | CCCGTACC | 0.88 |
| XC_RS08810 | D | CCCCGTCC | -19 | -12 | CCCCGCCC | 0.88 |
| XC_RS08810 | D | CCCGTCCC | -24 | -17 | CCCATCCC | 0.88 |
| XC_RS08835 | D | AGGCGGAGG | -72 | -64 | TGGCGGAGG | 0.89 |
| XC_RS08835 | D | CTCGCCGTC | -180 | -172 | CTCGCCTTC | 0.89 |
| XC_RS08855 | R | AGGCGGAGG | -58 | -50 | AGGCGCAGG | 0.89 |
| XC_RS08860 | D | AGGCGGAGG | -80 | -72 | AGGCGCAGG | 0.89 |
| XC_RS08875 | D | CCCGTCCC | -143 | -136 | GCCGTCCC | 0.88 |
| XC_RS08910 | D | CCCGTCCC | -24 | -17 | CCCGGCCC | 0.88 |
| XC_RS22630 | D | CTCCCGTC | -97 | -90 | CTCCCGGC | 0.88 |
| XC_RS08930 | D | CCCCGTCC | -10 | -3 | CCCCGTCA | 0.88 |
| XC_RS08940 | D | CTCCCGTC | -31 | -24 | CTCCTGTC | 0.88 |
| XC_RS08945 | R | CTCCCGTC | -79 | -72 | CTACCGTC | 0.88 |
| XC_RS08950 | D | CCCCGTCC | -41 | -34 | TCCCGTCC | 0.88 |
| XC_RS08950 | D | CCCGTCCC | -40 | -33 | CCCGTCCT | 0.88 |
| XC_RS08950 | D | CTCCCGTC | -42 | -35 | CTCCCGTC | 1 |
| XC_RS08960 | D | CTCGCCGTC | -29 | -21 | CTCGCCTTC | 0.89 |
| XC_RS08965 | R | CTCGCCGTC | -64 | -56 | CTCGCCTTC | 0.89 |
| XC_RS08975 | R | CCCCTCTC | -148 | -141 | ACCCTCTC | 0.88 |
| XC_RS09005 | R | CTCGCCGTC | -78 | -70 | CTCGCAGTC | 0.89 |
| XC_RS09010 | D | CCCCTCTC | -56 | -49 | CCCCTGTC | 0.88 |
| XC_RS09010 | D | CCCCGTCC | -8 | -1 | CCCCGCCC | 0.88 |
| XC_RS09030 | D | CTCGCCGTC | -58 | -50 | CGCGCCGTC | 0.89 |
| XC_RS09035 | D | CCCCGTCC | -48 | -41 | CGCCGTCC | 0.88 |
| XC_RS09085 | D | CCCCGTCC | -73 | -66 | CACCGTCC | 0.88 |
| XC_RS09115 | R | CCCCTCTC | -21 | -14 | CCCCTCCC | 0.88 |
| XC_RS09115 | R | CCCGTCCC | -21 | -14 | CCCCTCCC | 0.88 |
| XC_RS09115 | D | AGGCGGAGG | -24 | -16 | AGGGGGAGG | 0.89 |
| XC_RS09125 | D | CCCCGTCC | -94 | -87 | CCCCGTCT | 0.88 |
| XC_RS09135 | R | CCCCGTCC | -52 | -45 | CCACGTCC | 0.88 |
| XC_RS09140 | D | CCCCGTCC | -56 | -49 | CCACGTCC | 0.88 |
| XC_RS09145 | D | CCCCTCTC | -21 | -14 | CCCGTCTC | 0.88 |
| XC_RS09145 | D | CCCCGTCC | -67 | -60 | GCCCGTCC | 0.88 |
| XC_RS09145 | D | CCCGTCCC | -66 | -59 | CCCGTCCA | 0.88 |
| XC_RS09145 | D | CCCGTCCC | -21 | -14 | CCCGTCTC | 0.88 |
| XC_RS09145 | D | CTCCCGTC | -68 | -61 | CGCCCGTC | 0.88 |
| XC_RS09145 | D | CTCGCCGTC | -83 | -75 | CTCACCGTC | 0.89 |
| XC_RS09160 | D | CCCCGTCC | -33 | -26 | CCCCGTCA | 0.88 |
| XC_RS09160 | D | CTCCCGTC | -34 | -27 | CCCCCGTC | 0.88 |
| XC_RS09175 | D | CCCCTCTC | -33 | -26 | GCCCTCTC | 0.88 |
| XC_RS09180 | R | CCCCTCTC | -144 | -137 | GCCCTCTC | 0.88 |
| XC_RS09190 | D | CTCGCCGTC | -52 | -44 | CTCGCCGTT | 0.89 |
| XC_RS09195 | R | CTCGCCGTC | -57 | -49 | CTCGCCGTT | 0.89 |
| XC_RS09205 | D | CCCGTCCC | -102 | -95 | CCCTTCCC | 0.88 |
| XC_RS09210 | R | CCCGTCCC | -192 | -185 | CCCTTCCC | 0.88 |
| XC_RS09210 | D | CCCCCGCCTC | -94 | -85 | CCCTCGCCTC | 0.9 |
| XC_RS09215 | R | CTCCCGTC | -16 | -9 | CTCCCGTG | 0.88 |
| XC_RS09220 | D | CTCCCGTC | -108 | -101 | CTCCCGTG | 0.88 |
| XC_RS09260 | R | CCCCTCTC | -9 | -2 | CCCCGCTC | 0.88 |
| XC_RS09270 | D | CCCCTCTC | -36 | -29 | CACCTCTC | 0.88 |
| XC_RS09270 | D | CCTCTCTCCC | -34 | -25 | CCTCTCTCCC | 1 |
| XC_RS09305 | D | CCCCTCTC | -21 | -14 | CCCCTCCC | 0.88 |
| XC_RS09305 | D | CCCCGTCC | -15 | -8 | CCCCGACC | 0.88 |
| XC_RS09305 | D | CCCGTCCC | -21 | -14 | CCCCTCCC | 0.88 |
| XC_RS09305 | D | CTCCCGTC | -23 | -16 | CTCCCCTC | 0.88 |
| XC_RS09355 | D | AGGCGGAGG | -138 | -130 | AGGCGGAGC | 0.89 |
| XC_RS09360 | R | AGGCGGAGG | -20 | -12 | AGGCGGAGC | 0.89 |
| XC_RS09380 | D | CCCGTCCC | -143 | -136 | CCCGTACC | 0.88 |
| XC_RS09380 | R | CCCGTCCC | -186 | -179 | CCCGTACC | 0.88 |
| XC_RS09380 | D | CCCCTTCTCC | -116 | -107 | CACCTTCTCC | 0.9 |
| XC_RS09400 | D | CCCGTCCC | -73 | -66 | CGCGTCCC | 0.88 |
| XC_RS09420 | D | CCCCTCTC | -28 | -21 | CCCCACTC | 0.88 |
| XC_RS09470 | D | CTCCCGTC | -28 | -21 | CTCCCGCC | 0.88 |
| XC_RS09515 | D | CCCCGTCC | -50 | -43 | CCCTGTCC | 0.88 |
| XC_RS09515 | D | CCCGTCCC | -49 | -42 | CCTGTCCC | 0.88 |
| XC_RS09515 | D | CCCGTCCC | -40 | -33 | CCCTTCCC | 0.88 |
| XC_RS09515 | R | CCCGTCCC | -22 | -15 | CCCGTCGC | 0.88 |
| XC_RS09515 | R | CTCCCGTC | -20 | -13 | CTCCCGTC | 1 |
| XC_RS09515 | R | CTCGCCGTC | -171 | -163 | CTCGCGGTC | 0.89 |
| XC_RS09520 | R | CCCCGTCC | -155 | -148 | CCCTGTCC | 0.88 |
| XC_RS09520 | D | CCCGTCCC | -183 | -176 | CCCGTCGC | 0.88 |
| XC_RS09520 | R | CCCGTCCC | -165 | -158 | CCCTTCCC | 0.88 |
| XC_RS09520 | R | CCCGTCCC | -156 | -149 | CCTGTCCC | 0.88 |
| XC_RS09520 | D | CTCCCGTC | -185 | -178 | CTCCCGTC | 1 |
| XC_RS09520 | D | CTCGCCGTC | -35 | -27 | CTCGCGGTC | 0.89 |
| XC_RS09525 | D | CCCCTCTC | -33 | -26 | CCCCACTC | 0.88 |
| XC_RS09525 | D | CCCGTCCC | -39 | -32 | CCCGTTCC | 0.88 |
| XC_RS09535 | D | CCCCGTCC | -25 | -18 | CCACGTCC | 0.88 |
| XC_RS09535 | R | CCCGTCCC | -153 | -146 | CCCGTGCC | 0.88 |
| XC_RS09575 | R | CCCCTCTC | -13 | -6 | CCCCACTC | 0.88 |
| XC_RS09600 | D | CCCCGTCC | -98 | -91 | CCCTGTCC | 0.88 |
| XC_RS09620 | D | CCCCGTCC | -189 | -182 | CCACGTCC | 0.88 |
| XC_RS09640 | D | CCCGTCCC | -167 | -160 | CACGTCCC | 0.88 |
| XC_RS09720 | R | CCCCGTCC | -177 | -170 | CCGCGTCC | 0.88 |
| XC_RS09725 | R | CCCCTCTC | -86 | -79 | CCCCTCTT | 0.88 |
| XC_RS09755 | D | CCCCTCTC | -22 | -15 | CACCTCTC | 0.88 |
| XC_RS09755 | D | CCCGTCCC | -29 | -22 | CCCATCCC | 0.88 |
| XC_RS09760 | D | CCCCGTCC | -25 | -18 | CCGCGTCC | 0.88 |
| XC_RS09760 | D | CCCGTCCC | -24 | -17 | CGCGTCCC | 0.88 |
| XC_RS09760 | D | CTCCCGTC | -21 | -14 | GTCCCGTC | 0.88 |
| XC_RS09765 | R | CCCCGTCC | -126 | -119 | CCGCGTCC | 0.88 |
| XC_RS09765 | R | CCCGTCCC | -127 | -120 | CGCGTCCC | 0.88 |
| XC_RS09765 | R | CTCCCGTC | -130 | -123 | GTCCCGTC | 0.88 |
| XC_RS09775 | R | CCCCGTCC | -10 | -3 | CACCGTCC | 0.88 |
| XC_RS09795 | R | CCCCGTCC | -88 | -81 | CCCCGCCC | 0.88 |
| XC_RS09795 | R | CTCCCGTC | -189 | -182 | CTCCCGCC | 0.88 |
| XC_RS09815 | R | CTCCCGTC | -14 | -7 | CTCCCGTG | 0.88 |
| XC_RS09835 | R | CTCGCCGTC | -159 | -151 | CGCGCCGTC | 0.89 |
| XC_RS09840 | D | CTCGCCGTC | -42 | -34 | CGCGCCGTC | 0.89 |
| XC_RS09865 | D | CCCCTCTC | -25 | -18 | CCTCTCTC | 0.88 |
| XC_RS09900 | D | CCCCTCTC | -12 | -5 | CCACTCTC | 0.88 |
| XC_RS22655 | D | CCCCTCTC | -66 | -59 | CCGCTCTC | 0.88 |
| XC_RS22655 | D | CCCCGTCC | -51 | -44 | CCCCGGCC | 0.88 |
| XC_RS22655 | D | CTCGCCGTC | -91 | -83 | CTCGCCGCC | 0.89 |
| XC_RS09915 | D | CCCCTCTC | -46 | -39 | TCCCTCTC | 0.88 |
| XC_RS09915 | D | CCCCGTCC | -51 | -44 | CCCCTTCC | 0.88 |
| XC_RS09915 | D | CCCGTCCC | -50 | -43 | CCCTTCCC | 0.88 |
| XC_RS09915 | D | CTCGCCGTC | -22 | -14 | GTCGCCGTC | 0.89 |
| XC_RS09940 | R | CCCCGTCC | -180 | -173 | CGCCGTCC | 0.88 |
| XC_RS09940 | R | CTCGCCGTC | -179 | -171 | CACGCCGTC | 0.89 |
| XC_RS09980 | D | CCCCTCTC | -25 | -18 | CCCCTCGC | 0.88 |
| XC_RS10080 | D | CCCCCGCCTC | -174 | -165 | CCCCCGCTTC | 0.9 |
| XC_RS10145 | D | CCCGTCCC | -56 | -49 | CCCGTGCC | 0.88 |
| XC_RS10170 | R | CCCCTCTC | -51 | -44 | CCCCTGTC | 0.88 |
| XC_RS10245 | R | CCCGTCCC | -10 | -3 | CCCGCCCC | 0.88 |
| XC_RS10295 | R | CTCGCCGTC | -11 | -3 | CTCGGCGTC | 0.89 |
| XC_RS10320 | D | CCCCTCTC | -24 | -17 | CCCCGCTC | 0.88 |
| XC_RS22705 | D | CTCCCGTC | -60 | -53 | CACCCGTC | 0.88 |
| XC_RS10325 | D | CCCCTCTC | -51 | -44 | CCCCTCAC | 0.88 |
| XC_RS10330 | R | AGGCGGAGG | -27 | -19 | AAGCGGAGG | 0.89 |
| XC_RS10350 | D | CCCCGTCC | -81 | -74 | CCGCGTCC | 0.88 |
| XC_RS10350 | R | CCCCGTCC | -40 | -33 | CCCCGCCC | 0.88 |
| XC_RS10375 | R | CCCCGTCC | -33 | -26 | CCCTGTCC | 0.88 |
| XC_RS10375 | R | CCCGTCCC | -34 | -27 | CCTGTCCC | 0.88 |
| XC_RS10380 | D | CCCGTCCC | -45 | -38 | CCCGGCCC | 0.88 |
| XC_RS23640 | R | CCCGTCCC | -88 | -81 | CCCGGCCC | 0.88 |
| XC_RS10390 | R | CCCGTCCC | -35 | -28 | CCCGTGCC | 0.88 |
| XC_RS10410 | R | CTCGCCGTC | -10 | -2 | CTCGCCTTC | 0.89 |
| XC_RS10425 | D | CCCCGTCC | -25 | -18 | CCCCATCC | 0.88 |
| XC_RS10425 | D | CTCCCGTC | -42 | -35 | CTCCCCTC | 0.88 |
| XC_RS10425 | R | CTCGCCGTC | -13 | -5 | CTCTCCGTC | 0.89 |
| XC_RS23645 | D | CCCGTCCC | -169 | -162 | CCCGTCGC | 0.88 |
| XC_RS10440 | D | CCCGTCCC | -22 | -15 | CCTGTCCC | 0.88 |
| XC_RS10440 | D | CTCCCGTC | -107 | -100 | CTACCGTC | 0.88 |
| XC_RS10450 | R | CCCCTCTC | -15 | -8 | CCGCTCTC | 0.88 |
| XC_RS10505 | D | CCCGTCCC | -85 | -78 | CCCATCCC | 0.88 |
| XC_RS10510 | R | CTCGCCGTC | -61 | -53 | CTCACCGTC | 0.89 |
| XC_RS10560 | D | CCCGTCCC | -61 | -54 | CCCGTTCC | 0.88 |
| XC_RS10580 | D | CCCCTCTC | -66 | -59 | CCCCGCTC | 0.88 |
| XC_RS10585 | R | CCCCTCTC | -34 | -27 | CCCCGCTC | 0.88 |
| XC_RS10590 | R | CCCCGTCC | -32 | -25 | CCCGGTCC | 0.88 |
| XC_RS10600 | D | CCCCTCTC | -121 | -114 | CCCCTATC | 0.88 |
| XC_RS10600 | R | CTCCCGTC | -89 | -82 | CTCCCCTC | 0.88 |
| XC_RS10600 | R | CCCCCGCCTC | -84 | -75 | CCCCCGGCTC | 0.9 |
| XC_RS10605 | R | CCCCTCTC | -172 | -165 | CCCCTATC | 0.88 |
| XC_RS22755 | D | CCCCTCTC | -64 | -57 | GCCCTCTC | 0.88 |
| XC_RS22770 | D | CCCCTCTC | -64 | -57 | GCCCTCTC | 0.88 |
| XC_RS10700 | R | CCCCTCTC | -12 | -5 | CCCCTATC | 0.88 |
| XC_RS10700 | D | CTCCCGTC | -84 | -77 | CTCCCGTG | 0.88 |
| XC_RS10700 | D | CTCCCGTC | -44 | -37 | CTCCCCTC | 0.88 |
| XC_RS10700 | D | CCCCCGCCTC | -51 | -42 | CCCCCGGCTC | 0.9 |
| XC_RS10705 | D | CCCCTCTC | -81 | -74 | CCCCTATC | 0.88 |
| XC_RS10705 | R | CTCCCGTC | -49 | -42 | CTCCCCTC | 0.88 |
| XC_RS10705 | R | CTCCCGTC | -9 | -2 | CTCCCGTG | 0.88 |
| XC_RS10705 | R | CCCCCGCCTC | -44 | -35 | CCCCCGGCTC | 0.9 |
| XC_RS10715 | R | CCCCTCTC | -198 | -191 | CCCCTTTC | 0.88 |
| XC_RS10735 | D | CCCCGTCC | -158 | -151 | CCCCGTTC | 0.88 |
| XC_RS22790 | D | CCCGTCCC | -189 | -182 | CCCGTTCC | 0.88 |
| XC_RS10750 | D | CTCCCGTC | -154 | -147 | CTCCCGGC | 0.88 |
| XC_RS10755 | R | CCCGTCCC | -140 | -133 | CCCGTCGC | 0.88 |
| XC_RS10755 | D | CTCCCGTC | -44 | -37 | CTCCCGTG | 0.88 |
| XC_RS10765 | D | CCCGTCCC | -16 | -9 | CCCTTCCC | 0.88 |
| XC_RS10775 | D | CCCGTCCC | -72 | -65 | CCCGGCCC | 0.88 |
| XC_RS10790 | R | CCCCGTCC | -11 | -4 | CGCCGTCC | 0.88 |
| XC_RS10795 | D | CCCCGTCC | -158 | -151 | CGCCGTCC | 0.88 |
| XC_RS10825 | D | CTCGCCGTC | -76 | -68 | CTCGACGTC | 0.89 |
| XC_RS22800 | R | CTCGCCGTC | -170 | -162 | CTCGACGTC | 0.89 |
| XC_RS10845 | D | CCCCGTCC | -161 | -154 | CCCCGGCC | 0.88 |
| XC_RS10845 | D | CCCCGTCC | -136 | -129 | CTCCGTCC | 0.88 |
| XC_RS10855 | R | CCCGTCCC | -58 | -51 | CCCATCCC | 0.88 |
| XC_RS10855 | D | CTCCCGTC | -98 | -91 | CTCCCGCC | 0.88 |
| XC_RS10855 | R | CTCCCGTC | -61 | -54 | ATCCCGTC | 0.88 |
| XC_RS10875 | D | CCCCTCTC | -173 | -166 | GCCCTCTC | 0.88 |
| XC_RS10890 | R | AGGCGGAGG | -93 | -85 | AGGCGAAGG | 0.89 |
| XC_RS10895 | D | CTCGCCGTC | -117 | -109 | CTCGCCATC | 0.89 |
| XC_RS10905 | R | CCCCTCTC | -13 | -6 | CTCCTCTC | 0.88 |
| XC_RS10905 | R | CCCGTCCC | -169 | -162 | CCCGACCC | 0.88 |
| XC_RS10980 | D | CCCCGTCC | -25 | -18 | CCACGTCC | 0.88 |
| XC_RS10980 | D | CTCGCCGTC | -13 | -5 | CTCGCCGCC | 0.89 |
| XC_RS10985 | R | CCCCGTCC | -67 | -60 | CCACGTCC | 0.88 |
| XC_RS10985 | R | CTCGCCGTC | -80 | -72 | CTCGCCGCC | 0.89 |
| XC_RS11000 | R | CCCCTCTC | -161 | -154 | CCCCTCAC | 0.88 |
| XC_RS11000 | R | CCCCGTCC | -155 | -148 | CCGCGTCC | 0.88 |
| XC_RS11000 | R | CCCCGTCC | -126 | -119 | CCCCGCCC | 0.88 |
| XC_RS11000 | R | CCCGTCCC | -156 | -149 | CGCGTCCC | 0.88 |
| XC_RS11005 | D | CCCCTCTC | -19 | -12 | CCCCTCAC | 0.88 |
| XC_RS11005 | D | CCCCGTCC | -54 | -47 | CCCCGCCC | 0.88 |
| XC_RS11005 | D | CCCCGTCC | -25 | -18 | CCGCGTCC | 0.88 |
| XC_RS11005 | D | CCCGTCCC | -24 | -17 | CGCGTCCC | 0.88 |
| XC_RS11010 | R | CCCCTCTC | -119 | -112 | CCCCTCGC | 0.88 |
| XC_RS11010 | R | CCCGTCCC | -171 | -164 | ACCGTCCC | 0.88 |
| XC_RS11015 | D | CCCCTCTC | -62 | -55 | CCCCTCGC | 0.88 |
| XC_RS11015 | D | CCCGTCCC | -10 | -3 | ACCGTCCC | 0.88 |
| XC_RS11095 | R | CTCGCCGTC | -111 | -103 | CTCGTCGTC | 0.89 |
| XC_RS11100 | D | CTCGCCGTC | -116 | -108 | CTCGTCGTC | 0.89 |
| XC_RS11110 | R | CCCCGTCC | -81 | -74 | TCCCGTCC | 0.88 |
| XC_RS11110 | R | CCCGTCCC | -82 | -75 | CCCGTCCG | 0.88 |
| XC_RS11110 | R | CTCCCGTC | -80 | -73 | GTCCCGTC | 0.88 |
| XC_RS11150 | R | CCCCTCTC | -15 | -8 | CCCCTCTA | 0.88 |
| XC_RS11170 | D | AGGCGGAGG | -32 | -24 | ATGCGGAGG | 0.89 |
| XC_RS11175 | R | CCCCGTCC | -68 | -61 | CGCCGTCC | 0.88 |
| XC_RS11210 | D | CCCCCGCCTC | -135 | -126 | CCCCTGCCTC | 0.9 |
| XC_RS11220 | D | CCCCGTCC | -9 | -2 | CCTCGTCC | 0.88 |
| XC_RS11220 | D | CCCGTCCC | -8 | -1 | CTCGTCCC | 0.88 |
| XC_RS11235 | D | CCCCTCTC | -73 | -66 | CCCCACTC | 0.88 |
| XC_RS11235 | D | CCCGTCCC | -153 | -146 | TCCGTCCC | 0.88 |
| XC_RS11235 | R | CTCCCGTC | -134 | -127 | CGCCCGTC | 0.88 |
| XC_RS11240 | D | CCCCTCTC | -20 | -13 | CCCCTGTC | 0.88 |
| XC_RS11240 | R | CCCCTCTC | -177 | -170 | CCCCACTC | 0.88 |
| XC_RS11240 | R | CCCGTCCC | -97 | -90 | TCCGTCCC | 0.88 |
| XC_RS11240 | D | CTCCCGTC | -116 | -109 | CGCCCGTC | 0.88 |
| XC_RS11245 | D | CCCCTCTC | -181 | -174 | CCCGTCTC | 0.88 |
| XC_RS11245 | D | CCCCGTCC | -182 | -175 | CCCCGTCT | 0.88 |
| XC_RS11245 | D | CCCGTCCC | -181 | -174 | CCCGTCTC | 0.88 |
| XC_RS11245 | D | CCCGTCCC | -78 | -71 | CCCGGCCC | 0.88 |
| XC_RS11245 | R | CTCCCGTC | -14 | -7 | CTCCCGTG | 0.88 |
| XC_RS11245 | D | CCCCTTCTCC | -182 | -173 | CCCCGTCTCC | 0.9 |
| XC_RS11265 | D | CCCCTCTC | -70 | -63 | CCCTTCTC | 0.88 |
| XC_RS11265 | D | CCCCTTCTCC | -71 | -62 | CCCCTTCTCC | 1 |
| XC_RS11270 | R | AGGCGGAGG | -173 | -165 | AGGCGGAAG | 0.89 |
| XC_RS11275 | D | CCCCTCTC | -21 | -14 | GCCCTCTC | 0.88 |
| XC_RS11305 | D | CCCCTCTC | -194 | -187 | CCCCTCTC | 1 |
| XC_RS11305 | D | CCCCTCTC | -55 | -48 | CCCGTCTC | 0.88 |
| XC_RS11305 | D | CCCGTCCC | -55 | -48 | CCCGTCTC | 0.88 |
| XC_RS11330 | D | CCCGTCCC | -69 | -62 | CCCGTCGC | 0.88 |
| XC_RS11405 | D | CCCCGTCC | -142 | -135 | CCCCGCCC | 0.88 |
| XC_RS11405 | R | CTCCCGTC | -76 | -69 | CGCCCGTC | 0.88 |
| XC_RS11410 | D | CCCCGTCC | -10 | -3 | CACCGTCC | 0.88 |
| XC_RS11410 | D | CCCGTCCC | -9 | -2 | ACCGTCCC | 0.88 |
| XC_RS11445 | D | CTCCCGTC | -19 | -12 | CTTCCGTC | 0.88 |
| XC_RS11455 | D | CCCCGTCC | -86 | -79 | CCCCGTCG | 0.88 |
| XC_RS11455 | D | CCCGTCCC | -85 | -78 | CCCGTCGC | 0.88 |
| XC_RS11460 | D | CCCCGTCC | -95 | -88 | CCCCGCCC | 0.88 |
| XC_RS11460 | D | CCCGTCCC | -129 | -122 | CCCGCCCC | 0.88 |
| XC_RS11470 | R | CCCGTCCC | -122 | -115 | CCCGCCCC | 0.88 |
| XC_RS11470 | R | CCCGTCCC | -91 | -84 | CCTGTCCC | 0.88 |
| XC_RS11550 | D | CCCCGTCC | -169 | -162 | CCCCGTCA | 0.88 |
| XC_RS11555 | R | CTCCCGTC | -200 | -193 | CTCCCCTC | 0.88 |
| XC_RS22840 | D | CCCCTCTC | -162 | -155 | CCCGTCTC | 0.88 |
| XC_RS22840 | D | CCCCTCTC | -57 | -50 | CCCCTCTC | 1 |
| XC_RS22840 | R | CCCCTCTC | -184 | -177 | CCGCTCTC | 0.88 |
| XC_RS22840 | R | CCCCTCTC | -42 | -35 | CCCCTCTC | 1 |
| XC_RS22840 | D | CCCGTCCC | -162 | -155 | CCCGTCTC | 0.88 |
| XC_RS22840 | D | CCTCTCTCCC | -57 | -48 | CCCCTCTCCC | 0.9 |
| XC_RS22840 | R | CCTCTCTCCC | -44 | -35 | CCCCTCTCCC | 0.9 |
| XC_RS11560 | D | CCCCTCTC | -112 | -105 | CCGCTCTC | 0.88 |
| XC_RS11560 | R | CCCCTCTC | -134 | -127 | CCCGTCTC | 0.88 |
| XC_RS11560 | R | CCCGTCCC | -134 | -127 | CCCGTCTC | 0.88 |
| XC_RS11595 | D | CCCGTCCC | -21 | -14 | CCCTTCCC | 0.88 |
| XC_RS11615 | D | CCCCGTCC | -126 | -119 | CACCGTCC | 0.88 |
| XC_RS11615 | D | CCCGTCCC | -125 | -118 | ACCGTCCC | 0.88 |
| XC_RS11615 | R | CCCGTCCC | -56 | -49 | CCCGTGCC | 0.88 |
| XC_RS11615 | D | CTCGCCGTC | -128 | -120 | CTCACCGTC | 0.89 |
| XC_RS11615 | D | CCCCTTCTCC | -168 | -159 | CCCCTGCTCC | 0.9 |
| XC_RS11620 | D | AGGCGGAGG | -95 | -87 | AGGCGGAGG | 1 |
| XC_RS11630 | D | CCCCGTCC | -98 | -91 | CCCCGGCC | 0.88 |
| XC_RS11630 | D | CTCCCGTC | -32 | -25 | CTACCGTC | 0.88 |
| XC_RS22860 | D | CCCCTCTC | -27 | -20 | CCCCTCCC | 0.88 |
| XC_RS22860 | D | CCCGTCCC | -27 | -20 | CCCCTCCC | 0.88 |
| XC_RS11655 | D | CCCCGTCC | -64 | -57 | CCACGTCC | 0.88 |
| XC_RS11655 | R | CCCCGTCC | -54 | -47 | CCACGTCC | 0.88 |
| XC_RS11655 | D | CCCGTCCC | -63 | -56 | CACGTCCC | 0.88 |
| XC_RS11660 | D | CTCCCGTC | -87 | -80 | CGCCCGTC | 0.88 |
| XC_RS11675 | D | CCCCCGCCTC | -90 | -81 | CCCCCGCATC | 0.9 |
| XC_RS11705 | D | CCCCTCTC | -27 | -20 | CCCCCCTC | 0.88 |
| XC_RS11705 | D | CCCCGTCC | -26 | -19 | CCCCCTCC | 0.88 |
| XC_RS11710 | R | CCCCTCTC | -12 | -5 | CCCCCCTC | 0.88 |
| XC_RS11710 | R | CCCCGTCC | -13 | -6 | CCCCCTCC | 0.88 |
| XC_RS11715 | D | CCCCGTCC | -46 | -39 | CTCCGTCC | 0.88 |
| XC_RS11730 | R | CCCCTCTC | -16 | -9 | CTCCTCTC | 0.88 |
| XC_RS11735 | D | CCCGTCCC | -34 | -27 | CCCGTGCC | 0.88 |
| XC_RS11745 | R | CCCCTCTC | -120 | -113 | CCCCTATC | 0.88 |
| XC_RS11745 | R | CCCCTCTC | -76 | -69 | CCCCTATC | 0.88 |
| XC_RS11750 | D | CCCCTCTC | -72 | -65 | CCCCTATC | 0.88 |
| XC_RS11750 | D | CCCCTCTC | -28 | -21 | CCCCTATC | 0.88 |
| XC_RS11760 | D | CCCCGTCC | -10 | -3 | CGCCGTCC | 0.88 |
| XC_RS11760 | D | CCCGTCCC | -9 | -2 | GCCGTCCC | 0.88 |
| XC_RS11760 | D | CTCGCCGTC | -12 | -4 | CTCGCCGTC | 1 |
| XC_RS11795 | D | CCCCGTCC | -77 | -70 | CCGCGTCC | 0.88 |
| XC_RS11795 | D | CCCGTCCC | -90 | -83 | CCGGTCCC | 0.88 |
| XC_RS11795 | R | CTCCCGTC | -45 | -38 | CTCCCGGC | 0.88 |
| XC_RS11850 | D | CCCCGTCC | -66 | -59 | CCGCGTCC | 0.88 |
| XC_RS11850 | D | CCCCGTCC | -47 | -40 | CCCCGGCC | 0.88 |
| XC_RS11850 | R | CCCCGTCC | -96 | -89 | CCCCATCC | 0.88 |
| XC_RS11850 | D | CCCGTCCC | -65 | -58 | CGCGTCCC | 0.88 |
| XC_RS11850 | D | CCCGTCCC | -46 | -39 | CCCGGCCC | 0.88 |
| XC_RS11850 | R | CCCGTCCC | -97 | -90 | CCCATCCC | 0.88 |
| XC_RS11870 | R | CCCCGTCC | -57 | -50 | CCCCGTCC | 1 |
| XC_RS11870 | R | CCCGTCCC | -58 | -51 | CCCGTCCA | 0.88 |
| XC_RS11875 | D | CCCCGTCC | -158 | -151 | CCCCGTCC | 1 |
| XC_RS11875 | D | CCCGTCCC | -157 | -150 | CCCGTCCA | 0.88 |
| XC_RS11880 | R | CCCGTCCC | -99 | -92 | CCCGTCGC | 0.88 |
| XC_RS11880 | R | CTCCCGTC | -97 | -90 | CGCCCGTC | 0.88 |
| XC_RS11885 | R | CCCGTCCC | -107 | -100 | CCTGTCCC | 0.88 |
| XC_RS11890 | D | CCCGTCCC | -65 | -58 | CCTGTCCC | 0.88 |
| XC_RS11900 | D | CCCGTCCC | -189 | -182 | CCCGACCC | 0.88 |
| XC_RS11905 | D | CCCCGTCC | -28 | -21 | CCCCGCCC | 0.88 |
| XC_RS11905 | D | CCCGTCCC | -27 | -20 | CCCGCCCC | 0.88 |
| XC_RS11915 | R | CTCGCCGTC | -105 | -97 | CTCGCCGTG | 0.89 |
| XC_RS11970 | D | CCCCTCTC | -28 | -21 | CCCCACTC | 0.88 |
| XC_RS11970 | R | CCCCGTCC | -92 | -85 | CCGCGTCC | 0.88 |
| XC_RS11970 | R | CCCGTCCC | -93 | -86 | CGCGTCCC | 0.88 |
| XC_RS11975 | R | CCCCTCTC | -88 | -81 | CCCCACTC | 0.88 |
| XC_RS11975 | D | CCCCGTCC | -24 | -17 | CCGCGTCC | 0.88 |
| XC_RS11975 | D | CCCGTCCC | -23 | -16 | CGCGTCCC | 0.88 |
| XC_RS11980 | D | CCCCGTCC | -22 | -15 | CCGCGTCC | 0.88 |
| XC_RS11980 | D | CCCGTCCC | -21 | -14 | CGCGTCCC | 0.88 |
| XC_RS12000 | D | CCCCTCTC | -47 | -40 | TCCCTCTC | 0.88 |
| XC_RS12000 | R | CCCCGTCC | -28 | -21 | CCCCGTCC | 1 |
| XC_RS12000 | R | CCCGTCCC | -29 | -22 | CCCGTCCG | 0.88 |
| XC_RS12000 | R | CCCCCGCCTC | -59 | -50 | CCCCCGCATC | 0.9 |
| XC_RS12030 | D | CTCCCGTC | -58 | -51 | CTGCCGTC | 0.88 |
| XC_RS12065 | D | CTCCCGTC | -162 | -155 | CTCACGTC | 0.88 |
| XC_RS12160 | D | CCCCTCTC | -113 | -106 | TCCCTCTC | 0.88 |
| XC_RS12165 | R | CCCGTCCC | -144 | -137 | CCTGTCCC | 0.88 |
| XC_RS12165 | D | AGGCGGAGG | -139 | -131 | AGGCGAAGG | 0.89 |
| XC_RS12165 | R | AGGCGGAGG | -29 | -21 | AGGCGGTGG | 0.89 |
| XC_RS12170 | R | CCCCTCTC | -102 | -95 | CCACTCTC | 0.88 |
| XC_RS12175 | D | CCCCTCTC | -182 | -175 | CCACTCTC | 0.88 |
| XC_RS12185 | R | CCCCTCTC | -11 | -4 | CGCCTCTC | 0.88 |
| XC_RS12225 | D | CCCGTCCC | -57 | -50 | CCCGACCC | 0.88 |
| XC_RS12250 | R | CCCCTCTC | -184 | -177 | CCCCTCTT | 0.88 |
| XC_RS12250 | D | CCCCGTCC | -197 | -190 | CCCCCTCC | 0.88 |
| XC_RS12255 | D | CCCCTCTC | -185 | -178 | CCCCTCTT | 0.88 |
| XC_RS12255 | R | CCCCGTCC | -172 | -165 | CCCCCTCC | 0.88 |
| XC_RS12255 | D | CCCGTCCC | -26 | -19 | GCCGTCCC | 0.88 |
| XC_RS12260 | D | CCCCGTCC | -135 | -128 | CCCCGACC | 0.88 |
| XC_RS12260 | D | CCCGTCCC | -134 | -127 | CCCGACCC | 0.88 |
| XC_RS12300 | R | CCCCTCTC | -61 | -54 | CCCCTCCC | 0.88 |
| XC_RS12300 | R | CCCCGTCC | -66 | -59 | CCCCGCCC | 0.88 |
| XC_RS12300 | R | CCCCGTCC | -27 | -20 | CGCCGTCC | 0.88 |
| XC_RS12300 | R | CCCGTCCC | -61 | -54 | CCCCTCCC | 0.88 |
| XC_RS12305 | D | CCCCTCTC | -65 | -58 | CCCCTCCC | 0.88 |
| XC_RS12305 | D | CCCCGTCC | -99 | -92 | CGCCGTCC | 0.88 |
| XC_RS12305 | D | CCCCGTCC | -60 | -53 | CCCCGCCC | 0.88 |
| XC_RS12305 | D | CCCGTCCC | -65 | -58 | CCCCTCCC | 0.88 |
| XC_RS12320 | D | CCCCGTCC | -169 | -162 | CCCCGTCT | 0.88 |
| XC_RS12320 | D | CCCGTCCC | -174 | -167 | CCCGACCC | 0.88 |
| XC_RS12325 | R | CTCCCGTC | -14 | -7 | CTCCTGTC | 0.88 |
| XC_RS12330 | D | CCCCGTCC | -137 | -130 | CCCTGTCC | 0.88 |
| XC_RS12335 | D | CTCGCCGTC | -65 | -57 | CCCGCCGTC | 0.89 |
| XC_RS12345 | R | CCCCGTCC | -108 | -101 | CCCCGTCA | 0.88 |
| XC_RS12345 | R | CCCGTCCC | -109 | -102 | CCCGTCAC | 0.88 |
| XC_RS12385 | D | CCCGTCCC | -29 | -22 | GCCGTCCC | 0.88 |
| XC_RS12390 | D | CCCGTCCC | -100 | -93 | CGCGTCCC | 0.88 |
| XC_RS12390 | D | CTCCCGTC | -53 | -46 | CTCCCGTG | 0.88 |
| XC_RS12395 | R | CCCGTCCC | -128 | -121 | CGCGTCCC | 0.88 |
| XC_RS12395 | R | CTCCCGTC | -175 | -168 | CTCCCGTG | 0.88 |
| XC_RS12400 | R | CCCCGTCC | -130 | -123 | CCGCGTCC | 0.88 |
| XC_RS12415 | D | CCTCTCTCCC | -11 | -2 | CCTCTCACCC | 0.9 |
| XC_RS12435 | D | CCCCGTCC | -39 | -32 | CCCCGTTC | 0.88 |
| XC_RS12445 | R | CCCCTCTC | -18 | -11 | CCCCTCGC | 0.88 |
| XC_RS12445 | R | CTCCCGTC | -16 | -9 | CTCCCCTC | 0.88 |
| XC_RS12445 | D | AGGCGGAGG | -10 | -2 | AGCCGGAGG | 0.89 |
| XC_RS12450 | D | CTCCCGTC | -162 | -155 | CTCCCGCC | 0.88 |
| XC_RS12465 | D | CCCCGTCC | -69 | -62 | CCCCTTCC | 0.88 |
| XC_RS12465 | D | CCCGTCCC | -68 | -61 | CCCTTCCC | 0.88 |
| XC_RS12465 | D | CTCGCCGTC | -43 | -35 | CTGGCCGTC | 0.89 |
| XC_RS12465 | D | CCCCTTCTCC | -69 | -60 | CCCCTTCCCC | 0.9 |
| XC_RS12485 | D | AGGCGGAGG | -98 | -90 | AGGCGGCGG | 0.89 |
| XC_RS12485 | R | CCCCCGCCTC | -99 | -90 | CCGCCGCCTC | 0.9 |
| XC_RS12505 | R | CTCCCGTC | -25 | -18 | CTCCTGTC | 0.88 |
| XC_RS12510 | D | CCCCTCTC | -27 | -20 | CGCCTCTC | 0.88 |
| XC_RS12515 | D | CCCCGTCC | -69 | -62 | CCGCGTCC | 0.88 |
| XC_RS12515 | D | CCCCTTCTCC | -27 | -18 | CCCCTGCTCC | 0.9 |
| XC_RS12525 | R | CTCCCGTC | -30 | -23 | CTCCCTTC | 0.88 |
| XC_RS12545 | D | CTCGCCGTC | -24 | -16 | CTCGCCGTC | 1 |
| XC_RS12550 | D | CCCCGTCC | -87 | -80 | GCCCGTCC | 0.88 |
| XC_RS12550 | D | CCCGTCCC | -86 | -79 | CCCGTCCC | 1 |
| XC_RS12550 | D | AGGCGGAGG | -109 | -101 | AGGCGGAAG | 0.89 |
| XC_RS12555 | R | CCCCGTCC | -58 | -51 | GCCCGTCC | 0.88 |
| XC_RS12555 | R | CCCGTCCC | -59 | -52 | CCCGTCCC | 1 |
| XC_RS12555 | R | AGGCGGAGG | -37 | -29 | AGGCGGAAG | 0.89 |
| XC_RS12565 | D | CCCCTCTC | -61 | -54 | CCCCTCTT | 0.88 |
| XC_RS12580 | R | CCCCGTCC | -99 | -92 | CGCCGTCC | 0.88 |
| XC_RS12580 | R | CCCCGTCC | -32 | -25 | CGCCGTCC | 0.88 |
| XC_RS12580 | R | CCCGTCCC | -100 | -93 | GCCGTCCC | 0.88 |
| XC_RS12610 | D | CCCCGTCC | -83 | -76 | ACCCGTCC | 0.88 |
| XC_RS12610 | D | CCCGTCCC | -82 | -75 | CCCGTCCT | 0.88 |
| XC_RS12610 | R | CTCGCCGTC | -193 | -185 | CTGGCCGTC | 0.89 |
| XC_RS12620 | R | CCCGTCCC | -64 | -57 | CCGGTCCC | 0.88 |
| XC_RS12630 | D | CCCGTCCC | -147 | -140 | CCCGCCCC | 0.88 |
| XC_RS12710 | D | CCCCTCTC | -45 | -38 | CCCCTCTC | 1 |
| XC_RS12710 | R | CCCCTCTC | -30 | -23 | CCTCTCTC | 0.88 |
| XC_RS12710 | R | CCCCTTCTCC | -191 | -182 | CACCTTCTCC | 0.9 |
| XC_RS12710 | D | CCTCTCTCCC | -45 | -36 | CCCCTCTCCC | 0.9 |
| XC_RS12710 | R | CCTCTCTCCC | -32 | -23 | CCTCTCTCCC | 1 |
| XC_RS12715 | R | CCCCGTCC | -129 | -122 | CCCCGGCC | 0.88 |
| XC_RS12715 | D | CCCGTCCC | -45 | -38 | CCCGTCAC | 0.88 |
| XC_RS12715 | D | CTCCCGTC | -47 | -40 | CGCCCGTC | 0.88 |
| XC_RS12740 | R | CCCCGTCC | -123 | -116 | CCCCGGCC | 0.88 |
| XC_RS12740 | R | CCCGTCCC | -117 | -110 | CCCATCCC | 0.88 |
| XC_RS12750 | R | CTCCCGTC | -22 | -15 | CTGCCGTC | 0.88 |
| XC_RS12750 | D | CCTCTCTCCC | -34 | -25 | CGTCTCTCCC | 0.9 |
| XC_RS22975 | D | CTCCCGTC | -47 | -40 | CTGCCGTC | 0.88 |
| XC_RS22975 | R | CCTCTCTCCC | -37 | -28 | CGTCTCTCCC | 0.9 |
| XC_RS12760 | R | CTCGCCGTC | -12 | -4 | CTCGCTGTC | 0.89 |
| XC_RS12770 | D | CCCCTCTC | -37 | -30 | CCCATCTC | 0.88 |
| XC_RS12770 | D | CTCCCGTC | -143 | -136 | CTGCCGTC | 0.88 |
| XC_RS12775 | R | AGGCGGAGG | -130 | -122 | TGGCGGAGG | 0.89 |
| XC_RS12805 | D | AGGCGGAGG | -103 | -95 | AGGCGGCGG | 0.89 |
| XC_RS12845 | R | CCCGTCCC | -119 | -112 | CCCGCCCC | 0.88 |
| XC_RS12850 | R | CCCCTCTC | -117 | -110 | CCCATCTC | 0.88 |
| XC_RS12850 | R | CCCGTCCC | -82 | -75 | CCCGTCAC | 0.88 |
| XC_RS12855 | D | CCCCTCTC | -9 | -2 | CCCCGCTC | 0.88 |
| XC_RS12945 | R | CCCCTCTC | -172 | -165 | CCCCTATC | 0.88 |
| XC_RS12945 | D | AGGCGGAGG | -80 | -72 | AGGCGGCGG | 0.89 |
| XC_RS12965 | R | CTCCCGTC | -143 | -136 | CTCCCGCC | 0.88 |
| XC_RS22985 | D | CCCCTCTC | -149 | -142 | CCACTCTC | 0.88 |
| XC_RS22985 | R | CCCCTCTC | -180 | -173 | CCCTTCTC | 0.88 |
| XC_RS22995 | R | CCCGTCCC | -61 | -54 | CCAGTCCC | 0.88 |
| XC_RS13010 | D | CTCGCCGTC | -9 | -1 | CTCGCCGGC | 0.89 |
| XC_RS13075 | R | CTCCCGTC | -127 | -120 | CTCCCGTA | 0.88 |
| XC_RS13095 | R | CTCCCGTC | -64 | -57 | CTACCGTC | 0.88 |
| XC_RS13100 | R | CTCCCGTC | -125 | -118 | CTCGCGTC | 0.88 |
| XC_RS13100 | R | AGGCGGAGG | -140 | -132 | AGGCGTAGG | 0.89 |
| XC_RS13110 | D | CTCCCGTC | -50 | -43 | CTCCCGTG | 0.88 |
| XC_RS13125 | R | AGGCGGAGG | -136 | -128 | AGGAGGAGG | 0.89 |
| XC_RS13130 | D | CTCGCCGTC | -32 | -24 | CTCGCCGTC | 1 |
| XC_RS13150 | R | CTCCCGTC | -38 | -31 | CTCCCGTT | 0.88 |
| XC_RS13165 | R | CCCCGTCC | -109 | -102 | CCGCGTCC | 0.88 |
| XC_RS13170 | D | CCCCGTCC | -10 | -3 | CCGCGTCC | 0.88 |
| XC_RS13190 | D | AGGCGGAGG | -145 | -137 | AGGCGGCGG | 0.89 |
| XC_RS13190 | D | AGGCGGAGG | -115 | -107 | AGCCGGAGG | 0.89 |
| XC_RS23020 | R | CTCCCGTC | -117 | -110 | CTCACGTC | 0.88 |
| XC_RS13240 | D | CCCCTCTC | -25 | -18 | TCCCTCTC | 0.88 |
| XC_RS13260 | R | CCCCTCTC | -16 | -9 | CCCCTCTC | 1 |
| XC_RS13275 | R | CCCCCGCCTC | -86 | -77 | CGCCCGCCTC | 0.9 |
| XC_RS13330 | R | CTCCCGTC | -14 | -7 | CTCCTGTC | 0.88 |
| XC_RS13335 | D | CCCCTCTC | -152 | -145 | CCCCTCCC | 0.88 |
| XC_RS13335 | D | CCCGTCCC | -152 | -145 | CCCCTCCC | 0.88 |
| XC_RS13335 | D | CTCCCGTC | -154 | -147 | CTCCCCTC | 0.88 |
| XC_RS13340 | D | CCCCCGCCTC | -20 | -11 | CCCCCGCCGC | 0.9 |
| XC_RS13380 | R | CTCCCGTC | -87 | -80 | CTCCCGCC | 0.88 |
| XC_RS13385 | D | CCCCGTCC | -44 | -37 | GCCCGTCC | 0.88 |
| XC_RS13385 | D | CCCGTCCC | -43 | -36 | CCCGTCCA | 0.88 |
| XC_RS13420 | D | AGGCGGAGG | -174 | -166 | AGGCGCAGG | 0.89 |
| XC_RS13420 | D | AGGCGGAGG | -59 | -51 | AGGCGCAGG | 0.89 |
| XC_RS13425 | R | CCCCGTCC | -114 | -107 | CCCCGACC | 0.88 |
| XC_RS13440 | D | AGGCGGAGG | -83 | -75 | AGGCGCAGG | 0.89 |
| XC_RS13440 | D | CTCGCCGTC | -153 | -145 | CTCGCCGTA | 0.89 |
| XC_RS13445 | R | AGGCGGAGG | -87 | -79 | AGGCGCAGG | 0.89 |
| XC_RS13445 | R | CTCGCCGTC | -17 | -9 | CTCGCCGTA | 0.89 |
| XC_RS13490 | D | CCCCTCTC | -29 | -22 | CCCCTGTC | 0.88 |
| XC_RS13490 | D | CCCCGTCC | -28 | -21 | CCCTGTCC | 0.88 |
| XC_RS13495 | D | CCCCTCTC | -157 | -150 | CGCCTCTC | 0.88 |
| XC_RS13495 | R | CTCCCGTC | -75 | -68 | CTCCCGCC | 0.88 |
| XC_RS13495 | D | CCTCTCTCCC | -155 | -146 | CCTCTCTGCC | 0.9 |
| XC_RS13515 | R | CCCCGTCC | -170 | -163 | CCCCGCCC | 0.88 |
| XC_RS13515 | R | CCCGTCCC | -171 | -164 | CCCGCCCC | 0.88 |
| XC_RS13525 | D | CCCCGTCC | -107 | -100 | CCCCGCCC | 0.88 |
| XC_RS13535 | D | CTCGCCGTC | -95 | -87 | CTCGCCGGC | 0.89 |
| XC_RS13575 | D | CCCCGTCC | -35 | -28 | ACCCGTCC | 0.88 |
| XC_RS13575 | D | CCCGTCCC | -34 | -27 | CCCGTCCT | 0.88 |
| XC_RS13580 | R | CCCCGTCC | -137 | -130 | ACCCGTCC | 0.88 |
| XC_RS13580 | R | CCCGTCCC | -138 | -131 | CCCGTCCT | 0.88 |
| XC_RS13615 | D | CCCCGTCC | -30 | -23 | CCCCGTCA | 0.88 |
| XC_RS13620 | R | CCCCGTCC | -140 | -133 | CCCGGTCC | 0.88 |
| XC_RS13620 | R | CCCGTCCC | -135 | -128 | CGCGTCCC | 0.88 |
| XC_RS13620 | D | CCTCTCTCCC | -158 | -149 | TCTCTCTCCC | 0.9 |
| XC_RS13625 | D | CTCCCGTC | -105 | -98 | CTCCCGCC | 0.88 |
| XC_RS13640 | R | CCCGTCCC | -38 | -31 | TCCGTCCC | 0.88 |
| XC_RS13650 | R | CCCCGTCC | -49 | -42 | CGCCGTCC | 0.88 |
| XC_RS13650 | D | CTCGCCGTC | -72 | -64 | CTGGCCGTC | 0.89 |
| XC_RS13660 | D | CCCCGTCC | -66 | -59 | CCCCGGCC | 0.88 |
| XC_RS13660 | D | CTCCCGTC | -127 | -120 | CTCCCGGC | 0.88 |
| XC_RS13700 | R | CCCCTCTC | -14 | -7 | CCCCTCCC | 0.88 |
| XC_RS13700 | R | CCCGTCCC | -14 | -7 | CCCCTCCC | 0.88 |
| XC_RS13705 | D | CCCCTCTC | -60 | -53 | CCCCTCCC | 0.88 |
| XC_RS13705 | D | CCCGTCCC | -60 | -53 | CCCCTCCC | 0.88 |
| XC_RS13715 | D | CCCGTCCC | -23 | -16 | CCCGTCAC | 0.88 |
| XC_RS13715 | D | CTCGCCGTC | -70 | -62 | CGCGCCGTC | 0.89 |
| XC_RS23660 | D | CCCGTCCC | -100 | -93 | CCCATCCC | 0.88 |
| XC_RS13735 | D | CCCGTCCC | -187 | -180 | CCCGTTCC | 0.88 |
| XC_RS13740 | D | CCCGTCCC | -185 | -178 | CCCGTCGC | 0.88 |
| XC_RS13800 | R | CCCCGTCC | -18 | -11 | CCGCGTCC | 0.88 |
| XC_RS13820 | R | CCCCGTCC | -79 | -72 | CCCTGTCC | 0.88 |
| XC_RS13820 | D | CTCCCGTC | -113 | -106 | CTCGCGTC | 0.88 |
| XC_RS13825 | D | CCCCTCTC | -99 | -92 | CCCCTCTC | 1 |
| XC_RS13830 | D | CCCGTCCC | -11 | -4 | CCCGTCAC | 0.88 |
| XC_RS13840 | D | CCCCTCTC | -112 | -105 | CCCCTCAC | 0.88 |
| XC_RS13850 | R | CCCGTCCC | -69 | -62 | CGCGTCCC | 0.88 |
| XC_RS13855 | R | CCCCTCTC | -48 | -41 | CCCCACTC | 0.88 |
| XC_RS13875 | D | CTCGCCGTC | -65 | -57 | CTCGCCGGC | 0.89 |
| XC_RS13905 | D | CCCCTCTC | -28 | -21 | CCCTTCTC | 0.88 |
| XC_RS13905 | D | CCCCTTCTCC | -29 | -20 | TCCCTTCTCC | 0.9 |
| XC_RS13930 | D | CCCCTCTC | -31 | -24 | CCCGTCTC | 0.88 |
| XC_RS13930 | D | CCCGTCCC | -31 | -24 | CCCGTCTC | 0.88 |
| XC_RS13930 | D | CTCCCGTC | -50 | -43 | CTCCCGGC | 0.88 |
| XC_RS13935 | R | CCCCTCTC | -65 | -58 | CCCGTCTC | 0.88 |
| XC_RS13935 | R | CCCGTCCC | -65 | -58 | CCCGTCTC | 0.88 |
| XC_RS13935 | R | CTCCCGTC | -46 | -39 | CTCCCGGC | 0.88 |
| XC_RS13945 | D | CTCCCGTC | -24 | -17 | CGCCCGTC | 0.88 |
| XC_RS13950 | R | CTCCCGTC | -44 | -37 | CGCCCGTC | 0.88 |
| XC_RS23075 | R | CCCCGTCC | -48 | -41 | ACCCGTCC | 0.88 |
| XC_RS23075 | R | CCCGTCCC | -49 | -42 | CCCGTCCG | 0.88 |
| XC_RS23075 | R | CTCCCGTC | -47 | -40 | CACCCGTC | 0.88 |
| XC_RS13985 | D | CTCCCGTC | -72 | -65 | CTGCCGTC | 0.88 |
| XC_RS13990 | R | CTCCCGTC | -190 | -183 | CTGCCGTC | 0.88 |
| XC_RS13995 | D | CCCCGTCC | -187 | -180 | CCCCGTGC | 0.88 |
| XC_RS13995 | D | CCCGTCCC | -186 | -179 | CCCGTGCC | 0.88 |
| XC_RS23085 | R | CTCCCGTC | -110 | -103 | CTCCCATC | 0.88 |
| XC_RS14060 | R | CCCCGTCC | -65 | -58 | CCCCGTGC | 0.88 |
| XC_RS14060 | R | CCCGTCCC | -66 | -59 | CCCGTGCC | 0.88 |
| XC_RS14060 | D | AGGCGGAGG | -43 | -35 | AGGCGGCGG | 0.89 |
| XC_RS14060 | R | CCCCCGCCTC | -44 | -35 | CCGCCGCCTC | 0.9 |
| XC_RS14065 | D | CCCCGTCC | -88 | -81 | CCCCGTGC | 0.88 |
| XC_RS14065 | D | CCCGTCCC | -87 | -80 | CCCGTGCC | 0.88 |
| XC_RS14065 | R | AGGCGGAGG | -111 | -103 | AGGCGGCGG | 0.89 |
| XC_RS14065 | D | CCCCCGCCTC | -111 | -102 | CCGCCGCCTC | 0.9 |
| XC_RS14090 | R | CCCCGTCC | -54 | -47 | CCCCGCCC | 0.88 |
| XC_RS23095 | D | CTCGCCGTC | -83 | -75 | CTCGCTGTC | 0.89 |
| XC_RS14120 | D | CCCCTCTC | -30 | -23 | CCCGTCTC | 0.88 |
| XC_RS14120 | D | CCCCGTCC | -31 | -24 | CCCCGTCT | 0.88 |
| XC_RS14120 | R | CCCCGTCC | -156 | -149 | CCCCGTCT | 0.88 |
| XC_RS14120 | D | CCCGTCCC | -30 | -23 | CCCGTCTC | 0.88 |
| XC_RS14120 | R | CCCGTCCC | -182 | -175 | CCCGGCCC | 0.88 |
| XC_RS14120 | D | CTCCCGTC | -32 | -25 | CCCCCGTC | 0.88 |
| XC_RS14120 | D | CTCCCGTC | -21 | -14 | CTCGCGTC | 0.88 |
| XC_RS14120 | R | CTCCCGTC | -155 | -148 | CCCCCGTC | 0.88 |
| XC_RS14120 | D | CTCGCCGTC | -61 | -53 | CTCGCCGCC | 0.89 |
| XC_RS14120 | D | CCCCTTCTCC | -31 | -22 | CCCCGTCTCC | 0.9 |
| XC_RS14120 | D | CCCCCGCCTC | -32 | -23 | CCCCCGTCTC | 0.9 |
| XC_RS14135 | R | CTCCCGTC | -176 | -169 | CTGCCGTC | 0.88 |
| XC_RS14145 | R | CCCCGTCC | -49 | -42 | CGCCGTCC | 0.88 |
| XC_RS14150 | D | CTCGCCGTC | -40 | -32 | CACGCCGTC | 0.89 |
| XC_RS14155 | R | CTCGCCGTC | -113 | -105 | CACGCCGTC | 0.89 |
| XC_RS14165 | R | CCCCGTCC | -24 | -17 | CCCCATCC | 0.88 |
| XC_RS14170 | D | CCCCGTCC | -31 | -24 | CCCCATCC | 0.88 |
| XC_RS14175 | D | CCCCGTCC | -34 | -27 | CCCCGACC | 0.88 |
| XC_RS14190 | R | AGGCGGAGG | -21 | -13 | AGGTGGAGG | 0.89 |
| XC_RS14200 | D | CCCGTCCC | -27 | -20 | CCTGTCCC | 0.88 |
| XC_RS14205 | R | CCCGTCCC | -129 | -122 | CCCGTTCC | 0.88 |
| XC_RS14205 | R | AGGCGGAGG | -76 | -68 | AGGCGGAGC | 0.89 |
| XC_RS14220 | R | CCCCTCTC | -166 | -159 | TCCCTCTC | 0.88 |
| XC_RS14220 | D | CCCGTCCC | -136 | -129 | CCCGGCCC | 0.88 |
| XC_RS14220 | D | CTCCCGTC | -82 | -75 | CTCCCGGC | 0.88 |
| XC_RS14225 | R | CCCGTCCC | -127 | -120 | CCTGTCCC | 0.88 |
| XC_RS14225 | R | CTCCCGTC | -186 | -179 | CACCCGTC | 0.88 |
| XC_RS14225 | R | CTCCCGTC | -90 | -83 | CTCGCGTC | 0.88 |
| XC_RS14230 | D | CCCGTCCC | -102 | -95 | CCTGTCCC | 0.88 |
| XC_RS14230 | D | CTCCCGTC | -139 | -132 | CTCGCGTC | 0.88 |
| XC_RS14230 | D | CTCCCGTC | -43 | -36 | CACCCGTC | 0.88 |
| XC_RS14260 | D | CCCGTCCC | -94 | -87 | CCCGTTCC | 0.88 |
| XC_RS14270 | D | CCCCTCTC | -55 | -48 | GCCCTCTC | 0.88 |
| XC_RS14285 | D | CCCCGTCC | -35 | -28 | CCCCGTCG | 0.88 |
| XC_RS14320 | R | CCCCTCTC | -144 | -137 | ACCCTCTC | 0.88 |
| XC_RS14320 | R | CTCCCGTC | -169 | -162 | CTACCGTC | 0.88 |
| XC_RS14335 | R | CCCCTCTC | -148 | -141 | CCCTTCTC | 0.88 |
| XC_RS14335 | D | CCCGTCCC | -189 | -182 | CCCGTACC | 0.88 |
| XC_RS14335 | D | CCCCTTCTCC | -164 | -155 | CACCTTCTCC | 0.9 |
| XC_RS14335 | R | CCCCTTCTCC | -149 | -140 | TCCCTTCTCC | 0.9 |
| XC_RS14365 | D | CCCGTCCC | -34 | -27 | CCCGGCCC | 0.88 |
| XC_RS14370 | D | CTCCCGTC | -170 | -163 | CTGCCGTC | 0.88 |
| XC_RS14380 | D | CCCGTCCC | -98 | -91 | CCGGTCCC | 0.88 |
| XC_RS14380 | R | CTCGCCGTC | -74 | -66 | CTCGCCGGC | 0.89 |
| XC_RS14385 | R | CCCGTCCC | -141 | -134 | CCGGTCCC | 0.88 |
| XC_RS14385 | D | CTCGCCGTC | -166 | -158 | CTCGCCGGC | 0.89 |
| XC_RS14420 | D | CTCCCGTC | -117 | -110 | CTCGCGTC | 0.88 |
| XC_RS14420 | R | CTCCCGTC | -126 | -119 | GTCCCGTC | 0.88 |
| XC_RS14485 | D | CCCCTCTC | -26 | -19 | CCCCACTC | 0.88 |
| XC_RS14485 | D | CCCCGTCC | -58 | -51 | CTCCGTCC | 0.88 |
| XC_RS14485 | D | CCCGTCCC | -57 | -50 | TCCGTCCC | 0.88 |
| XC_RS14495 | D | CTCCCGTC | -40 | -33 | CTGCCGTC | 0.88 |
| XC_RS14495 | R | AGGCGGAGG | -127 | -119 | AGGCGGCGG | 0.89 |
| XC_RS14500 | D | CCCCGTCC | -64 | -57 | CCCCGTAC | 0.88 |
| XC_RS14520 | D | CTCCCGTC | -74 | -67 | CTACCGTC | 0.88 |
| XC_RS23110 | R | CTCCCGTC | -191 | -184 | CTTCCGTC | 0.88 |
| XC_RS14530 | D | CCCCTCTC | -87 | -80 | CCCCACTC | 0.88 |
| XC_RS14535 | D | CCCGTCCC | -186 | -179 | CCCATCCC | 0.88 |
| XC_RS14565 | D | CCCCTCTC | -28 | -21 | CCCCGCTC | 0.88 |
| XC_RS14565 | D | CCCGTCCC | -103 | -96 | CCCGTGCC | 0.88 |
| XC_RS14575 | R | CCCCGTCC | -29 | -22 | CCCCATCC | 0.88 |
| XC_RS14580 | D | CCCCGTCC | -118 | -111 | CCCCATCC | 0.88 |
| XC_RS14590 | R | CCCCTCTC | -12 | -5 | CCCCTCCC | 0.88 |
| XC_RS14590 | R | CCCGTCCC | -12 | -5 | CCCCTCCC | 0.88 |
| XC_RS14600 | R | CCCCGTCC | -91 | -84 | CCCCGACC | 0.88 |
| XC_RS14600 | D | CTCCCGTC | -77 | -70 | CTCCCGGC | 0.88 |
| XC_RS14605 | R | CCCCTCTC | -145 | -138 | CCCCACTC | 0.88 |
| XC_RS14605 | D | CCCCGTCC | -113 | -106 | CCCTGTCC | 0.88 |
| XC_RS14605 | D | CCCCGTCC | -10 | -3 | CCCCGCCC | 0.88 |
| XC_RS14605 | D | CCCGTCCC | -9 | -2 | CCCGCCCC | 0.88 |
| XC_RS14615 | D | CCCCGTCC | -161 | -154 | CCCCGACC | 0.88 |
| XC_RS14615 | D | CTCCCGTC | -66 | -59 | CTCCCGCC | 0.88 |
| XC_RS14615 | D | CTCGCCGTC | -109 | -101 | CTCGCCGCC | 0.89 |
| XC_RS14625 | R | AGGCGGAGG | -21 | -13 | TGGCGGAGG | 0.89 |
| XC_RS14675 | R | CTCCCGTC | -183 | -176 | CTCCCCTC | 0.88 |
| XC_RS14680 | D | CTCCCGTC | -32 | -25 | CTCCCCTC | 0.88 |
| XC_RS14710 | D | CCCCTCTC | -76 | -69 | CCCCTTTC | 0.88 |
| XC_RS14760 | D | CTCCCGTC | -98 | -91 | CTCCCGCC | 0.88 |
| XC_RS14765 | D | CTCGCCGTC | -141 | -133 | CCCGCCGTC | 0.89 |
| XC_RS23130 | R | CCCCGTCC | -154 | -147 | CCCCTTCC | 0.88 |
| XC_RS23130 | R | CCCGTCCC | -155 | -148 | CCCTTCCC | 0.88 |
| XC_RS14785 | D | CCCCGTCC | -12 | -5 | CCCCTTCC | 0.88 |
| XC_RS14785 | D | CCCGTCCC | -18 | -11 | CCCGACCC | 0.88 |
| XC_RS14810 | D | CTCCCGTC | -102 | -95 | CTCCCGCC | 0.88 |
| XC_RS14815 | R | CCCCGTCC | -98 | -91 | CCCTGTCC | 0.88 |
| XC_RS14835 | D | CTCCCGTC | -167 | -160 | CTCACGTC | 0.88 |
| XC_RS23135 | D | CCCCGTCC | -144 | -137 | CCCCGCCC | 0.88 |
| XC_RS14965 | R | CCCCTCTC | -143 | -136 | CCCCACTC | 0.88 |
| XC_RS14970 | D | CTCCCGTC | -74 | -67 | CTCCCGTT | 0.88 |
| XC_RS14970 | D | CTCGCCGTC | -105 | -97 | CTCGCCGTT | 0.89 |
| XC_RS14980 | R | CTCCCGTC | -19 | -12 | CTCCTGTC | 0.88 |
| XC_RS15065 | R | AGGCGGAGG | -66 | -58 | AGGCGAAGG | 0.89 |
| XC_RS15100 | D | CCCCGTCC | -110 | -103 | TCCCGTCC | 0.88 |
| XC_RS15100 | D | CCCGTCCC | -109 | -102 | CCCGTCCT | 0.88 |
| XC_RS15100 | D | CTCCCGTC | -111 | -104 | TTCCCGTC | 0.88 |
| XC_RS15110 | D | CCCCTCTC | -17 | -10 | CCCCTCTG | 0.88 |
| XC_RS15110 | R | CCCGTCCC | -114 | -107 | CCCGTGCC | 0.88 |
| XC_RS15120 | D | CCCCGTCC | -99 | -92 | CCCAGTCC | 0.88 |
| XC_RS15220 | D | CCCCTCTC | -82 | -75 | CCCCTCAC | 0.88 |
| XC_RS15220 | D | CCCCGTCC | -56 | -49 | CGCCGTCC | 0.88 |
| XC_RS15230 | D | CCCGTCCC | -116 | -109 | CCCGTACC | 0.88 |
| XC_RS15295 | D | CCCCGTCC | -126 | -119 | CCCCGCCC | 0.88 |
| XC_RS15295 | D | CTCCCGTC | -111 | -104 | CTCGCGTC | 0.88 |
| XC_RS15295 | D | CTCGCCGTC | -157 | -149 | CCCGCCGTC | 0.89 |
| XC_RS15300 | D | CCCCGTCC | -157 | -150 | CCCCGTGC | 0.88 |
| XC_RS15300 | R | CCCCGTCC | -49 | -42 | CTCCGTCC | 0.88 |
| XC_RS15300 | D | CCCGTCCC | -108 | -101 | CCCGGCCC | 0.88 |
| XC_RS15300 | D | CCCGTCCC | -72 | -65 | CCTGTCCC | 0.88 |
| XC_RS15310 | R | CCCCTCTC | -117 | -110 | CCCCTATC | 0.88 |
| XC_RS15315 | D | CCCCGTCC | -25 | -18 | CCCAGTCC | 0.88 |
| XC_RS15315 | D | CCCGTCCC | -24 | -17 | CCAGTCCC | 0.88 |
| XC_RS15320 | R | CCCGTCCC | -37 | -30 | CCCGGCCC | 0.88 |
| XC_RS15350 | R | CCCCTCTC | -110 | -103 | CCCCTGTC | 0.88 |
| XC_RS15350 | R | CCCCGTCC | -111 | -104 | CCCTGTCC | 0.88 |
| XC_RS15350 | R | CCCCGTCC | -79 | -72 | CCCCGGCC | 0.88 |
| XC_RS15350 | R | CCCCGTCC | -69 | -62 | CCCCGGCC | 0.88 |
| XC_RS15350 | R | CCCGTCCC | -186 | -179 | CCCGGCCC | 0.88 |
| XC_RS15355 | D | CCCCTCTC | -188 | -181 | CCCCTGTC | 0.88 |
| XC_RS15355 | D | CCCCGTCC | -187 | -180 | CCCTGTCC | 0.88 |
| XC_RS15355 | D | CCCGTCCC | -112 | -105 | CCCGGCCC | 0.88 |
| XC_RS15355 | D | CCCGTCCC | -31 | -24 | CCCGGCCC | 0.88 |
| XC_RS15360 | R | CTCGCCGTC | -179 | -171 | CTCGCCGAC | 0.89 |
| XC_RS15365 | R | CTCCCGTC | -72 | -65 | CTCCTGTC | 0.88 |
| XC_RS15365 | D | CTCGCCGTC | -115 | -107 | CTCGCCGAC | 0.89 |
| XC_RS15380 | R | CCCCTCTC | -10 | -3 | CCTCTCTC | 0.88 |
| XC_RS15380 | R | CCTCTCTCCC | -12 | -3 | CCTCTCTCTC | 0.9 |
| XC_RS15470 | R | CTCCCGTC | -13 | -6 | CACCCGTC | 0.88 |
| XC_RS15475 | D | CCTCTCTCCC | -12 | -3 | GCTCTCTCCC | 0.9 |
| XC_RS15500 | D | CCCGTCCC | -26 | -19 | CCCGTACC | 0.88 |
| XC_RS15550 | D | CCCCTTCTCC | -46 | -37 | CCTCTTCTCC | 0.9 |
| XC_RS15560 | D | CCCCTCTC | -106 | -99 | CCCATCTC | 0.88 |
| XC_RS15560 | R | CCCCTCTC | -198 | -191 | CCCATCTC | 0.88 |
| XC_RS15560 | R | CCCCGTCC | -175 | -168 | CCCCGGCC | 0.88 |
| XC_RS15575 | R | CTCGCCGTC | -199 | -191 | CTCTCCGTC | 0.89 |
| XC_RS15585 | D | CTCCCGTC | -60 | -53 | CTCCTGTC | 0.88 |
| XC_RS15655 | D | CCCCTCTC | -24 | -17 | CCCCTCCC | 0.88 |
| XC_RS15655 | D | CCCGTCCC | -24 | -17 | CCCCTCCC | 0.88 |
| XC_RS15660 | R | CCCCTCTC | -112 | -105 | CCCCTCCC | 0.88 |
| XC_RS15660 | R | CCCGTCCC | -112 | -105 | CCCCTCCC | 0.88 |
| XC_RS15695 | D | CCCGTCCC | -96 | -89 | CCCGTCGC | 0.88 |
| XC_RS15775 | R | CCCGTCCC | -141 | -134 | CCCGGCCC | 0.88 |
| XC_RS15785 | D | CCCGTCCC | -66 | -59 | CCAGTCCC | 0.88 |
| XC_RS15790 | R | CCCCTCTC | -112 | -105 | CCCCTCTG | 0.88 |
| XC_RS15805 | D | CCCCGTCC | -94 | -87 | CCCTGTCC | 0.88 |
| XC_RS15805 | D | CCCCTTCTCC | -106 | -97 | CCCCTGCTCC | 0.9 |
| XC_RS23170 | R | CCCCGTCC | -56 | -49 | CCCTGTCC | 0.88 |
| XC_RS23170 | R | CCCCTTCTCC | -46 | -37 | CCCCTGCTCC | 0.9 |
| XC_RS15810 | D | CCCCTCTC | -29 | -22 | ACCCTCTC | 0.88 |
| XC_RS23175 | D | AGGCGGAGG | -30 | -22 | AGGCGGGGG | 0.89 |
| XC_RS23175 | R | CCCCCGCCTC | -31 | -22 | CCCCCGCCTG | 0.9 |
| XC_RS15850 | D | CCCCGTCC | -197 | -190 | CACCGTCC | 0.88 |
| XC_RS15855 | R | CCCCGTCC | -183 | -176 | CACCGTCC | 0.88 |
| XC_RS15870 | D | CCCGTCCC | -39 | -32 | CCTGTCCC | 0.88 |
| XC_RS15885 | R | AGGCGGAGG | -57 | -49 | AGGCGGTGG | 0.89 |
| XC_RS15895 | D | CTCGCCGTC | -129 | -121 | CTCGCCGTT | 0.89 |
| XC_RS15900 | D | CCCCTCTC | -29 | -22 | ACCCTCTC | 0.88 |
| XC_RS15900 | R | CTCGCCGTC | -158 | -150 | CTCGCCGTT | 0.89 |
| XC_RS15930 | D | CTCCCGTC | -50 | -43 | CTGCCGTC | 0.88 |
| XC_RS15935 | R | CTCCCGTC | -99 | -92 | CTGCCGTC | 0.88 |
| XC_RS15965 | R | CCCCTCTC | -155 | -148 | CCCCACTC | 0.88 |
| XC_RS15965 | D | CTCCCGTC | -169 | -162 | CTCCTGTC | 0.88 |
| XC_RS15970 | D | CCCCTCTC | -30 | -23 | CCCCACTC | 0.88 |
| XC_RS15970 | R | CTCCCGTC | -16 | -9 | CTCCTGTC | 0.88 |
| XC_RS16000 | D | CCCCGTCC | -20 | -13 | CCCCGTCC | 1 |
| XC_RS16000 | D | CCCGTCCC | -19 | -12 | CCCGTCCA | 0.88 |
| XC_RS16010 | R | CTCCCGTC | -16 | -9 | CTCGCGTC | 0.88 |
| XC_RS23190 | D | CCCCCGCCTC | -15 | -6 | CCCGCGCCTC | 0.9 |
| XC_RS23195 | R | CCCCCGCCTC | -97 | -88 | CCCGCGCCTC | 0.9 |
| XC_RS16090 | R | AGGCGGAGG | -21 | -13 | AGGCGGAGG | 1 |
| XC_RS16095 | D | CCCCGTCC | -141 | -134 | CCCCGTTC | 0.88 |
| XC_RS16095 | D | CCCGTCCC | -55 | -48 | CCGGTCCC | 0.88 |
| XC_RS16095 | D | CTCCCGTC | -8 | -1 | CACCCGTC | 0.88 |
| XC_RS16110 | D | CCCGTCCC | -167 | -160 | CCCGGCCC | 0.88 |
| XC_RS16110 | R | CCCGTCCC | -38 | -31 | CCAGTCCC | 0.88 |
| XC_RS16110 | R | AGGCGGAGG | -173 | -165 | GGGCGGAGG | 0.89 |
| XC_RS16125 | D | CCCGTCCC | -27 | -20 | CCCGTCAC | 0.88 |
| XC_RS16125 | D | CTCCCGTC | -29 | -22 | CGCCCGTC | 0.88 |
| XC_RS16125 | D | AGGCGGAGG | -126 | -118 | AGGCGAAGG | 0.89 |
| XC_RS16125 | R | CCCCTTCTCC | -125 | -116 | CCCCTTCGCC | 0.9 |
| XC_RS16130 | R | CCCGTCCC | -141 | -134 | CCCGTCAC | 0.88 |
| XC_RS16130 | R | CTCCCGTC | -139 | -132 | CGCCCGTC | 0.88 |
| XC_RS16130 | R | AGGCGGAGG | -43 | -35 | AGGCGAAGG | 0.89 |
| XC_RS16130 | D | CCCCTTCTCC | -45 | -36 | CCCCTTCGCC | 0.9 |
| XC_RS16135 | D | CCCCGTCC | -31 | -24 | CCCCGTCC | 1 |
| XC_RS16135 | D | CCCGTCCC | -30 | -23 | CCCGTCCA | 0.88 |
| XC_RS16135 | D | CTCCCGTC | -32 | -25 | CCCCCGTC | 0.88 |
| XC_RS16160 | R | CCCCTCTC | -179 | -172 | CACCTCTC | 0.88 |
| XC_RS16160 | D | CTCCCGTC | -110 | -103 | CTCCCGAC | 0.88 |
| XC_RS23215 | D | CCCCCGCCTC | -191 | -182 | CCCCCGCCGC | 0.9 |
| XC_RS16175 | D | CCCCGTCC | -28 | -21 | CCCCGTCA | 0.88 |
| XC_RS16175 | D | CCCGTCCC | -27 | -20 | CCCGTCAC | 0.88 |
| XC_RS16195 | D | CCCCGTCC | -28 | -21 | CCCCGCCC | 0.88 |
| XC_RS16220 | R | CCCCGTCC | -115 | -108 | CCCCATCC | 0.88 |
| XC_RS16230 | R | CCCCTCTC | -27 | -20 | CCCCTGTC | 0.88 |
| XC_RS16230 | R | CCCCGTCC | -28 | -21 | CCCTGTCC | 0.88 |
| XC_RS16235 | D | CTCCCGTC | -11 | -4 | CTCGCGTC | 0.88 |
| XC_RS16255 | D | CCCGTCCC | -27 | -20 | TCCGTCCC | 0.88 |
| XC_RS16260 | D | CCCCGTCC | -31 | -24 | CACCGTCC | 0.88 |
| XC_RS16265 | R | CTCCCGTC | -146 | -139 | CTCCCGGC | 0.88 |
| XC_RS16270 | D | CCCCTCTC | -21 | -14 | CCCCTCTT | 0.88 |
| XC_RS16360 | D | CCCCTCTC | -172 | -165 | CCCCACTC | 0.88 |
| XC_RS16365 | R | CTCGCCGTC | -25 | -17 | CTGGCCGTC | 0.89 |
| XC_RS16410 | D | CCCCTCTC | -165 | -158 | CCCCCCTC | 0.88 |
| XC_RS16410 | D | CCCCTTCTCC | -164 | -155 | CCCCCTCTCC | 0.9 |
| XC_RS16415 | D | AGGCGGAGG | -147 | -139 | AGGCGGCGG | 0.89 |
| XC_RS16420 | R | AGGCGGAGG | -59 | -51 | AGGCGGCGG | 0.89 |
| XC_RS16485 | D | CCCCGTCC | -37 | -30 | CCCCGTGC | 0.88 |
| XC_RS16485 | D | CCCGTCCC | -67 | -60 | CCCGACCC | 0.88 |
| XC_RS16485 | R | CCCGTCCC | -89 | -82 | CCCGACCC | 0.88 |
| XC_RS16485 | D | CTCCCGTC | -69 | -62 | CTCCCGAC | 0.88 |
| XC_RS16495 | D | CCCCTCTC | -54 | -47 | CCCGTCTC | 0.88 |
| XC_RS16495 | D | CCCGTCCC | -54 | -47 | CCCGTCTC | 0.88 |
| XC_RS16495 | D | CTCGCCGTC | -69 | -61 | CTCGCCATC | 0.89 |
| XC_RS16530 | D | CCCGTCCC | -132 | -125 | CCCGACCC | 0.88 |
| XC_RS16560 | R | AGGCGGAGG | -44 | -36 | AGGCGGCGG | 0.89 |
| XC_RS16565 | D | CCCCTCTC | -42 | -35 | CGCCTCTC | 0.88 |
| XC_RS16565 | R | AGGCGGAGG | -46 | -38 | AGGCGGCGG | 0.89 |
| XC_RS16565 | D | CCCCCGCCTC | -46 | -37 | CCGCCGCCTC | 0.9 |
| XC_RS16570 | D | CCCCTCTC | -100 | -93 | CCCCACTC | 0.88 |
| XC_RS16570 | D | CCCCTCTC | -82 | -75 | CCCCTCTG | 0.88 |
| XC_RS16570 | R | CTCCCGTC | -15 | -8 | CTCCCGTT | 0.88 |
| XC_RS16580 | D | CTCCCGTC | -18 | -11 | CTCCCGTA | 0.88 |
| XC_RS16590 | R | CCCCTCTC | -55 | -48 | CCCCTGTC | 0.88 |
| XC_RS16590 | D | CCCCGTCC | -199 | -192 | CGCCGTCC | 0.88 |
| XC_RS16590 | R | CCCCGTCC | -56 | -49 | CCCTGTCC | 0.88 |
| XC_RS16590 | R | CCCGTCCC | -57 | -50 | CCTGTCCC | 0.88 |
| XC_RS16605 | R | CTCCCGTC | -68 | -61 | CTCCCCTC | 0.88 |
| XC_RS16630 | D | CCCCTCTC | -25 | -18 | CCCCTCCC | 0.88 |
| XC_RS16630 | R | CCCCGTCC | -125 | -118 | CACCGTCC | 0.88 |
| XC_RS16630 | D | CCCGTCCC | -25 | -18 | CCCCTCCC | 0.88 |
| XC_RS16630 | D | CTCCCGTC | -52 | -45 | CTCCCGTC | 1 |
| XC_RS16630 | R | CTCGCCGTC | -76 | -68 | CTCGCCGCC | 0.89 |
| XC_RS16635 | D | CTCGCCGTC | -40 | -32 | CACGCCGTC | 0.89 |
| XC_RS16640 | R | CTCGCCGTC | -83 | -75 | CACGCCGTC | 0.89 |
| XC_RS16645 | D | CCCCGTCC | -120 | -113 | CCCCGTCC | 1 |
| XC_RS16645 | D | CCCGTCCC | -119 | -112 | CCCGTCCT | 0.88 |
| XC_RS16645 | R | CCCGTCCC | -199 | -192 | CCCGTGCC | 0.88 |
| XC_RS16650 | D | CTCCCGTC | -132 | -125 | CTGCCGTC | 0.88 |
| XC_RS16705 | D | CCCCTCTC | -103 | -96 | CGCCTCTC | 0.88 |
| XC_RS16710 | D | CCCCGTCC | -162 | -155 | CACCGTCC | 0.88 |
| XC_RS16710 | D | CCCGTCCC | -161 | -154 | ACCGTCCC | 0.88 |
| XC_RS16735 | D | CCCCGTCC | -57 | -50 | CCCCGTGC | 0.88 |
| XC_RS16745 | R | CCCCTCTC | -18 | -11 | CCCCTGTC | 0.88 |
| XC_RS16760 | D | CCTCTCTCCC | -27 | -18 | CCTCTTTCCC | 0.9 |
| XC_RS16770 | R | CCCCGTCC | -50 | -43 | CCCCGGCC | 0.88 |
| XC_RS16770 | R | CCCGTCCC | -51 | -44 | CCCGGCCC | 0.88 |
| XC_RS16795 | D | CCCCGTCC | -175 | -168 | CACCGTCC | 0.88 |
| XC_RS16815 | D | CCCCGTCC | -127 | -120 | CCACGTCC | 0.88 |
| XC_RS16815 | D | CCCGTCCC | -98 | -91 | GCCGTCCC | 0.88 |
| XC_RS16815 | D | CTCCCGTC | -139 | -132 | CTCGCGTC | 0.88 |
| XC_RS16830 | D | CCCGTCCC | -64 | -57 | CCCATCCC | 0.88 |
| XC_RS16905 | D | CCCGTCCC | -33 | -26 | CCCGTCGC | 0.88 |
| XC_RS16905 | D | AGGCGGAGG | -117 | -109 | GGGCGGAGG | 0.89 |
| XC_RS16935 | R | CCCCTCTC | -174 | -167 | CACCTCTC | 0.88 |
| XC_RS16935 | D | CCCCGTCC | -195 | -188 | CCCCGGCC | 0.88 |
| XC_RS16935 | D | CTCGCCGTC | -142 | -134 | CTCGCAGTC | 0.89 |
| XC_RS16935 | R | CCCCTTCTCC | -106 | -97 | CGCCTTCTCC | 0.9 |
| XC_RS16945 | D | CCCGTCCC | -28 | -21 | CCAGTCCC | 0.88 |
| XC_RS16955 | D | CCCCGTCC | -54 | -47 | CCCGGTCC | 0.88 |
| XC_RS16955 | R | CCCGTCCC | -69 | -62 | CGCGTCCC | 0.88 |
| XC_RS16990 | D | CCCCGTCC | -21 | -14 | CCCGGTCC | 0.88 |
| XC_RS17010 | R | CCTCTCTCCC | -106 | -97 | CCTCACTCCC | 0.9 |
| XC_RS23240 | D | CCCCTCTC | -39 | -32 | CCGCTCTC | 0.88 |
| XC_RS23240 | D | CCTCTCTCCC | -39 | -30 | CCGCTCTCCC | 0.9 |
| XC_RS17050 | R | CCCCTCTC | -136 | -129 | CCGCTCTC | 0.88 |
| XC_RS17050 | R | CCTCTCTCCC | -138 | -129 | CCGCTCTCCC | 0.9 |
| XC_RS17105 | R | CCCGTCCC | -193 | -186 | CCCGGCCC | 0.88 |
| XC_RS17130 | D | CCCGTCCC | -25 | -18 | CCCGTCGC | 0.88 |
| XC_RS17130 | D | CTCCCGTC | -27 | -20 | CTCCCGTC | 1 |
| XC_RS17135 | R | CCCGTCCC | -156 | -149 | CCCGTCGC | 0.88 |
| XC_RS17135 | R | CTCCCGTC | -154 | -147 | CTCCCGTC | 1 |
| XC_RS23245 | R | CTCCCGTC | -29 | -22 | CGCCCGTC | 0.88 |
| XC_RS17155 | R | CTCCCGTC | -128 | -121 | CTCCCTTC | 0.88 |
| XC_RS17160 | D | CTCCCGTC | -20 | -13 | CTCCCTTC | 0.88 |
| XC_RS23250 | D | CCCCTCTC | -58 | -51 | CCGCTCTC | 0.88 |
| XC_RS17170 | D | CCCGTCCC | -91 | -84 | CCCTTCCC | 0.88 |
| XC_RS17175 | R | CCCGTCCC | -47 | -40 | CCCTTCCC | 0.88 |
| XC_RS17200 | R | CCCGTCCC | -169 | -162 | TCCGTCCC | 0.88 |
| XC_RS17200 | R | CCCCTTCTCC | -184 | -175 | CCCCTTCACC | 0.9 |
| XC_RS17205 | D | CCCGTCCC | -38 | -31 | TCCGTCCC | 0.88 |
| XC_RS17205 | D | CCCCTTCTCC | -25 | -16 | CCCCTTCACC | 0.9 |
| XC_RS17210 | D | CCCCGTCC | -75 | -68 | CCCCTTCC | 0.88 |
| XC_RS17210 | R | CCCCGTCC | -160 | -153 | CCCCGGCC | 0.88 |
| XC_RS17210 | R | CCCGTCCC | -161 | -154 | CCCGGCCC | 0.88 |
| XC_RS17215 | D | CCCGTCCC | -72 | -65 | CCCGGCCC | 0.88 |
| XC_RS17225 | D | CCCCGTCC | -33 | -26 | CCCCGACC | 0.88 |
| XC_RS17225 | D | CCCGTCCC | -32 | -25 | CCCGACCC | 0.88 |
| XC_RS17235 | R | CTCCCGTC | -74 | -67 | CTCCCGGC | 0.88 |
| XC_RS17265 | R | CCCCGTCC | -104 | -97 | CCCCGGCC | 0.88 |
| XC_RS17265 | D | AGGCGGAGG | -196 | -188 | AGGCGGTGG | 0.89 |
| XC_RS17270 | D | CCCCGTCC | -133 | -126 | CCCCGGCC | 0.88 |
| XC_RS17270 | R | AGGCGGAGG | -42 | -34 | AGGCGGTGG | 0.89 |
| XC_RS17290 | R | AGGCGGAGG | -25 | -17 | GGGCGGAGG | 0.89 |
| XC_RS17320 | D | CCCCGTCC | -50 | -43 | CCGCGTCC | 0.88 |
| XC_RS17340 | D | CCCGTCCC | -36 | -29 | CCCGTCAC | 0.88 |
| XC_RS17340 | D | CTCCCGTC | -38 | -31 | CGCCCGTC | 0.88 |
| XC_RS17355 | D | CCCGTCCC | -110 | -103 | GCCGTCCC | 0.88 |
| XC_RS17360 | R | CCCGTCCC | -134 | -127 | GCCGTCCC | 0.88 |
| XC_RS17370 | D | CCCCGTCC | -105 | -98 | CCCCGTTC | 0.88 |
| XC_RS17370 | R | CTCCCGTC | -97 | -90 | CTGCCGTC | 0.88 |
| XC_RS17405 | D | CCCCGTCC | -81 | -74 | CCCCTTCC | 0.88 |
| XC_RS17455 | D | CTCGCCGTC | -123 | -115 | CTCGCCGTT | 0.89 |
| XC_RS17460 | D | CTCGCCGTC | -30 | -22 | CTCGCGGTC | 0.89 |
| XC_RS17485 | R | CCCCTCTC | -66 | -59 | CCCCACTC | 0.88 |
| XC_RS17485 | D | CTCCCGTC | -124 | -117 | CTCCGGTC | 0.88 |
| XC_RS17490 | D | CCCCTCTC | -96 | -89 | CCCCACTC | 0.88 |
| XC_RS17490 | R | CTCCCGTC | -38 | -31 | CTCCGGTC | 0.88 |
| XC_RS17505 | D | CCCCTCTC | -28 | -21 | TCCCTCTC | 0.88 |
| XC_RS17520 | D | CCCCGTCC | -25 | -18 | CCCCGTCC | 1 |
| XC_RS17520 | D | CCCGTCCC | -24 | -17 | CCCGTCCT | 0.88 |
| XC_RS17520 | D | CTCCCGTC | -8 | -1 | CTGCCGTC | 0.88 |
| XC_RS17520 | R | AGGCGGAGG | -15 | -7 | AGGCGGCGG | 0.89 |
| XC_RS17535 | D | CCCCGTCC | -87 | -80 | CGCCGTCC | 0.88 |
| XC_RS17535 | D | CTCGCCGTC | -89 | -81 | ATCGCCGTC | 0.89 |
| XC_RS23270 | D | CCCGTCCC | -79 | -72 | CGCGTCCC | 0.88 |
| XC_RS17545 | D | CCCCCGCCTC | -185 | -176 | GCCCCGCCTC | 0.9 |
| XC_RS17550 | D | CCCCTCTC | -71 | -64 | CCCCTCGC | 0.88 |
| XC_RS23275 | R | CCCGTCCC | -144 | -137 | CCCTTCCC | 0.88 |
| XC_RS23275 | R | CTCCCGTC | -142 | -135 | CTCCCTTC | 0.88 |
| XC_RS23275 | R | CTCCCGTC | -14 | -7 | CTCCCGCC | 0.88 |
| XC_RS17560 | D | CCCGTCCC | -29 | -22 | CCCTTCCC | 0.88 |
| XC_RS17560 | D | CTCCCGTC | -159 | -152 | CTCCCGCC | 0.88 |
| XC_RS17560 | D | CTCCCGTC | -31 | -24 | CTCCCTTC | 0.88 |
| XC_RS17575 | D | CCCCGTCC | -147 | -140 | CCCCGTCT | 0.88 |
| XC_RS23280 | D | CTCCCGTC | -15 | -8 | CTCCCGTG | 0.88 |
| XC_RS17585 | D | CCCCGTCC | -126 | -119 | CCCCGGCC | 0.88 |
| XC_RS17610 | D | CTCCCGTC | -110 | -103 | CGCCCGTC | 0.88 |
| XC_RS17615 | R | CCCCTCTC | -63 | -56 | CCCCTGTC | 0.88 |
| XC_RS17615 | D | CCCCGTCC | -100 | -93 | CCCCGTCC | 1 |
| XC_RS17615 | D | CCCGTCCC | -99 | -92 | CCCGTCCG | 0.88 |
| XC_RS17615 | R | CCCGTCCC | -9 | -2 | CCCTTCCC | 0.88 |
| XC_RS17615 | D | CTCCCGTC | -101 | -94 | CCCCCGTC | 0.88 |
| XC_RS17620 | R | CCCCGTCC | -175 | -168 | CCCCGTCC | 1 |
| XC_RS17620 | R | CCCGTCCC | -176 | -169 | CCCGTCCG | 0.88 |
| XC_RS17620 | R | CTCCCGTC | -174 | -167 | CCCCCGTC | 0.88 |
| XC_RS17665 | D | CCCCTCTC | -90 | -83 | CACCTCTC | 0.88 |
| XC_RS17670 | R | CCCCTCTC | -153 | -146 | CACCTCTC | 0.88 |
| XC_RS17705 | R | CTCCCGTC | -180 | -173 | CTCCCGCC | 0.88 |
| XC_RS17710 | D | CCCCGTCC | -58 | -51 | CCCCGCCC | 0.88 |
| XC_RS17710 | D | CCCGTCCC | -53 | -46 | CCCGTGCC | 0.88 |
| XC_RS17715 | D | CCCCGTCC | -125 | -118 | CCCCGCCC | 0.88 |
| XC_RS17715 | D | CCCGTCCC | -124 | -117 | CCCGCCCC | 0.88 |
| XC_RS17715 | R | CCCGTCCC | -45 | -38 | CCGGTCCC | 0.88 |
| XC_RS17820 | R | CCCCTCTC | -69 | -62 | CCCCTGTC | 0.88 |
| XC_RS17820 | R | CCCCGTCC | -70 | -63 | CCCTGTCC | 0.88 |
| XC_RS17825 | D | CCCCTCTC | -21 | -14 | CCCCTGTC | 0.88 |
| XC_RS17825 | D | CCCCGTCC | -20 | -13 | CCCTGTCC | 0.88 |
| XC_RS17885 | D | CCCCTCTC | -23 | -16 | CCCCTCTC | 1 |
| XC_RS17885 | D | CCCCGTCC | -10 | -3 | TCCCGTCC | 0.88 |
| XC_RS17885 | D | CCCGTCCC | -9 | -2 | CCCGTCCT | 0.88 |
| XC_RS17885 | D | CTCCCGTC | -11 | -4 | TTCCCGTC | 0.88 |
| XC_RS17885 | D | CCTCTCTCCC | -23 | -14 | CCCCTCTCCC | 0.9 |
| XC_RS17895 | D | CCCGTCCC | -26 | -19 | CCCGCCCC | 0.88 |
| XC_RS17910 | R | CTCCCGTC | -72 | -65 | CTCCCGCC | 0.88 |
| XC_RS17925 | D | CCCCTCTC | -26 | -19 | CCCCTCTC | 1 |
| XC_RS17925 | R | CCCCTCTC | -82 | -75 | CCCGTCTC | 0.88 |
| XC_RS17925 | R | CCCGTCCC | -82 | -75 | CCCGTCTC | 0.88 |
| XC_RS17925 | R | CTCCCGTC | -80 | -73 | TTCCCGTC | 0.88 |
| XC_RS17925 | D | CCTCTCTCCC | -26 | -17 | CCCCTCTCCC | 0.9 |
| XC_RS17930 | D | CCCGTCCC | -119 | -112 | CCCGTTCC | 0.88 |
| XC_RS17930 | D | CCCGTCCC | -64 | -57 | CCCGTTCC | 0.88 |
| XC_RS17935 | R | AGGCGGAGG | -140 | -132 | CGGCGGAGG | 0.89 |
| XC_RS17940 | D | AGGCGGAGG | -95 | -87 | CGGCGGAGG | 0.89 |
| XC_RS17950 | D | CCCCGTCC | -42 | -35 | TCCCGTCC | 0.88 |
| XC_RS17950 | D | CCCGTCCC | -41 | -34 | CCCGTCCT | 0.88 |
| XC_RS17950 | D | CTCCCGTC | -43 | -36 | CTCCCGTC | 1 |
| XC_RS23315 | R | CTCCCGTC | -29 | -22 | CTCCCGAC | 0.88 |
| XC_RS17970 | D | CCCCGTCC | -163 | -156 | CCGCGTCC | 0.88 |
| XC_RS17970 | D | CCCGTCCC | -33 | -26 | CCCGCCCC | 0.88 |
| XC_RS17990 | D | CCCCTCTC | -76 | -69 | CCCCTCAC | 0.88 |
| XC_RS17990 | D | CCCGTCCC | -162 | -155 | CCTGTCCC | 0.88 |
| XC_RS17995 | R | CCCGTCCC | -19 | -12 | CCCGTGCC | 0.88 |
| XC_RS17995 | D | CTCGCCGTC | -170 | -162 | CTGGCCGTC | 0.89 |
| XC_RS18010 | D | CCCCGTCC | -38 | -31 | CACCGTCC | 0.88 |
| XC_RS18015 | D | CTCCCGTC | -21 | -14 | CACCCGTC | 0.88 |
| XC_RS18075 | D | CCCGTCCC | -79 | -72 | CCCGACCC | 0.88 |
| XC_RS18080 | D | CCCCGTCC | -94 | -87 | CCCCGGCC | 0.88 |
| XC_RS18085 | R | CCCGTCCC | -98 | -91 | CCCGCCCC | 0.88 |
| XC_RS18090 | D | CCCGTCCC | -105 | -98 | CCCGGCCC | 0.88 |
| XC_RS18100 | D | CCCGTCCC | -12 | -5 | CCCGTCGC | 0.88 |
| XC_RS18100 | D | CTCCCGTC | -14 | -7 | CGCCCGTC | 0.88 |
| XC_RS18105 | R | CCCGTCCC | -127 | -120 | CCCGTCGC | 0.88 |
| XC_RS18110 | D | CTCCCGTC | -186 | -179 | CTCCCGAC | 0.88 |
| XC_RS18115 | D | CTCCCGTC | -37 | -30 | CTACCGTC | 0.88 |
| XC_RS18120 | R | CTCCCGTC | -58 | -51 | CTACCGTC | 0.88 |
| XC_RS18145 | D | CCCCTCTC | -24 | -17 | CTCCTCTC | 0.88 |
| XC_RS18155 | D | CCCCTCTC | -90 | -83 | CCCATCTC | 0.88 |
| XC_RS18165 | R | CCCGTCCC | -101 | -94 | CCAGTCCC | 0.88 |
| XC_RS18200 | D | CCCCTCTC | -25 | -18 | TCCCTCTC | 0.88 |
| XC_RS18230 | D | CTCCCGTC | -112 | -105 | CTCCCGTT | 0.88 |
| XC_RS18235 | R | CTCCCGTC | -15 | -8 | CTCCCGTT | 0.88 |
| XC_RS18250 | R | CCCCTCTC | -10 | -3 | CCGCTCTC | 0.88 |
| XC_RS18250 | R | CCCCGTCC | -59 | -52 | CCCTGTCC | 0.88 |
| XC_RS18255 | D | CCCCTCTC | -132 | -125 | CCGCTCTC | 0.88 |
| XC_RS18255 | D | CCCCGTCC | -83 | -76 | CCCTGTCC | 0.88 |
| XC_RS18260 | D | CCCCTCTC | -8 | -1 | CCCCGCTC | 0.88 |
| XC_RS23340 | D | CTCCCGTC | -67 | -60 | CTCCCGTA | 0.88 |
| XC_RS18350 | D | CCCCGTCC | -58 | -51 | CCCGGTCC | 0.88 |
| XC_RS18395 | D | CCCCGTCC | -167 | -160 | CACCGTCC | 0.88 |
| XC_RS18400 | R | CCCGTCCC | -91 | -84 | CCCGGCCC | 0.88 |
| XC_RS18400 | R | AGGCGGAGG | -112 | -104 | AGGCGGCGG | 0.89 |
| XC_RS18480 | R | CCCGTCCC | -120 | -113 | CCGGTCCC | 0.88 |
| XC_RS18485 | D | CCCCTCTC | -26 | -19 | CGCCTCTC | 0.88 |
| XC_RS18485 | D | CCTCTCTCCC | -24 | -15 | CCTCTCTCGC | 0.9 |
| XC_RS18490 | R | CCCCTCTC | -114 | -107 | CCTCTCTC | 0.88 |
| XC_RS18490 | R | CCTCTCTCCC | -116 | -107 | CCTCTCTCGC | 0.9 |
| XC_RS18495 | R | CTCCCGTC | -92 | -85 | CTGCCGTC | 0.88 |
| XC_RS18505 | D | CCCCGTCC | -24 | -17 | CCCCATCC | 0.88 |
| XC_RS23365 | R | CCCCGTCC | -26 | -19 | CCGCGTCC | 0.88 |
| XC_RS23365 | D | AGGCGGAGG | -30 | -22 | AGGCGGACG | 0.89 |
| XC_RS18520 | R | CCCGTCCC | -192 | -185 | GCCGTCCC | 0.88 |
| XC_RS18555 | D | CCCGTCCC | -55 | -48 | CCCGTCGC | 0.88 |
| XC_RS23370 | R | CCCCGTCC | -35 | -28 | CCCCGCCC | 0.88 |
| XC_RS18600 | D | CCCCTCTC | -44 | -37 | CCCCACTC | 0.88 |
| XC_RS18610 | D | CCCCTCTC | -48 | -41 | CCCCACTC | 0.88 |
| XC_RS18640 | D | CTCCCGTC | -145 | -138 | CTCCCGTT | 0.88 |
| XC_RS18665 | D | CTCCCGTC | -52 | -45 | CTCCCGGC | 0.88 |
| XC_RS18705 | R | CCCCGTCC | -149 | -142 | CCCCGTGC | 0.88 |
| XC_RS18705 | R | AGGCGGAGG | -24 | -16 | AGGAGGAGG | 0.89 |
| XC_RS18715 | R | CCCGTCCC | -115 | -108 | CCAGTCCC | 0.88 |
| XC_RS18720 | R | CCCGTCCC | -162 | -155 | CCCATCCC | 0.88 |
| XC_RS18725 | D | CCCCTCTC | -62 | -55 | CCCCTGTC | 0.88 |
| XC_RS18725 | D | CCCGTCCC | -103 | -96 | CCCATCCC | 0.88 |
| XC_RS18740 | D | CTCCCGTC | -41 | -34 | CTCCCGTG | 0.88 |
| XC_RS18760 | D | CCCGTCCC | -43 | -36 | CCGGTCCC | 0.88 |
| XC_RS18765 | R | CCCGTCCC | -112 | -105 | CCGGTCCC | 0.88 |
| XC_RS18780 | D | CCCGTCCC | -81 | -74 | CCCGTCGC | 0.88 |
| XC_RS18780 | D | CTCCCGTC | -83 | -76 | CGCCCGTC | 0.88 |
| XC_RS18825 | R | CTCCCGTC | -15 | -8 | CTCCTGTC | 0.88 |
| XC_RS18830 | D | CTCCCGTC | -14 | -7 | CTCCCGGC | 0.88 |
| XC_RS23390 | R | CTCCCGTC | -47 | -40 | CTTCCGTC | 0.88 |
| XC_RS18865 | D | CTCCCGTC | -60 | -53 | CTCCCATC | 0.88 |
| XC_RS18865 | R | CTCCCGTC | -88 | -81 | CTCCCGAC | 0.88 |
| XC_RS18880 | D | CCCCGTCC | -62 | -55 | CCCTGTCC | 0.88 |
| XC_RS18880 | D | CTCCCGTC | -93 | -86 | CTCCAGTC | 0.88 |
| XC_RS18885 | R | CTCCCGTC | -61 | -54 | CTCTCGTC | 0.88 |
| XC_RS23395 | D | CTCCCGTC | -91 | -84 | CTCTCGTC | 0.88 |
| XC_RS18910 | D | CCCCTCTC | -33 | -26 | GCCCTCTC | 0.88 |
| XC_RS18910 | D | CCTCTCTCCC | -31 | -22 | CCTCTCACCC | 0.9 |
| XC_RS18920 | D | CCCCGTCC | -84 | -77 | ACCCGTCC | 0.88 |
| XC_RS18920 | D | CCCGTCCC | -83 | -76 | CCCGTCCT | 0.88 |
| XC_RS18935 | R | CTCGCCGTC | -73 | -65 | CTTGCCGTC | 0.89 |
| XC_RS18940 | D | CTCCCGTC | -65 | -58 | ATCCCGTC | 0.88 |
| XC_RS18955 | R | CCCCGTCC | -172 | -165 | CCGCGTCC | 0.88 |
| XC_RS18955 | D | CTCCCGTC | -26 | -19 | CTGCCGTC | 0.88 |
| XC_RS18955 | D | CTCGCCGTC | -34 | -26 | CTCGCCGCC | 0.89 |
| XC_RS23405 | R | CCCCGTCC | -26 | -19 | CCGCGTCC | 0.88 |
| XC_RS18975 | D | CCCCGTCC | -36 | -29 | CCCCATCC | 0.88 |
| XC_RS18975 | D | CCCGTCCC | -35 | -28 | CCCATCCC | 0.88 |
| XC_RS18980 | D | CCCCGTCC | -62 | -55 | CCGCGTCC | 0.88 |
| XC_RS18980 | D | CCCGTCCC | -115 | -108 | CCCGCCCC | 0.88 |
| XC_RS19010 | D | CTCGCCGTC | -41 | -33 | CGCGCCGTC | 0.89 |
| XC_RS19015 | D | CTCCCGTC | -115 | -108 | CTCCCGCC | 0.88 |
| XC_RS19035 | R | CCCCGTCC | -62 | -55 | CCCCGTCA | 0.88 |
| XC_RS19040 | D | CCCCGTCC | -18 | -11 | CCCCGTCA | 0.88 |
| XC_RS19050 | D | CCCCGTCC | -26 | -19 | CCCCTTCC | 0.88 |
| XC_RS19050 | D | CCCGTCCC | -25 | -18 | CCCTTCCC | 0.88 |
| XC_RS19050 | D | CCCCTTCTCC | -26 | -17 | CCCCTTCCCC | 0.9 |
| XC_RS19075 | R | CTCCCGTC | -49 | -42 | CTGCCGTC | 0.88 |
| XC_RS23410 | D | CTCGCCGTC | -95 | -87 | CTCGCCGTT | 0.89 |
| XC_RS19100 | R | CCCCGTCC | -32 | -25 | CCCCGGCC | 0.88 |
| XC_RS19100 | R | CCCGTCCC | -33 | -26 | CCCGGCCC | 0.88 |
| XC_RS19105 | D | CCCCGTCC | -25 | -18 | GCCCGTCC | 0.88 |
| XC_RS19105 | D | CCCGTCCC | -24 | -17 | CCCGTCCC | 1 |
| XC_RS19105 | D | CTCCCGTC | -26 | -19 | CGCCCGTC | 0.88 |
| XC_RS19110 | D | CCCCTCTC | -200 | -193 | CCCCTCCC | 0.88 |
| XC_RS19110 | D | CCCCTCTC | -189 | -182 | CCCCTCCC | 0.88 |
| XC_RS19110 | D | CCCCTCTC | -178 | -171 | CCCCTCCC | 0.88 |
| XC_RS19110 | D | CCCCGTCC | -190 | -183 | CCCCCTCC | 0.88 |
| XC_RS19110 | D | CCCCGTCC | -179 | -172 | CCCCCTCC | 0.88 |
| XC_RS19110 | D | CCCCGTCC | -88 | -81 | CCCCCTCC | 0.88 |
| XC_RS19110 | D | CCCGTCCC | -200 | -193 | CCCCTCCC | 0.88 |
| XC_RS19110 | D | CCCGTCCC | -189 | -182 | CCCCTCCC | 0.88 |
| XC_RS19110 | D | CCCGTCCC | -178 | -171 | CCCCTCCC | 0.88 |
| XC_RS19110 | D | CTCCCGTC | -197 | -190 | CTCCCATC | 0.88 |
| XC_RS19110 | D | CTCCCGTC | -186 | -179 | CTCCCATC | 0.88 |
| XC_RS19110 | D | CTCCCGTC | -175 | -168 | CTCCCATC | 0.88 |
| XC_RS19150 | D | CTCGCCGTC | -100 | -92 | CTCGCCGGC | 0.89 |
| XC_RS19165 | R | AGGCGGAGG | -66 | -58 | AGGCGAAGG | 0.89 |
| XC_RS19205 | R | CCCGTCCC | -90 | -83 | CCCGTCGC | 0.88 |
| XC_RS19215 | D | CCCGTCCC | -23 | -16 | CCAGTCCC | 0.88 |
| XC_RS19235 | R | CTCCCGTC | -69 | -62 | CTCCCGTT | 0.88 |
| XC_RS19235 | R | CTCGCCGTC | -15 | -7 | CTCGCTGTC | 0.89 |
| XC_RS19255 | R | CTCCCGTC | -148 | -141 | CTCCCGGC | 0.88 |
| XC_RS19260 | D | CTCCCGTC | -105 | -98 | CTCCCGGC | 0.88 |
| XC_RS19265 | D | CCCCCGCCTC | -54 | -45 | CCCCCGCTTC | 0.9 |
| XC_RS19300 | D | CCCCGTCC | -64 | -57 | CACCGTCC | 0.88 |
| XC_RS19300 | D | CCCGTCCC | -63 | -56 | ACCGTCCC | 0.88 |
| XC_RS19320 | D | AGGCGGAGG | -13 | -5 | AGGCCGAGG | 0.89 |
| XC_RS19325 | R | CCCCTCTC | -14 | -7 | CCCCTCGC | 0.88 |
| XC_RS19325 | R | CCCCGTCC | -8 | -1 | CCCCGTCC | 1 |
| XC_RS19325 | R | CCCGTCCC | -9 | -2 | CCCGTCCC | 1 |
| XC_RS19335 | D | CTCGCCGTC | -147 | -139 | CTCGCCGTG | 0.89 |
| XC_RS19345 | R | AGGCGGAGG | -84 | -76 | AGGCGGCGG | 0.89 |
| XC_RS19360 | D | CCCCGTCC | -69 | -62 | CCCCGGCC | 0.88 |
| XC_RS19365 | R | CCCCGTCC | -43 | -36 | CCCCGGCC | 0.88 |
| XC_RS19390 | R | CCCCGTCC | -24 | -17 | CCCTGTCC | 0.88 |
| XC_RS19455 | D | CCCCGTCC | -50 | -43 | CCTCGTCC | 0.88 |
| XC_RS19480 | D | CCCCGTCC | -115 | -108 | CCGCGTCC | 0.88 |
| XC_RS19480 | D | CTCCCGTC | -28 | -21 | CTCCCGAC | 0.88 |
| XC_RS19480 | R | AGGCGGAGG | -179 | -171 | AGGCGGATG | 0.89 |
| XC_RS19490 | R | CCCCGTCC | -17 | -10 | CCCTGTCC | 0.88 |
| XC_RS19495 | R | CCCGTCCC | -118 | -111 | CACGTCCC | 0.88 |
| XC_RS19495 | R | CCCGTCCC | -103 | -96 | CCCGTCGC | 0.88 |
| XC_RS19495 | R | CCCCTTCTCC | -170 | -161 | CCGCTTCTCC | 0.9 |
| XC_RS19505 | D | CCCGTCCC | -26 | -19 | CCCGGCCC | 0.88 |
| XC_RS23435 | D | CCCCGTCC | -117 | -110 | GCCCGTCC | 0.88 |
| XC_RS23435 | D | CCCGTCCC | -116 | -109 | CCCGTCCC | 1 |
| XC_RS23435 | D | CTCCCGTC | -118 | -111 | CGCCCGTC | 0.88 |
| XC_RS19520 | R | CCCCGTCC | -97 | -90 | GCCCGTCC | 0.88 |
| XC_RS19520 | R | CCCGTCCC | -98 | -91 | CCCGTCCC | 1 |
| XC_RS19520 | R | CTCCCGTC | -96 | -89 | CGCCCGTC | 0.88 |
| XC_RS19540 | R | CTCGCCGTC | -53 | -45 | CTCGCCGAC | 0.89 |
| XC_RS19560 | D | CTCCCGTC | -63 | -56 | CTCCCGTT | 0.88 |
| XC_RS19560 | R | CTCCCGTC | -87 | -80 | CTCCCGTG | 0.88 |
| XC_RS19635 | D | CCCCTCTC | -35 | -28 | CCCCTCTC | 1 |
| XC_RS19635 | D | CCCCGTCC | -86 | -79 | CGCCGTCC | 0.88 |
| XC_RS19640 | R | CCCCTCTC | -143 | -136 | CCCCTCTC | 1 |
| XC_RS19640 | R | CCCCGTCC | -92 | -85 | CGCCGTCC | 0.88 |
| XC_RS19645 | D | CCCCGTCC | -145 | -138 | CGCCGTCC | 0.88 |
| XC_RS19650 | D | CTCCCGTC | -86 | -79 | CTCCGGTC | 0.88 |
| XC_RS19670 | D | AGGCGGAGG | -37 | -29 | AGGCGCAGG | 0.89 |
| XC_RS19690 | D | CCCCTCTC | -68 | -61 | CGCCTCTC | 0.88 |
| XC_RS19770 | D | CTCCCGTC | -37 | -30 | CACCCGTC | 0.88 |
| XC_RS19780 | R | CCCCGTCC | -69 | -62 | CCCCGCCC | 0.88 |
| XC_RS19785 | D | CCCCTCTC | -171 | -164 | CCCATCTC | 0.88 |
| XC_RS19830 | D | CCCCTCTC | -85 | -78 | CGCCTCTC | 0.88 |
| XC_RS19860 | R | CTCCCGTC | -199 | -192 | TTCCCGTC | 0.88 |
| XC_RS19865 | R | CCCGTCCC | -158 | -151 | GCCGTCCC | 0.88 |
| XC_RS19865 | R | CTCGCCGTC | -27 | -19 | TTCGCCGTC | 0.89 |
| XC_RS19870 | D | CCCGTCCC | -22 | -15 | GCCGTCCC | 0.88 |
| XC_RS19870 | D | CTCGCCGTC | -154 | -146 | TTCGCCGTC | 0.89 |
| XC_RS19885 | D | CCCCGTCC | -71 | -64 | CCCCGCCC | 0.88 |
| XC_RS19885 | D | AGGCGGAGG | -167 | -159 | AGGCGAAGG | 0.89 |
| XC_RS19900 | R | CCCCTCTC | -68 | -61 | CCCCTGTC | 0.88 |
| XC_RS19930 | R | AGGCGGAGG | -31 | -23 | AGGCGGAGC | 0.89 |
| XC_RS19940 | D | CCCCGTCC | -35 | -28 | CGCCGTCC | 0.88 |
| XC_RS19940 | R | AGGCGGAGG | -39 | -31 | CGGCGGAGG | 0.89 |
| XC_RS23470 | D | CTCCCGTC | -94 | -87 | CTTCCGTC | 0.88 |
| XC_RS23470 | R | CTCGCCGTC | -60 | -52 | CTCGCCGGC | 0.89 |
| XC_RS19955 | R | CCCCGTCC | -54 | -47 | CCCCGTTC | 0.88 |
| XC_RS19960 | D | CCCCGTCC | -50 | -43 | CCCCGTTC | 0.88 |
| XC_RS19970 | R | CCCCTTCTCC | -13 | -4 | CCACTTCTCC | 0.9 |
| XC_RS19980 | D | CCCCGTCC | -23 | -16 | CCACGTCC | 0.88 |
| XC_RS19980 | D | CCCGTCCC | -22 | -15 | CACGTCCC | 0.88 |
| XC_RS19985 | D | CTCCCGTC | -21 | -14 | CTCCCGAC | 0.88 |
| XC_RS19990 | R | CTCCCGTC | -44 | -37 | CTCCCGAC | 0.88 |
| XC_RS20005 | R | CCCCGTCC | -166 | -159 | CCCCGACC | 0.88 |
| XC_RS20005 | R | CCCGTCCC | -167 | -160 | CCCGACCC | 0.88 |
| XC_RS20005 | R | CTCCCGTC | -199 | -192 | CTCCCGCC | 0.88 |
| XC_RS20005 | R | CTCGCCGTC | -133 | -125 | CTGGCCGTC | 0.89 |
| XC_RS20005 | R | CCCCTTCTCC | -195 | -186 | CACCTTCTCC | 0.9 |
| XC_RS20010 | D | CCCCGTCC | -83 | -76 | CCCCGACC | 0.88 |
| XC_RS20010 | D | CCCGTCCC | -82 | -75 | CCCGACCC | 0.88 |
| XC_RS20010 | D | CTCCCGTC | -50 | -43 | CTCCCGCC | 0.88 |
| XC_RS20010 | R | CTCCCGTC | -45 | -38 | CTCCCGGC | 0.88 |
| XC_RS20010 | D | CTCGCCGTC | -117 | -109 | CTGGCCGTC | 0.89 |
| XC_RS20010 | D | CCCCTTCTCC | -56 | -47 | CACCTTCTCC | 0.9 |
| XC_RS20020 | D | CCCCGTCC | -76 | -69 | CCCCGTGC | 0.88 |
| XC_RS20025 | D | CTCCCGTC | -28 | -21 | CGCCCGTC | 0.88 |
| XC_RS20030 | R | CTCCCGTC | -170 | -163 | CGCCCGTC | 0.88 |
| XC_RS20050 | D | CCCCTCTC | -82 | -75 | CCACTCTC | 0.88 |
| XC_RS20050 | D | CCCCGTCC | -134 | -127 | CTCCGTCC | 0.88 |
| XC_RS20050 | D | CCCGTCCC | -133 | -126 | TCCGTCCC | 0.88 |
| XC_RS20060 | D | CCCCTCTC | -42 | -35 | CCCCTCTG | 0.88 |
| XC_RS20065 | R | CCCCTCTC | -41 | -34 | CCCCTCTG | 0.88 |
| XC_RS20120 | D | CCCCCGCCTC | -20 | -11 | CCCCCGCTTC | 0.9 |
| XC_RS20150 | D | CCCGTCCC | -60 | -53 | ACCGTCCC | 0.88 |
| XC_RS20185 | D | CTCGCCGTC | -25 | -17 | CTCGCCGGC | 0.89 |
| XC_RS20200 | D | CCCCTCTC | -27 | -20 | CACCTCTC | 0.88 |
| XC_RS20210 | R | CCCCTCTC | -134 | -127 | CCCTTCTC | 0.88 |
| XC_RS20210 | D | CCCGTCCC | -177 | -170 | CCCGTACC | 0.88 |
| XC_RS20210 | D | CTCCCGTC | -144 | -137 | CTCCCGGC | 0.88 |
| XC_RS20210 | R | CTCCCGTC | -139 | -132 | CTCCCGCC | 0.88 |
| XC_RS20210 | R | CCCCTTCTCC | -135 | -126 | TCCCTTCTCC | 0.9 |
| XC_RS20225 | D | CCCGTCCC | -102 | -95 | CCCGTCGC | 0.88 |
| XC_RS20225 | D | CTCCCGTC | -104 | -97 | CGCCCGTC | 0.88 |
| XC_RS20230 | D | CCCCGTCC | -116 | -109 | CCCCGTCA | 0.88 |
| XC_RS20230 | D | CCCCGTCC | -38 | -31 | CCCCGTCC | 1 |
| XC_RS20230 | D | CCCGTCCC | -115 | -108 | CCCGTCAC | 0.88 |
| XC_RS20230 | D | CCCGTCCC | -37 | -30 | CCCGTCCC | 1 |
| XC_RS20235 | D | CCCCGTCC | -17 | -10 | CCCCGTCA | 0.88 |
| XC_RS20250 | R | CCCCTCTC | -176 | -169 | CCCCACTC | 0.88 |
| XC_RS20250 | R | CTCCCGTC | -181 | -174 | CTCCCGGC | 0.88 |
| XC_RS23490 | R | CTCCCGTC | -28 | -21 | CGCCCGTC | 0.88 |
| XC_RS20290 | R | CCCCGTCC | -108 | -101 | GCCCGTCC | 0.88 |
| XC_RS20290 | R | CCCGTCCC | -109 | -102 | CCCGTCCC | 1 |
| XC_RS20305 | R | CCCCGTCC | -24 | -17 | CCGCGTCC | 0.88 |
| XC_RS20340 | R | CCCCGTCC | -74 | -67 | CCACGTCC | 0.88 |
| XC_RS20340 | R | CCCGTCCC | -75 | -68 | CACGTCCC | 0.88 |
| XC_RS20345 | D | CCCCGTCC | -30 | -23 | CCACGTCC | 0.88 |
| XC_RS20345 | D | CCCGTCCC | -29 | -22 | CACGTCCC | 0.88 |
| XC_RS20350 | D | CCCCTCTC | -88 | -81 | CCGCTCTC | 0.88 |
| XC_RS20350 | D | CCTCTCTCCC | -86 | -77 | GCTCTCTCCC | 0.9 |
| XC_RS20405 | D | CTCCCGTC | -194 | -187 | CTCCAGTC | 0.88 |
| XC_RS20430 | D | CCCCTCTC | -85 | -78 | CCCCTATC | 0.88 |
| XC_RS20465 | R | CTCCCGTC | -14 | -7 | CTCCTGTC | 0.88 |
| XC_RS23495 | R | AGGCGGAGG | -115 | -107 | CGGCGGAGG | 0.89 |
| XC_RS20480 | R | AGGCGGAGG | -27 | -19 | AGGCGGCGG | 0.89 |
| XC_RS20485 | D | CTCGCCGTC | -26 | -18 | TTCGCCGTC | 0.89 |
| XC_RS20505 | R | CTCCCGTC | -130 | -123 | CTCCCGAC | 0.88 |
| XC_RS20505 | D | CTCGCCGTC | -76 | -68 | CTTGCCGTC | 0.89 |
| XC_RS20590 | D | CCCCGTCC | -177 | -170 | CCCCATCC | 0.88 |
| XC_RS20590 | R | CCCGTCCC | -143 | -136 | CCCTTCCC | 0.88 |
| XC_RS20605 | D | CCCCTCTC | -49 | -42 | CCCCGCTC | 0.88 |
| XC_RS20610 | R | AGGCGGAGG | -109 | -101 | AGGCGCAGG | 0.89 |
| XC_RS20615 | D | CCCCGTCC | -43 | -36 | CCCCGTCC | 1 |
| XC_RS20615 | D | CCCGTCCC | -42 | -35 | CCCGTCCC | 1 |
| XC_RS20620 | D | CCCCGTCC | -104 | -97 | CCCCGTCA | 0.88 |
| XC_RS20620 | D | CCCCGTCC | -86 | -79 | CCCCATCC | 0.88 |
| XC_RS20620 | D | CCCCGTCC | -39 | -32 | CGCCGTCC | 0.88 |
| XC_RS20620 | D | CCCGTCCC | -103 | -96 | CCCGTCAC | 0.88 |
| XC_RS20620 | D | CTCGCCGTC | -106 | -98 | CTCCCCGTC | 0.89 |
| XC_RS20620 | D | CTCGCCGTC | -41 | -33 | GTCGCCGTC | 0.89 |
| XC_RS20625 | D | CCCCGTCC | -160 | -153 | CCGCGTCC | 0.88 |
| XC_RS20630 | R | CCCCGTCC | -151 | -144 | CCCCGTCG | 0.88 |
| XC_RS20640 | R | CCCCTCTC | -172 | -165 | CCCGTCTC | 0.88 |
| XC_RS20640 | R | CCCCGTCC | -112 | -105 | CCCCGCCC | 0.88 |
| XC_RS20640 | R | CCCGTCCC | -172 | -165 | CCCGTCTC | 0.88 |
| XC_RS20740 | R | CCCCGTCC | -162 | -155 | CCCCGTCC | 1 |
| XC_RS20740 | R | CCCGTCCC | -163 | -156 | CCCGTCCG | 0.88 |
| XC_RS20740 | R | AGGCGGAGG | -22 | -14 | AGGCTGAGG | 0.89 |
| XC_RS20745 | D | CCCCGTCC | -11 | -4 | CCCCGTCC | 1 |
| XC_RS20745 | D | CCCGTCCC | -10 | -3 | CCCGTCCG | 0.88 |
| XC_RS20745 | D | AGGCGGAGG | -152 | -144 | AGGCTGAGG | 0.89 |
| XC_RS20760 | D | CCCCGTCC | -26 | -19 | CCCCGTGC | 0.88 |
| XC_RS20810 | D | AGGCGGAGG | -16 | -8 | AGGCGGCGG | 0.89 |
| XC_RS20815 | D | CCCCTCTC | -152 | -145 | CCCCTCTC | 1 |
| XC_RS20815 | D | CCCCTCTC | -123 | -116 | CCCTTCTC | 0.88 |
| XC_RS20815 | R | CCCCTCTC | -138 | -131 | CCCCTCTC | 1 |
| XC_RS20815 | D | CCCCGTCC | -171 | -164 | CCCCGTAC | 0.88 |
| XC_RS20815 | D | CCCGTCCC | -170 | -163 | CCCGTACC | 0.88 |
| XC_RS20815 | D | CTCCCGTC | -147 | -140 | CTCCCGTA | 0.88 |
| XC_RS20815 | D | CTCCCGTC | -118 | -111 | CTCCCATC | 0.88 |
| XC_RS20815 | D | CCCCTTCTCC | -124 | -115 | TCCCTTCTCC | 0.9 |
| XC_RS20815 | R | CCCCTTCTCC | -109 | -100 | CACCTTCTCC | 0.9 |
| XC_RS20815 | D | CCTCTCTCCC | -152 | -143 | CCCCTCTCCC | 0.9 |
| XC_RS20845 | R | CCCCGTCC | -195 | -188 | CCCCGGCC | 0.88 |
| XC_RS20855 | R | CCCCGTCC | -141 | -134 | CGCCGTCC | 0.88 |
| XC_RS20885 | D | CCCCGTCC | -69 | -62 | CCCCGGCC | 0.88 |
| XC_RS20885 | D | CTCCCGTC | -152 | -145 | CACCCGTC | 0.88 |
| XC_RS20885 | D | CCCCCGCCTC | -69 | -60 | CCCCGGCCTC | 0.9 |
| XC_RS20900 | R | CCCGTCCC | -134 | -127 | CGCGTCCC | 0.88 |
| XC_RS20905 | D | CCCCTCTC | -139 | -132 | CCCCACTC | 0.88 |
| XC_RS20910 | R | CCCCTCTC | -12 | -5 | CCACTCTC | 0.88 |
| XC_RS20920 | R | CCCCGTCC | -46 | -39 | CCTCGTCC | 0.88 |
| XC_RS20925 | D | AGGCGGAGG | -42 | -34 | AGGCGGATG | 0.89 |
| XC_RS20930 | R | CTCCCGTC | -95 | -88 | CTCGCGTC | 0.88 |
| XC_RS20940 | R | CCCCGTCC | -195 | -188 | CCGCGTCC | 0.88 |
| XC_RS20940 | D | CTCCCGTC | -14 | -7 | CACCCGTC | 0.88 |
| XC_RS20950 | R | CCCCGTCC | -68 | -61 | CCCCGCCC | 0.88 |
| XC_RS20955 | D | CCCCGTCC | -17 | -10 | CCCCGCCC | 0.88 |
| XC_RS20985 | R | CCCCTCTC | -37 | -30 | CCCCTCCC | 0.88 |
| XC_RS20985 | D | CCCCGTCC | -64 | -57 | CGCCGTCC | 0.88 |
| XC_RS20985 | R | CCCCGTCC | -36 | -29 | CCCCCTCC | 0.88 |
| XC_RS20985 | R | CCCGTCCC | -37 | -30 | CCCCTCCC | 0.88 |
| XC_RS20985 | R | AGGCGGAGG | -78 | -70 | AGGCGGAGC | 0.89 |
| XC_RS20985 | D | CTCGCCGTC | -66 | -58 | GTCGCCGTC | 0.89 |
| XC_RS20995 | D | CCCCTCTC | -106 | -99 | CCCCGCTC | 0.88 |
| XC_RS20995 | D | CCCCTCTC | -26 | -19 | CCCCACTC | 0.88 |
| XC_RS20995 | R | CCCCTCTC | -195 | -188 | CCGCTCTC | 0.88 |
| XC_RS20995 | D | CCCGTCCC | -12 | -5 | CCCGGCCC | 0.88 |
| XC_RS20995 | D | CTCCCGTC | -101 | -94 | CTCCCGCC | 0.88 |
| XC_RS20995 | R | AGGCGGAGG | -98 | -90 | AGGCGGCGG | 0.89 |
| XC_RS21000 | R | CTCGCCGTC | -58 | -50 | CACGCCGTC | 0.89 |
| XC_RS21015 | R | CCCGTCCC | -177 | -170 | CCCGGCCC | 0.88 |
| XC_RS21020 | D | CCCGTCCC | -101 | -94 | CCCGGCCC | 0.88 |
| XC_RS21035 | D | CCCCGTCC | -76 | -69 | CCCCATCC | 0.88 |
| XC_RS21035 | R | CCCCGTCC | -131 | -124 | CCGCGTCC | 0.88 |
| XC_RS21040 | D | CCCCTCTC | -111 | -104 | CCCCTCTC | 1 |
| XC_RS21040 | R | CCCCTCTC | -96 | -89 | CCCCTCTC | 1 |
| XC_RS21040 | R | AGGCGGAGG | -76 | -68 | AGGCGGAGA | 0.89 |
| XC_RS21040 | D | CCTCTCTCCC | -111 | -102 | CCCCTCTCCC | 0.9 |
| XC_RS21040 | R | CCTCTCTCCC | -98 | -89 | CCCCTCTCCC | 0.9 |
| XC_RS21050 | R | CCCCTCTC | -17 | -10 | CCCCTCCC | 0.88 |
| XC_RS21050 | R | CCCGTCCC | -17 | -10 | CCCCTCCC | 0.88 |
| XC_RS21050 | R | CTCCCGTC | -15 | -8 | CTCCCCTC | 0.88 |
| XC_RS21055 | D | CCCCTCTC | -170 | -163 | CCCCTCCC | 0.88 |
| XC_RS21055 | D | CCCGTCCC | -170 | -163 | CCCCTCCC | 0.88 |
| XC_RS21055 | D | CTCCCGTC | -172 | -165 | CTCCCCTC | 0.88 |
| XC_RS21065 | D | CCCGTCCC | -26 | -19 | CCCGTCGC | 0.88 |
| XC_RS21065 | D | CTCCCGTC | -28 | -21 | GTCCCGTC | 0.88 |
| XC_RS21075 | D | CCCCTCTC | -30 | -23 | CCCCCCTC | 0.88 |
| XC_RS21085 | R | CCCCGTCC | -191 | -184 | CCGCGTCC | 0.88 |
| XC_RS21090 | D | CCCCGTCC | -32 | -25 | CCGCGTCC | 0.88 |
| XC_RS21095 | D | CCCCGTCC | -29 | -22 | CCCAGTCC | 0.88 |
| XC_RS21120 | R | CCCCGTCC | -148 | -141 | CCCTGTCC | 0.88 |
| XC_RS21125 | D | CCCCTCTC | -151 | -144 | GCCCTCTC | 0.88 |
| XC_RS21125 | R | CCCCTCTC | -136 | -129 | CCCCTCTT | 0.88 |
| XC_RS21125 | R | CCCCTCTC | -30 | -23 | CCCTTCTC | 0.88 |
| XC_RS21125 | R | CCCCGTCC | -117 | -110 | CCCCGTAC | 0.88 |
| XC_RS21125 | D | CCCGTCCC | -73 | -66 | CCCGTACC | 0.88 |
| XC_RS21125 | R | CCCGTCCC | -118 | -111 | CCCGTACC | 0.88 |
| XC_RS21125 | D | CTCCCGTC | -40 | -33 | CTCCCGAC | 0.88 |
| XC_RS21125 | R | CTCCCGTC | -172 | -165 | CTCCCATC | 0.88 |
| XC_RS21125 | R | CTCCCGTC | -35 | -28 | CTCCCGTC | 1 |
| XC_RS21125 | D | CCCCTTCTCC | -183 | -174 | CACCTTCTCC | 0.9 |
| XC_RS21125 | D | CCCCTTCTCC | -46 | -37 | CACCTTCTCC | 0.9 |
| XC_RS21125 | R | CCCCTTCTCC | -31 | -22 | TCCCTTCTCC | 0.9 |
| XC_RS21130 | D | CCCCGTCC | -24 | -17 | CCCCTTCC | 0.88 |
| XC_RS21135 | R | CCCCGTCC | -75 | -68 | CCCCTTCC | 0.88 |
| XC_RS21140 | R | AGGCGGAGG | -142 | -134 | AGGCGGGGG | 0.89 |
| XC_RS21140 | D | CCCCCGCCTC | -142 | -133 | CCCCCGCCTA | 0.9 |
| XC_RS21190 | D | CCTCTCTCCC | -44 | -35 | CCTCACTCCC | 0.9 |
| XC_RS21200 | R | CCCCTCTC | -29 | -22 | GCCCTCTC | 0.88 |
| XC_RS21205 | D | CCCCTCTC | -29 | -22 | GCCCTCTC | 0.88 |
| XC_RS21240 | D | CCCCTCTC | -85 | -78 | CCCCTCTC | 1 |
| XC_RS21240 | R | CCCCTCTC | -70 | -63 | CCCCTCTC | 1 |
| XC_RS21240 | D | CTCCCGTC | -80 | -73 | CTCCCGTC | 1 |
| XC_RS21240 | R | CTCCCGTC | -75 | -68 | CTCCCGAC | 0.88 |
| XC_RS21240 | D | CCTCTCTCCC | -85 | -76 | CCCCTCTCCC | 0.9 |
| XC_RS21240 | R | CCTCTCTCCC | -72 | -63 | CCCCTCTCCC | 0.9 |
| XC_RS21245 | R | CCCGTCCC | -18 | -11 | CCCTTCCC | 0.88 |
| XC_RS21245 | R | CTCCCGTC | -16 | -9 | CTCCCTTC | 0.88 |
| XC_RS23515 | R | CTCCCGTC | -194 | -187 | CTCCTGTC | 0.88 |
| XC_RS21290 | D | CCCCTCTC | -21 | -14 | CCACTCTC | 0.88 |
| XC_RS21315 | R | CCCGTCCC | -74 | -67 | CCCTTCCC | 0.88 |
| XC_RS21325 | D | CCCCTCTC | -33 | -26 | CCCCTCCC | 0.88 |
| XC_RS21325 | D | CCCCTCTC | -24 | -17 | TCCCTCTC | 0.88 |
| XC_RS21325 | D | CCCGTCCC | -33 | -26 | CCCCTCCC | 0.88 |
| XC_RS21325 | D | CTCCCGTC | -30 | -23 | CTCCCCTC | 0.88 |
| XC_RS21330 | D | CCCGTCCC | -98 | -91 | CCCGTCGC | 0.88 |
| XC_RS21335 | R | CCCCGTCC | -116 | -109 | CCGCGTCC | 0.88 |
| XC_RS21335 | R | CCCGTCCC | -117 | -110 | CGCGTCCC | 0.88 |
| XC_RS21335 | R | CTCCCGTC | -120 | -113 | GTCCCGTC | 0.88 |
| XC_RS21340 | D | AGGCGGAGG | -103 | -95 | CGGCGGAGG | 0.89 |
| XC_RS21345 | R | AGGCGGAGG | -45 | -37 | CGGCGGAGG | 0.89 |
| XC_RS23520 | D | CCCCTCTC | -175 | -168 | CCCCTTTC | 0.88 |
| XC_RS23520 | R | CCCCTCTC | -160 | -153 | CCCCTCTC | 1 |
| XC_RS23520 | R | CCTCTCTCCC | -162 | -153 | CCCCTCTCCC | 0.9 |
| XC_RS21350 | D | CCCCTCTC | -34 | -27 | CCCTTCTC | 0.88 |
| XC_RS21350 | D | CCCCTTCTCC | -35 | -26 | TCCCTTCTCC | 0.9 |
| XC_RS21350 | R | CCCCTTCTCC | -22 | -13 | CACCTTCTCC | 0.9 |
| XC_RS21355 | D | CCCCGTCC | -153 | -146 | CCCCGTGC | 0.88 |
| XC_RS21355 | D | CCCGTCCC | -152 | -145 | CCCGTGCC | 0.88 |
| XC_RS21355 | D | CCCGTCCC | -121 | -114 | CCTGTCCC | 0.88 |
| XC_RS21355 | R | CTCGCCGTC | -175 | -167 | ATCGCCGTC | 0.89 |
| XC_RS21365 | R | CCCCGTCC | -112 | -105 | CCCCATCC | 0.88 |
| XC_RS21370 | R | CCCCTCTC | -14 | -7 | CCCCTCCC | 0.88 |
| XC_RS21370 | R | CCCCGTCC | -13 | -6 | CCCCCTCC | 0.88 |
| XC_RS21370 | R | CCCGTCCC | -14 | -7 | CCCCTCCC | 0.88 |
| XC_RS21375 | R | CCCCTCTC | -15 | -8 | CCCCTCCC | 0.88 |
| XC_RS21375 | R | CCCCGTCC | -14 | -7 | CCCCCTCC | 0.88 |
| XC_RS21375 | R | CCCGTCCC | -15 | -8 | CCCCTCCC | 0.88 |
| XC_RS21380 | D | CCCCGTCC | -50 | -43 | CCCCGTTC | 0.88 |
| XC_RS21380 | R | CCCCGTCC | -66 | -59 | CCCCGTTC | 0.88 |
| XC_RS21390 | R | CTCCCGTC | -193 | -186 | CTGCCGTC | 0.88 |
| XC_RS21395 | R | CCCGTCCC | -49 | -42 | CCCGTGCC | 0.88 |
| XC_RS21395 | D | CTCCCGTC | -179 | -172 | CTGCCGTC | 0.88 |
| XC_RS21450 | D | CTCCCGTC | -34 | -27 | CTCCGGTC | 0.88 |
| XC_RS21450 | R | CTCGCCGTC | -127 | -119 | CTAGCCGTC | 0.89 |
| XC_RS21475 | R | CTCCCGTC | -199 | -192 | TTCCCGTC | 0.88 |
| XC_RS21495 | R | CCCCTCTC | -189 | -182 | GCCCTCTC | 0.88 |
| XC_RS21495 | R | CCCCGTCC | -90 | -83 | CCCCGTCC | 1 |
| XC_RS21495 | R | CCCGTCCC | -91 | -84 | CCCGTCCC | 1 |
| XC_RS21495 | R | CTCCCGTC | -194 | -187 | CTCCCGCC | 0.88 |
| XC_RS21495 | R | CCCCTTCTCC | -120 | -111 | CACCTTCTCC | 0.9 |
| XC_RS23680 | D | CCCCGTCC | -93 | -86 | CCCCGGCC | 0.88 |
| XC_RS21505 | R | CCCCGTCC | -10 | -3 | TCCCGTCC | 0.88 |
| XC_RS21505 | R | CCCGTCCC | -11 | -4 | CCCGTCCC | 1 |
| XC_RS21505 | R | CTCCCGTC | -9 | -2 | ATCCCGTC | 0.88 |
| XC_RS21530 | D | CCCCGTCC | -150 | -143 | CCCCATCC | 0.88 |
| XC_RS21530 | D | CCCGTCCC | -128 | -121 | CTCGTCCC | 0.88 |
| XC_RS21545 | D | CCCCGTCC | -17 | -10 | CCCCGTCC | 1 |
| XC_RS21545 | D | CCCGTCCC | -16 | -9 | CCCGTCCT | 0.88 |
| XC_RS21550 | R | CCCCGTCC | -97 | -90 | CCCCGTCC | 1 |
| XC_RS21550 | R | CCCGTCCC | -98 | -91 | CCCGTCCT | 0.88 |
| XC_RS21555 | D | CCCGTCCC | -14 | -7 | CCCTTCCC | 0.88 |
| XC_RS21595 | R | CTCCCGTC | -63 | -56 | CTCCCGTT | 0.88 |
| XC_RS21595 | R | CTCGCCGTC | -75 | -67 | CGCGCCGTC | 0.89 |
| XC_RS21600 | R | CCCGTCCC | -59 | -52 | CCCGGCCC | 0.88 |
| XC_RS21605 | D | CCCGTCCC | -186 | -179 | CCCGGCCC | 0.88 |
| XC_RS21605 | D | AGGCGGAGG | -26 | -18 | AGGCGGCGG | 0.89 |
| XC_RS21615 | D | CTCGCCGTC | -180 | -172 | CTCGCCTTC | 0.89 |
| XC_RS21620 | R | CTCCCGTC | -61 | -54 | CTCCCGAC | 0.88 |
| XC_RS21675 | R | CCCCGTCC | -16 | -9 | CCCCGTTC | 0.88 |
| XC_RS21675 | R | CCCGTCCC | -17 | -10 | CCCGTTCC | 0.88 |
| XC_RS23585 | D | CTCGCCGTC | -131 | -123 | CTCGCCCTC | 0.89 |
| XC_RS21695 | R | AGGCGGAGG | -26 | -18 | TGGCGGAGG | 0.89 |
| XC_RS21700 | D | CTCCCGTC | -56 | -49 | CTCCCGTA | 0.88 |
| XC_RS21700 | R | CTCCCGTC | -16 | -9 | CTCCCGTT | 0.88 |
| XC_RS21700 | D | CCCCCGCCTC | -26 | -17 | CCCCAGCCTC | 0.9 |
| XC_RS21705 | D | CCCGTCCC | -115 | -108 | CCCGTCGC | 0.88 |
| XC_RS21705 | D | CTCCCGTC | -117 | -110 | CGCCCGTC | 0.88 |
| XC_RS21705 | R | AGGCGGAGG | -109 | -101 | AGGCGGAGC | 0.89 |
| XC_RS21710 | R | CTCCCGTC | -166 | -159 | CTCGCGTC | 0.88 |
| XC_RS21725 | D | CCCGTCCC | -187 | -180 | CCCGTCGC | 0.88 |
| XC_RS21755 | D | CCCCTCTC | -106 | -99 | CCCGTCTC | 0.88 |
| XC_RS21755 | D | CCCCGTCC | -107 | -100 | CCCCGTCT | 0.88 |
| XC_RS21755 | R | CCCCGTCC | -84 | -77 | CCCCGTGC | 0.88 |
| XC_RS21755 | D | CCCGTCCC | -106 | -99 | CCCGTCTC | 0.88 |
| XC_RS21760 | R | CCCCTCTC | -141 | -134 | CCCGTCTC | 0.88 |
| XC_RS21760 | D | CCCCGTCC | -163 | -156 | CCCCGTGC | 0.88 |
| XC_RS21760 | R | CCCCGTCC | -140 | -133 | CCCCGTCT | 0.88 |
| XC_RS21760 | R | CCCGTCCC | -141 | -134 | CCCGTCTC | 0.88 |
| XC_RS21800 | D | CTCCCGTC | -89 | -82 | CTCCCGAC | 0.88 |
| XC_RS21815 | D | CCCCGTCC | -42 | -35 | CCGCGTCC | 0.88 |
| XC_RS21825 | D | CCCGTCCC | -93 | -86 | CCCGTGCC | 0.88 |
| XC_RS21830 | D | CCCCTCTC | -26 | -19 | GCCCTCTC | 0.88 |
| XC_RS21830 | D | CCTCTCTCCC | -24 | -15 | CCTCTCCCCC | 0.9 |
| XC_RS21855 | D | CCCGTCCC | -32 | -25 | CCCGTGCC | 0.88 |
| XC_RS21875 | R | CCCCGTCC | -106 | -99 | CCCCGTGC | 0.88 |
| XC_RS21890 | D | CCCGTCCC | -72 | -65 | CCCGTCGC | 0.88 |
| XC_RS21890 | D | CTCCCGTC | -74 | -67 | ATCCCGTC | 0.88 |
| XC_RS21890 | R | CTCCCGTC | -88 | -81 | CTCCCGGC | 0.88 |
| XC_RS21895 | R | CCCCGTCC | -72 | -65 | CCCCGTCG | 0.88 |
| XC_RS21895 | R | CCCGTCCC | -73 | -66 | CCCGTCGC | 0.88 |
| XC_RS21895 | R | CCCCCGCCTC | -20 | -11 | CGCCCGCCTC | 0.9 |
| XC_RS21905 | R | CTCGCCGTC | -113 | -105 | CGCGCCGTC | 0.89 |
| XC_RS21910 | D | CTCGCCGTC | -91 | -83 | CGCGCCGTC | 0.89 |
| XC_RS21915 | R | CCCCTCTC | -9 | -2 | CCTCTCTC | 0.88 |
| XC_RS21915 | R | CCTCTCTCCC | -11 | -2 | CCTCTCTCTC | 0.9 |
| XC_RS21940 | D | CCCGTCCC | -64 | -57 | CCCGGCCC | 0.88 |

**Supplementary Note**

**Analysis Bind-n-seq data using macOS operating system**

1. For data analysis files were downloaded from website: <https://editor.wix.com/html/editor/web/renderer/edit/6bb6390a-86eb-4784-b407-ec128d2d7b5e?metaSiteId=c6f9b7de-1b51-41c3-be5f-c350090e0f8c&editorSessionId=0d4908cc-e087-4066-8ece-b62e9aaac012&referralInfo=dashboard> and files saved to a location on computer hard disk: mermade_v1.03.tar.gz (compressed archive file of the code needed to run), MERMADE, background.txt (random 21mers that acts as the default background for a MERMADE run), Bind-n-seq 13-barcodes.csv (a comma-separated list of the possible 3 long bar-codes), which can be edited in excel to add meaningful names for specific libraries against the barcodes. The downloaded MERMADE archive can be unpacked and archived using the following commands:

mv ~/Downloads/mermade_v1.03.tar.gz

tar –zxvf mermade_v1.03.tar.gz

2. To create (or add lines to) the file .bash_profile under user home directory by using following commands:

*pico .bash_profile*

*export MERMADE="${HOME}/mermade_v1.03"*

*export PATH="${MERMADE}:${MERMADE}/weblogo:${PATH}"*

*export PERL5LIB="${MERMADE}:${PERL5LIB}"*

Ctrl-X can be used to save the modified buffer, the Y key can be used to say yes and the return key to confirm the file name .bash_profile.

The command less .bash_profile can be used to test if the file has been generated successfully. Commands source .bash_profile and echo $MERMADE can be used to check the path to the MERMADE files. Run command run_mermade.pl can be used to test if the software has been installed successfully.

3. To create a new subdirectory with the command *mkdir directoryname* was used. The fastq.gz file was placed into this directory and then the *unzipped background.txt* file into the *mermade_v1.03* program directory (Note: that the input dataset should be compressed fastq.gz file contains all the sequence, it also must be the raw file from the sequencer without any pre-processing, which should not contain any sequencing adaptor sequences).

In the terminal window directories could be switched using the command *cd directoryname*. MERMADE could then be run with the command:

*run_mermade.pl –o databasename –v TGATCGGAAG sequencefile.fastq.gz*

*barcode.csv*

where *databasename* is the name of the database file ***sequencefile.fastq.gz*** is the name of the sequence file barcode.csv is the name of the edited barcode.csv file with user library names (Note there are other optional parameters can be further optimized by the user, but in general running the application at its default setting is recommended).

An analysis report was generated by using reporter.pl script. The reporter.pl script. was executable with command: *chmod +x/Users/x/mermade_v1.03/reporter.pl*

The analysis report was then generated with the command: *report.pl <database> <# of motifs><output dir><barcodes>* (Please note that it is essential to assign an output directory for saving the results)
