# Supplementary material for: An improved bind-n-seq strategy to determine protein-DNA interactions validated using the bacterial transcriptional regulator YipR
Source: BMC Microbiol. 2020 Jan 2;20:1. doi: 10.1186/s12866-019-1672-7 (PMC6941359; doi:10.1186/s12866-019-1672-7)
Supplement: Supplementary file 3 — Additional file 3: Table S2. Fragment used for EMSA experiment. [file 12866_2019_1672_MOESM3_ESM.docx]

**Supplementary Table S2. Fragment used for EMSA experiment**

| **Name** | **Sequence** |
| --- | --- |
| **XC_2633P** | 5′-CCG GCT TTA TCC AGA TCG CCG ACC TGC ACC CAG GAC CCA GCG GCT TGC TGA CGC CCT TCA AGG ACA CAC GGA TGT GGA TAG CGG GGT TCA AGC CCG ACG GGA ACC GCG ACA AGT AAC CCG GCC TCC TAC TTC CCA ATC CCT CTC-3′ |
| **Truncated XC_2633P** | 5′-CCG GCT TTA TCC AGA TCG CCG ACC TGC ACC CAG GAC CCA GCG GCT TGC TGA CGC CCT TCA AGG ACA CAC GGA TGT GGA TAG CGG GGT TCA AGC CCG ACG GGA ACC GCG ACA AGT AAC CCG GCC TCC TAC TTC CCA AT-3′ |
