# Supplementary material for: An improved bind-n-seq strategy to determine protein-DNA interactions validated using the bacterial transcriptional regulator YipR
Source: BMC Microbiol. 2020 Jan 2;20:1. doi: 10.1186/s12866-019-1672-7 (PMC6941359; doi:10.1186/s12866-019-1672-7)
Supplement: Supplementary file 4 — Additional file 4: Table S3. Barcode assignment for Bind-n-seq. [file 12866_2019_1672_MOESM4_ESM.docx]

**Supplementary Table S3. Barcode assignment for Bind-n-Seq**

| No. | Bar Code | Protein | Protein (nM) | Salt (mM) |
| --- | --- | --- | --- | --- |
| 1 | AAA | No-protein | 0 | 100 |
| 2 | AAC | Protein tested | 400 | 10 |
| 3 | AAG | Protein tested | 400 | 25 |
| 4 | AAT | Protein tested | 400 | 50 |
| 5 | ACA | Protein tested | 400 | 100 |
| 6 | ACC | Protein tested | 400 | 500 |
| 7 | ACG | Protein tested | 4 | 100 |
| 8 | ACT | Protein tested | 40 | 100 |
| 9 | AGA | Protein tested | 400 | 100 |
| 10 | AGC | Protein tested | 4000 | 100 |
| 11 | CCC | Oligo only | 0 | 0 |
