# Supplementary material for: An improved bind-n-seq strategy to determine protein-DNA interactions validated using the bacterial transcriptional regulator YipR
Source: BMC Microbiol. 2020 Jan 2;20:1. doi: 10.1186/s12866-019-1672-7 (PMC6941359; doi:10.1186/s12866-019-1672-7)
Supplement: Supplementary file 5 — Additional file 5: Table S4. Primers used in Bind-n-seq procedure. [file 12866_2019_1672_MOESM5_ESM.docx]

**Supplementary Table S4. Primers used in Bind-n-Seq procedure**

| **Oligo** | **Sequence** | **Note** |
| --- | --- | --- |
| **Bind-n-Seq 93 mer** | 5′-ACA CTC TTT CCC TAC ACG ACG CTC TTC CGA TCT AA**A AA**N NNN NNN NNN NNN NNN NNN NNT GAT CGG AAG AGC TCG TAT GCC GTC TTC TGC TTG-3′ | Barcode highlighted in bold (AAA). |
| **Primer 1** | 5′-CAA GCA GAA GAC GGC ATA CGA GCT CTT CCG ATC -3′ | Used in initial primer extension reaction. |
| **Primer 2** | 5′-AAT GAT ACG GCG ACC ACC GAG ATC TAC ACT CTT TCC CTA CAC GAC GCT CTT CCG ATC T-3′ | Used in PCR amplification reaction. |
| **Primer 3** | 5′-CAA GCA GAA GAC GGC ATA CGA GAT CGT GAT GTG ACT GGA GTT CAG ACG TGT GCT CTT CCG ATC TCA AGC AGA AGA CGG CAT ACG A-3′ | Used in PCR amplification reaction. |
