# Supplementary material for: An improved bind-n-seq strategy to determine protein-DNA interactions validated using the bacterial transcriptional regulator YipR
Source: BMC Microbiol. 2020 Jan 2;20:1. doi: 10.1186/s12866-019-1672-7 (PMC6941359; doi:10.1186/s12866-019-1672-7)
Supplement: Supplementary file 6 — Additional file 6: Table S5. Composition for KCl salt solutions. [file 12866_2019_1672_MOESM6_ESM.docx]

**Supplementary Table S5.** **Composition for** **KCl salt solutions**

| Solution | Volume | H_2_O | Total Volume | Final Con. |
| --- | --- | --- | --- | --- |
| KCl (2.5 M) | 9.9 ml | 0.1 ml | 10 ml | 2475 mM |
| KCl (2.5 M) | 1.9 ml | 8.1 ml | 10 ml | 475 mM |
| KCl (2.5 M) | 0.9 ml | 9.1 ml | 10 ml | 225 mM |
| KCl (2.5 M) | 0.4 ml | 9.6 ml | 10 ml | 100 mM |
| KCl (100 mM) | 2.5 ml | 7.5 ml | 10 ml | 25 mM |
