# Supplementary material for: An improved bind-n-seq strategy to determine protein-DNA interactions validated using the bacterial transcriptional regulator YipR
Source: BMC Microbiol. 2020 Jan 2;20:1. doi: 10.1186/s12866-019-1672-7 (PMC6941359; doi:10.1186/s12866-019-1672-7)
Supplement: Supplementary file 7 — Additional file 7: Table S6. Binding reaction conditions. [file 12866_2019_1672_MOESM7_ESM.docx]

**Supplementary Table S6. Binding reaction conditions**

| No. | Protein | Volume | Final | KCl | Volume | Final Con. |
| --- | --- | --- | --- | --- | --- | --- |
| 1 | No-protein | 5 µl | 0 nM | KCl (475mM) | 10 µl | 100 mM |
| 2 | Protein tested (4 µM) | 5 µl | 400 nM | KCl (25mM) | 10 µl | 10 mM |
| 3 | Protein tested (4 µM | 5 µl | 400 nM | KCl (100mM) | 10 µl | 25 mM |
| 4 | Protein tested (4 µM) | 5 µl | 400 nM | KCl (225mM) | 10 µl | 50 mM |
| 5 | Protein tested (4 µM) | 5 µl | 400 nM | KCl (475mM) | 10 µl | 100 mM |
| 6 | Protein tested (4 µM) | 5 µl | 400 nM | KCl (2475mM) | 10 µl | 500 mM |
| 7 | Protein tested (40 nM) | 5 µl | 4 nM | KCl (475mM) | 10 µl | 100 mM |
| 8 | Protein tested (400 nM) | 5 µl | 40nM | KCl (475mM) | 10 µl | 100 mM |
| 9 | Protein tested (4 µM) | 5 µl | 400nM | KCl (475mM) | 10 µl | 100 mM |
| 10 | Protein tested (40 µM) | 5 µl | 4000nM | KCl (475mM) | 10 µl | 100 mM |
