# Supplementary material for: An improved bind-n-seq strategy to determine protein-DNA interactions validated using the bacterial transcriptional regulator YipR
Source: BMC Microbiol. 2020 Jan 2;20:1. doi: 10.1186/s12866-019-1672-7 (PMC6941359; doi:10.1186/s12866-019-1672-7)
Supplement: Supplementary file 8 — Additional file 8: Table S7. Composition for Bin-n-seq wash buffer. [file 12866_2019_1672_MOESM8_ESM.docx]

**Supplementary Table S7. Composition for Bin-n-Seq wash buffer**

| **Bin-n-Seq wash buffer** |  |  |  |  | **×6** |  |
| --- | --- | --- | --- | --- | --- | --- |
| **KCl Final Con.** | 10 mM | 25 mM | 50mM | 100 nM | 100 nM | 500 nM |
| **H_2_O** | 8.5 ml | 8.5 ml | 8.5 ml | 8.5 ml | 51ml | 8.5 ml |
| **20×Binding Buffer A (Without KCl)** | 500 µl | 500 µl | 500 µl | 500 µl | 3000 µl | 500 µl |
| **MgCl_2_ (1M)** | 10 µl | 10 µl | 10 µl | 10 µl | 60 µl | 10 µl |
| **KCl (5M)** | 20 µl | 50 µl | 100 µl | 200 µl | 1200 µl | 1000 µl |
| **H_2_O** | 970 µl | 940 µl | 890 µl | 790 µl | 4740 µl | 0 µl |
| **Total Volume** | **10ml** | **10ml** | **10ml** | **10ml** | **60ml** | **10ml** |
