# Supplementary material for: An improved bind-n-seq strategy to determine protein-DNA interactions validated using the bacterial transcriptional regulator YipR
Source: BMC Microbiol. 2020 Jan 2;20:1. doi: 10.1186/s12866-019-1672-7 (PMC6941359; doi:10.1186/s12866-019-1672-7)
Supplement: Supplementary file 9 — Additional file 9. Analysis Bind-n-seq data using macOS operating system. [file 12866_2019_1672_MOESM9_ESM.docx]

**Supplementary Note**

**Analysis Bind-n-seq data using macOS operating system**

1. For data analysis files were downloaded from website: <https://anshiqi19840918.wixsite.com/ngsfilelinks/others> and files saved to a location on computer hard disk: mermade_v1.03.tar.gz (compressed archive file of the code needed to run), MERMADE, background.txt (random 21mers that acts as the default background for a MERMADE run), Bind-n-seq 13-barcodes.csv (a comma-separated list of the possible 3 long bar-codes), which can be edited in excel to add meaningful names for specific libraries against the barcodes. The downloaded MERMADE archive can be unpacked and archived using the following commands:

mv ~/Downloads/mermade_v1.03.tar.gz

tar –zxvf mermade_v1.03.tar.gz

2. To create (or add lines to) the file .bash_profile under user home directory by using following commands:

*pico .bash_profile*

*export MERMADE="${HOME}/mermade_v1.03"*

*export PATH="${MERMADE}:${MERMADE}/weblogo:${PATH}"*

*export PERL5LIB="${MERMADE}:${PERL5LIB}"*

Ctrl-X can be used to save the modified buffer, the Y key can be used to say yes and the return key to confirm the file name .bash_profile.

The command less .bash_profile can be used to test if the file has been generated successfully. Commands source .bash_profile and echo $MERMADE can be used to check the path to the MERMADE files. Run command run_mermade.pl can be used to test if the software has been installed successfully.

3. To create a new subdirectory with the command *mkdir directoryname* was used. The fastq.gz file was placed into this directory and then the *unzipped background.txt* file into the *mermade_v1.03* program directory (Note: that the input dataset should be compressed fastq.gz file contains all the sequence, it also must be the raw file from the sequencer without any pre-processing, which should not contain any sequencing adaptor sequences).

In the terminal window directories could be switched using the command *cd directoryname*. MERMADE could then be run with the command:

*run_mermade.pl –o databasename –v TGATCGGAAG sequencefile.fastq.gz*

*barcode.csv*

where *databasename* is the name of the database file ***sequencefile.fastq.gz*** is the name of the sequence file barcode.csv is the name of the edited barcode.csv file with user library names (Note there are other optional parameters can be further optimized by the user, but in general running the application at its default setting is recommended).

An analysis report was generated by using reporter.pl script. The reporter.pl script. was executable with command: *chmod +x/Users/x/mermade_v1.03/reporter.pl*

The analysis report was then generated with the command: *report.pl <database> <# of motifs><output dir><barcodes>* (Please note that it is essential to assign an output directory for saving the results)
